# Incidence and influencing factors of progressive pulmonary fibrosis in connective tissue disease-associated interstitial lung disease: a systematic review and meta-analysis

**DOI:** 10.3389/fimmu.2026.1758437

**Published:** 2026-02-04

**Authors:** Mingyang Yi, Minghao Li, Zhen Zhang, Haixia Li, Junting Sai, Yinzhe Gui, Zhiwan Wang

**Affiliations:** 1 Department of Respiratory Medicine, The First Affiliated Hospital of Henan University of Chinese Medicine, Zhengzhou, Henan, China; 2 The First Clinical Medical College, Henan University of Chinese Medicine, Zhengzhou, Henan, China; 3 Collaborative Innovation Center for Chinese Medicine and Respiratory Diseases Co-constructed by Henan Province & Ministry of Education of P.R. China, Henan University of Chinese Medicine, Zhengzhou, Henan, China

**Keywords:** connective tissue disease-associated interstitial lung disease, incidence, influencing factors, meta-analysis, progressive pulmonary fibrosis

## Abstract

**Background:**

Progressive pulmonary fibrosis (PPF) is a major cause of poor prognosis in connective tissue disease-associated interstitial lung disease (CTD-ILD). This study aims to analyze and evaluate the incidence of PPF and related influencing factors in CTD-ILD.

**Methods:**

We searched PubMed, EMBASE, the Cochrane Library, Web of Science, and Scopus databases for studies on the incidence of PPF and influencing factors in CTD-ILD until August 20, 2025. The methodological quality of the included studies was assessed using the Newcastle-Ottawa Scale (NOS). Meta-analysis was performed using Stata 17.0 software.

**Results:**

A total of 22 studies were included in the meta-analysis, comprising 20 high-quality studies and 2 medium-quality studies. The incidence of PPF in CTD-ILD was 29% (95% CI: 25% - 34%). Meta-analysis identified Krebs von den Lungen-6 (KL-6: OR = 2.21, 95% CI: 1.24 - 3.94), human Surfactant Protein D (hSP-D: OR = 1.48, 95% CI: 1.16 - 1.90), Matrix Metalloproteinase-7 (MMP-7: OR = 1.48, 95% CI: 1.13 - 1.93), and Cancer Antigen 125 (CA-125: OR = 1.19, 95% CI: 1.05 - 1.34) as risk factors for PPF development. A higher baseline forced vital capacity percentage predicted value (FVC% predicted: OR = 0.98, 95% CI: 0.96 - 0.99) was identified as a protective factor against PPF development.

**Conclusions:**

Patients with CTD-ILD are at high risk of developing PPF, and this progression risk is associated with KL-6, hSP-D, MMP-7, and CA-125. Identifying the incidence risk and influencing factors of PPF in CTD-ILD is crucial for early identification of high-risk populations, optimizing diagnostic and therapeutic strategies, and improving prognosis.

**Systematic Review Registration:**

https://www.crd.york.ac.uk/prospero/, identifier CRD420251128274.

## 1 Introduction

Connective tissue disease (CTD) represents a group of systemic autoimmune disorders characterized by immune-mediated organ dysfunction, encompassing subtypes such as systemic sclerosis (SSc), rheumatoid arthritis (RA), idiopathic inflammatory myopathy (IIM), Sjögren’s syndrome (SS), systemic lupus erythematosus (SLE), and mixed connective tissue disease (MCTD). The etiology remains incompletely understood, with most cases demonstrating a chronic progressive course that may affect joints, skin, and multiple internal organs (1). Interstitial lung disease (ILD) is a common and severe pulmonary complication of CTD, characterized by alveolar inflammation and interstitial fibrosis as core pathological changes. Its clinical manifestations include insidious dyspnea and dry cough, posing challenges for early diagnosis, and ultimately leading to irreversible pulmonary function impairment (2). The clinical outcomes of CTD-ILD exhibit significant heterogeneity, ranging from disease stability to progressive fibrosis culminating in respiratory failure and death. A subset of CTD-ILD patients develops a progressive fibrosing phenotype, predisposing them to irreversible pulmonary fibrosis (3). Even when the primary disease causing ILD has stabilized, pulmonary fibrosis may continue to progress. The median survival time for CTD-ILD patients is 8 to 10 years, but it decreases to 4 years for those who develop the PPF phenotype (4). The 2022 official ATS/ERS/JRS/ALAT clinical practice guideline defines PPF as meeting at least two of the following criteria within 12 months: worsening of respiratory symptoms, decline in physiological function, and increased fibrosis on high-resolution computed tomography (HRCT) (5). The guideline emphasizes that PPF is closely associated with disease prognosis. It serves to describe ‘progressive fibrotic ILD’ or ‘fibrotic ILD with a progressive phenotype’ characterized by a sharp decline in pulmonary function, worsening respiratory symptoms, and deterioration of health-related quality of life, which is significant for disease management.

Currently, there is no systematic research on the epidemiology of PPF in patients with CTD-ILD. Estimates can only be based on research data concerning the fibrosis progression phenotype within progressive fibrosing interstitial lung disease (PF-ILD) and rapidly progressive interstitial lung disease (RP-ILD). The 2022 guideline provides a detailed description of the morphological definition of fibrosis progression, assisting clinicians in determining the presence of progression. It also shortens the observation period to one year, enabling early disease identification and treatment to avoid delays in anti-fibrotic therapy, thus demonstrating strong practical significance. However, due to the relatively recent introduction of the PPF definition, current research in this field is limited. There are significant variations in study findings regarding the incidence of PPF and its influencing factors in CTD-ILD, which greatly hinders the implementation of clinical risk stratification and precise interventions. The 2025 joint ERS/EULAR clinical practice guideline for CTD-ILD noted that current evidence on the epidemiology and predictors of progressive fibrotic phenotypes in CTD-ILD is limited and requires systematic integration of existing research (6). Thus, this study quantitatively integrated the incidence of PPF and its influencing factors through systematic review and meta-analysis, to some extent addressing this evidence gap and providing a reliable basis for identifying high-risk populations and formulating individualized diagnosis and treatment strategies in clinical practice.

## 2 Methods

This study strictly adhered to the Meta-analysis of Observational Studies in Epidemiology (MOOSE) statement (7) and followed the Preferred Reporting Items for Systematic Reviews and Meta-Analyses (PRISMA) guidelines (8). The study protocol was pre-registered in the International Prospective Register of Systematic Reviews (PROSPERO) on August 19, 2025 (CRD420251128274).

### 2.1 Inclusion and exclusion criteria

We included English-language studies reporting the incidence of PPF and influencing factors in CTD-ILD patients. Studies meeting the following criteria were included: (1) Population (P): Adult patients diagnosed with CTD-ILD were eligible for inclusion; (2) Exposure (I): Clinical, demographic, radiological, physiological, serological, or treatment-related factors potentially associated with the development of PPF in CTD-ILD patients; (3) Comparison (C): CTD-ILD patients without the exposure of interest or who did not develop PPF during follow-up; (4) Outcomes (O): The primary outcome was the incidence of PPF, defined according to the 2022 clinical practice guideline, requiring worsening of respiratory symptoms, physiological progression, or radiological progression within a 12-month period. The secondary outcomes included influencing factors associated with PPF development, reported as odds ratio (OR), risk ratio (RR), or hazard ratio (HR), with corresponding 95% confidence intervals (CI); (5) Study Design (S): Cohort studies were used to assess incidence rates, while both cohort and case-control studies were utilized to evaluate influencing factors, depending on the research objectives.

We excluded the following studies: (1) Reviews, letters, case reports, conference abstracts, meta-analyses, and non-human studies; (2) Duplicate publications (only the version with the largest sample size or the most complete data was retained); (3) Studies with incomplete data that could not be obtained through reasonable means.

### 2.2 Search strategy

We developed a systematic literature search strategy after consulting with medical information technology specialists; details are provided in **Supplementary Table 1**. A comprehensive search was conducted in the PubMed, EMBASE, Cochrane Library, Web of Science, and Scopus databases for relevant articles published from inception to August 20, 2025. To maximize search accuracy, a combination of Medical Subject Headings (MeSH) terms and free-text keywords was employed, including: (“rheumatoid arthritis” OR “scleroderma” OR “sjogren syndrome” OR “myositis” OR “antisynthetase syndrome” OR “dermatomyositis” OR “polymyositis” OR “systemic lupus erythematosus” OR “mixed connective tissue disease” OR “connective tissue disease”) AND (“interstitial lung disease”) AND (“exacerbation” OR “symptom flare up” OR “disease progression” OR “clinical deterioration”). Precise Boolean operators (AND/OR) were used to construct the search syntax to identify studies related to the development of PPF in CTD-ILD. The search covered titles, abstracts, and keywords. Two investigators independently screened potentially relevant articles based on titles and abstracts. Full texts of eligible articles and their reference lists were subsequently reviewed to ensure comprehensiveness of the included literature. The eligibility of the included studies was assessed through discussion between two independent reviewers. In cases of disagreement, a third and fourth reviewer were consulted for consensus.

### 2.3 Data extraction and quality assessment

Data were independently extracted by two researchers and subsequently cross-verified to ensure accuracy and completeness. Standardized data extraction forms were used to collect data from the included studies. Extracted information included: author names, publication year, study country, research design, study duration, sample size, disease type, CTD subtypes, participant demographics (gender, age), outcome definitions (number of PPF cases, incidence of PPF, and influencing factors associated with outcomes). For influencing factors, priority was given to extracting adjusted OR values and 95% CI for confounding factors. We used the Newcastle-Ottawa Scale (NOS) as a tool to assess the quality of cohort studies and case-control studies. This scale comprises three dimensions, assessing subject selection, comparability within groups, and outcomes. The scale has a maximum score of 9 points, with studies scoring 0–3 points classified as low quality, 4–6 points as moderate quality, and 7–9 points as high quality. Two researchers independently assessed the quality of the included literature, with any disagreements discussed and resolved through consultation with a third researcher.

We complemented the quantitative NOS scoring with a structured narrative appraisal of bias domains most relevant to prognostic research. Specifically, we qualitatively evaluated: (1) selection bias, focusing on cohort representativeness, inclusion criteria, and loss to follow-up; (2) outcome ascertainment, including consistency with the 2022 ATS/ERS/JRS/ALAT definition of PPF and the objectivity of outcome assessment; (3) missing data, particularly the extent and handling of incomplete follow-up or biomarker measurements; and (4) adjustment adequacy, emphasizing whether multivariable models appropriately accounted for key confounders such as age, gender, baseline lung function, CTD subtypes, and treatment exposure. When multiple publications were suspected to originate from overlapping or identical cohorts, we compared study periods, institutions, and participant characteristics, and retained only the most informative dataset to avoid double-counting of participants in the meta-analysis.

### 2.4 Assessment of consistency with the PPF definition

Given the relatively recent introduction of the 2022 ATS/ERS/JRS/ALAT clinical practice guideline for PPF, we anticipated heterogeneity in how disease progression was operationalized across eligible studies. Therefore, in addition to extracting outcome data, we systematically recorded, for each included study, the observation window used to define progression, the PPF domains assessed (respiratory symptoms, physiological function, and/or HRCT), and the specific thresholds applied for physiological or radiological progression. These operational definitions are summarized in **Supplementary Table 3** to enhance transparency and facilitate interpretation of between-study heterogeneity.

### 2.5 Data synthesis and statistical analysis

Meta-analysis was performed using Stata 17.0 software. Incidence rates were expressed as pooled rates with 95% CI, and forest plots were generated. Subgroup analyses were conducted based on CTD subtypes, gender, age, and geographical region. Intergroup differences were assessed using chi-square tests. Heterogeneity was evaluated using Cochran’s Q-test and I² statistics. When no significant statistical heterogeneity existed among studies (I² ≤ 50%, P > 0.1), a fixed-effects model was applied; When statistical heterogeneity was present (I² > 50%, P < 0.1), sources of heterogeneity were investigated. After excluding significant clinical heterogeneity, a random-effects model was employed, and sensitivity analysis was performed to assess result stability (9). When more than ten studies were included in the meta-analysis, Egger’s regression test and Begg’s rank correlation test were used to assess publication bias. If publication bias was detected, the trim and fill method was used to evaluate funnel plot asymmetry (10). Meta-analysis was performed on influencing factors reported in more than two included studies. OR values and 95% CI were used as effect measures, with P< 0.05 considered statistically significant.

## 3 Results

### 3.1 Study selection

**Figure 1:**
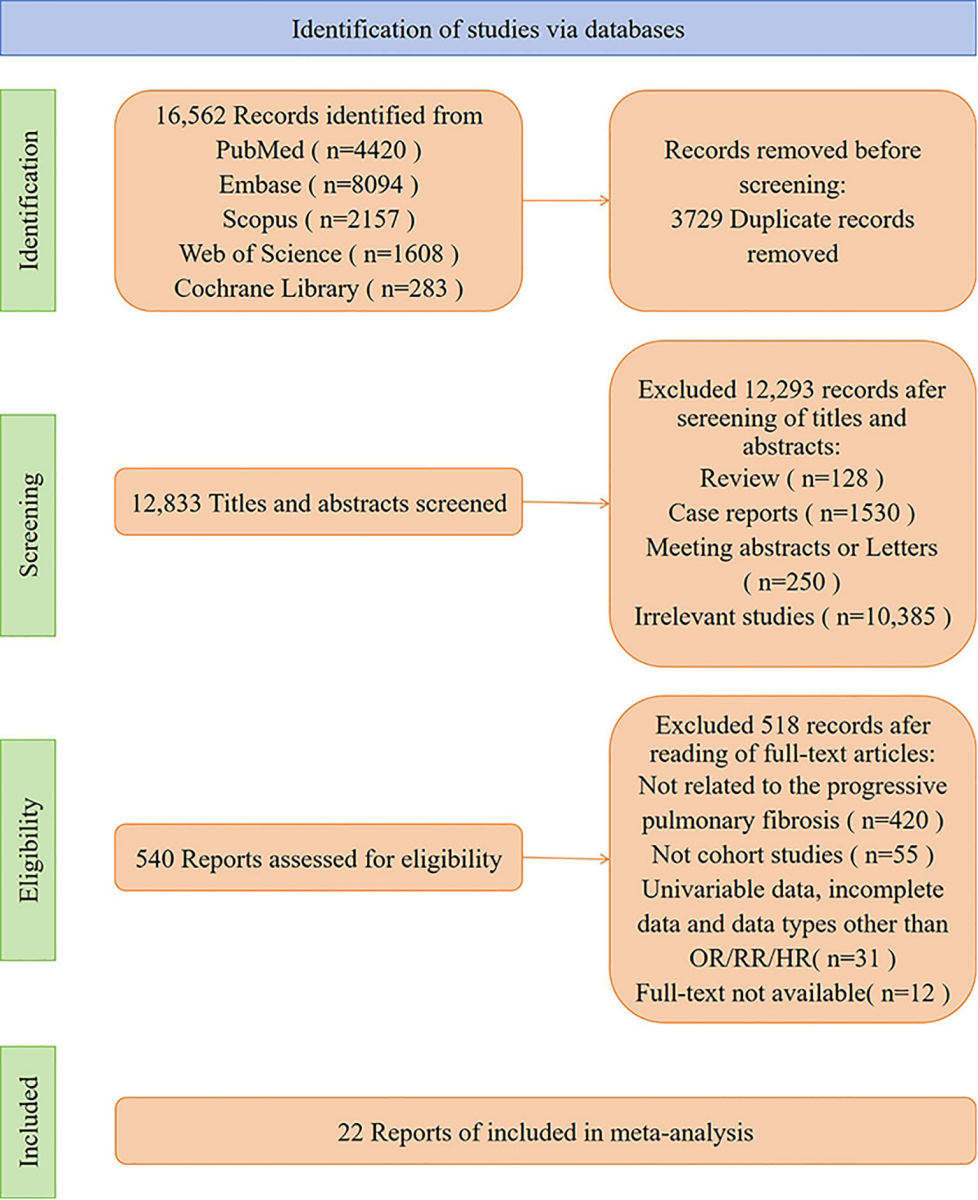
Flow diagram showing literature search and results.

A total of 16,562 articles were identified through searches. After removing 3,729 duplicate articles, 12,293 articles were excluded after reviewing titles and abstracts. Following full-text review, 518 articles were excluded, including 420 unrelated to PPF, 55 non-cohort studies, 31 excluded due to univariate data, incomplete data, or incompatible data types, and 12 articles for which full text was unavailable. An updated search in PubMed identified one additional article, resulting in the inclusion of 22 studies that met the criteria for systematic review and meta-analysis (11–32).The literature selection process is illustrated in **Figure 1**.

### 3.2 Study characteristics and quality assessment

This study ultimately included 22 articles, comprising 20 cohort studies and 2 case-control studies, encompassing a total of 3,224 CTD-ILD patients. Among them, 924 patients developed PPF, and a total of 31 influencing factors were investigated. The NOS assessment identified 20 high-quality articles and 2 medium-quality articles. The basic characteristics of the included studies and quality assessment results in **Supplementary Table 2**.

As summarized in **Supplementary Table 3**, most included studies were either fully or partially aligned with the 2022 PPF definition, although substantial variation existed in how the criteria were operationalized. All studies applied a 12-month observation window to define disease progression, but they differed in the specific combinations of PPF domains assessed. Physiological and radiological criteria were consistently applied across studies, whereas systematic assessment of symptom progression was less frequently reported.

### 3.3 Incidence of PPF in CTD-ILD

#### 3.3.1 Overall incidence

**Figure 2:**
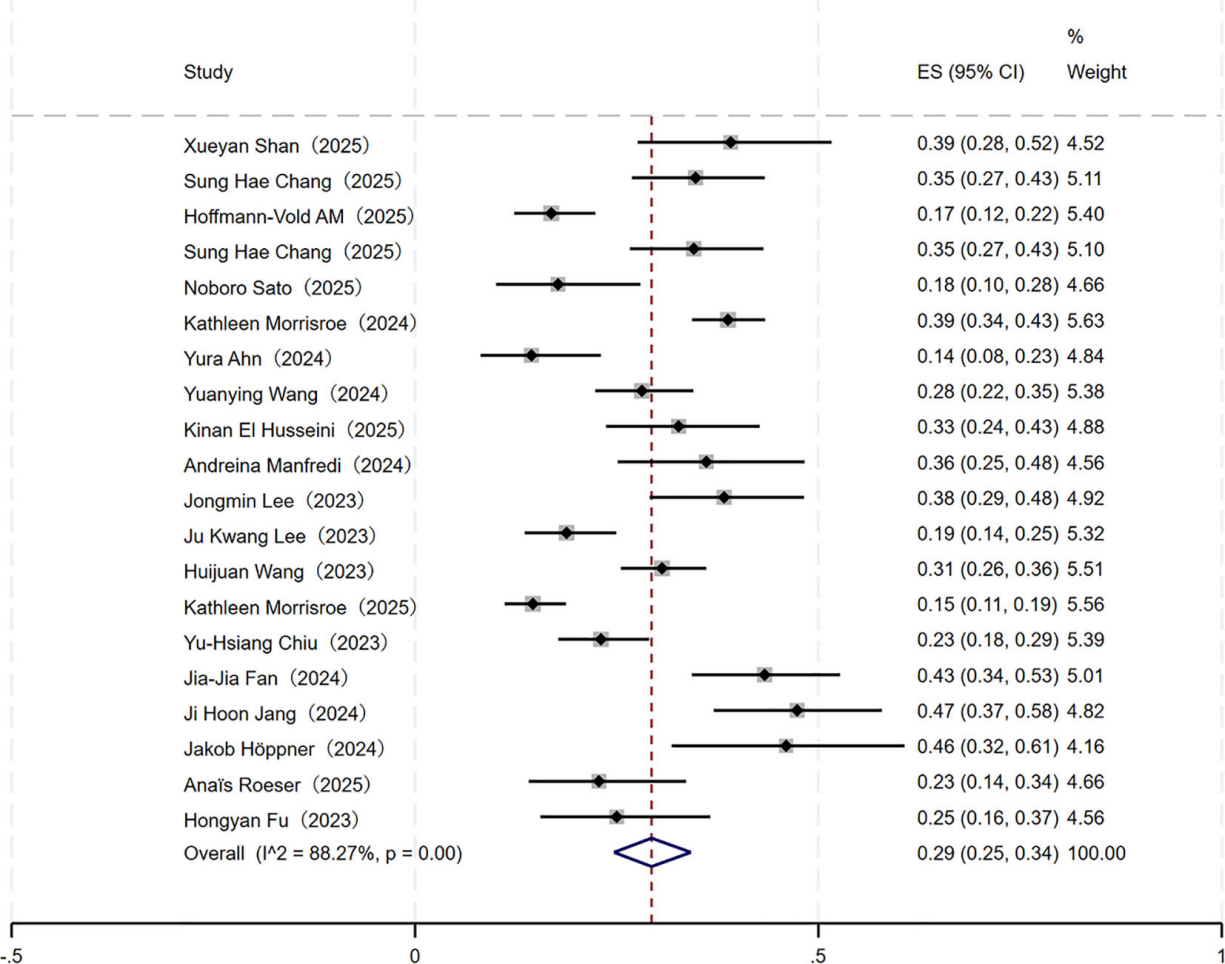
Forest plot of the overall incidence of PPF in patients with CTD-ILD.

A meta-analysis of 20 included cohort studies revealed significant heterogeneity among the studies (I² = 88.27%, P < 0.001) based on the heterogeneity test (**Table 1**; **Figure 2**). Consequently, a random-effects model was employed to pool the effect size. The results demonstrated a pooled incidence rate of PPF in CTD-ILD patients at 29% (95% CI: 25% - 34%).

**Table 1:** Meta-analysis results of PPF incidence in CTD-ILD patients.

Subgroups	Number of included studies (Articles)	Heterogeneity test		Effect Model	Bias assessment		Meta-analysis results		
		*I²*(%)	*P value*		Egger’s test	Begg’s test	Incidence rate (%)	95% *CI*(%)	*P value*
Overall Incidence	20	88.27	<0.001	Random	0.401	0.205	29	25~34	
CTD subtypes					-	-			0.316
RA	6	85.56	<0.001	Random	-	-	28	18~39	
SSc	7	92.47	<0.001	Random	-	-	19	11~30	
IIM	7	62.91	0.01	Random	-	-	31	24~38	
pSS	3	-	-	Random	-	-	32	23~42	
SLE	2	-	-	Random	-	-	18	6~34	
MCTD	1	-	-	Random	-	-	38	9~76	
Region					-	-			0.605
Asia	12	82.97	<0.001	Random	0.648	0.537	30	25~36	
Europe	6	81.46	<0.001	Random	-	-	28	21~36	
Oceania	2	-	-	Random	-	-	27	24~31	
Mean age					-	-			0.251
<60 years	5	89.70	<0.001	Random	-	-	24	16~33	
≥60 years	6	77.75	<0.001	Random	-	-	32	25~39	
Gender					-	-			0.163
Male	12	60.42	<0.001	Random	0.882	0.680	31	24~39	
Female	12	86.97	<0.001	Random	0.797	0.783	25	19~32	

“-” indicates that heterogeneity test (fewer than 3 included studies), Egger’s test, and Begg’s test (fewer than 10 included studies) were not performed.

#### 3.3.2 Subgroup analysis

Stratified subgroup analyses were conducted based on CTD subtypes, geographic region, mean age, and gender. Results showed: (1) CTD subgroups: The probability of developing PPF was 31% (95% CI: 24% - 38%) in IIM, 28% (95% CI: 18% - 39%) in RA, 32% (95% CI: 23% - 42%) in pSS, 19% (95% CI: 11% - 30%) in SSc, and 18% (95% CI: 6% - 34%) in SLE. For MCTD, which was represented in only one study, the incidence rate was 38% (95% CI: 9% - 76%) and only serves as a descriptive reference. (2) Regional subgroup analysis: The incidence of PPF was highest among Asian patients (30%, 95% CI: 25% - 36%), followed by European patients (28%, 95% CI: 21% - 36%), which was slightly higher than that in Oceania (27%, 95% CI: 24% - 31%). (3) Age subgroup analysis using 60 years as the threshold: Patients aged ≥60 years showed a higher incidence of PPF (31%, 95% CI: 25% - 39%) compared to those aged <60 years (24%, 95% CI: 16% - 33%). (4) Gender subgroup analysis: Male patients exhibited a higher incidence of PPF (32%, 95% CI: 24% - 39%) than female patients (25%, 95% CI: 19% - 32%). The results of intergroup difference tests showed that the differences in the incidence of PPF among the above subgroups were not statistically significant (P > 0.05), as shown in **Table 1**; **Supplementary Figures 1**-**4**.

#### 3.3.3 Meta-regression analysis

Meta-regression analyses were conducted using CTD subtypes, geographic region, age, and gender as covariates to explore potential sources of heterogeneity in the pooled incidence of PPF. The results showed that none of these covariates had a statistically significant impact on the effect size (P > 0.05), and no specific source of heterogeneity was identified. The adjusted coefficients of determination indicated that these variables explained only a limited proportion of the between-study variability, suggesting that residual heterogeneity may be attributable to other unmeasured study-level factors (**Table 2**; **Supplementary Figures 5**-**8**).

**Table 2:** Meta-regression analysis of PPF incidence in CTD-ILD patients.

Covariate	β (95%CI)	SE	t-value	P-value
CTD subtype category	0.0056 (-0.0487~0.0599)	0.0263	0.21	0.833
Mean age	0.0793 (-0.0479~0.2065)	0.0562	1.41	0.192
Region	-0.0219 (-0.0903~0.0464)	0.0325	-0.67	0.509
Gender	-0.0624 (-0.1796~0.0548)	0.0565	-1.10	0.281

#### 3.3.4 Sensitivity analysis

Sensitivity analysis was performed using the method of sequentially excluding individual studies. The results showed that after excluding any single study, the incidence of PPF in CTD-ILD patients ranged from 28% to 30%. All studies were evenly distributed on both sides of the vertical line, with no significant changes compared to the overall incidence rate, indicating robust stability of the study results (**Supplementary Figure 9**).

#### 3.3.5 Publication bias

**Figure 3:**
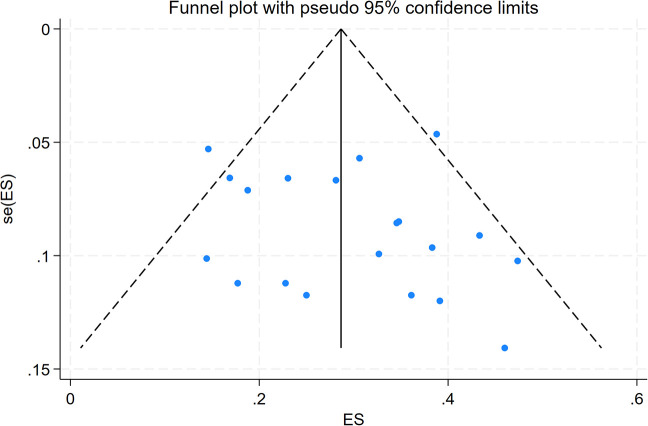
Funnel plot of the overall incidence of PPF in patients with CTD-ILD.

Publication bias was assessed by generating a funnel plot, which showed a roughly symmetrical distribution of study points, suggesting a low risk of publication bias (**Figure 3**). The results of Egger’s regression test (t = 0.86, P = 0.401) and Begg’s rank correlation test (z = 1.27, P = 0.205) indicated no significant publication bias in the overall analysis. Additionally, trim and fill method analysis revealed no substantial fluctuation in the pooled effect size before and after adjustment, further validating the reliability of the above conclusion (**Supplementary Figure 10**). For subgroups with more than 10 included studies, Egger’s and Begg’s tests yielded P > 0.05, indicating no independent publication bias risk within each subgroup.

#### 3.3.6 Localization of heterogeneity sources and extreme value validation

Further exploration of heterogeneity sources and the influence weight of individual studies through Galbraith plots revealed that all effect size data points were approximately distributed along the regression line, with no significantly deviated outlier data points. No studies exerting extreme influence on the pooled effect size were identified, thereby further validating the robustness of this study’s findings (**Supplementary Figure 11**).

### 3.4 Influencing factors associated with PPF in CTD-ILD

#### 3.4.1 Meta-analysis results

As shown in **Table 3**; **Supplementary Figures 12**-**18**, a total of 12 studies reported factors associated with the development of PPF in CTD-ILD. Combined analysis of identical factors reported in ≥ 2 studies demonstrated that elevated levels of KL-6, hSP-D, MMP-7, and CA-125 were identified as risk factors for the progression of CTD-ILD to PPF (P < 0.05). Baseline high FVC% predicted was a protective factor against progression to PPF in CTD-ILD (P < 0.05).

**Table 3:** Meta-analysis of influencing factors for progression to PPF in CTD-ILD.

Influencing factors	Number of included studies (Articles)	Heterogeneity test		Effect model	Meta-analysis		
		*I²*(%)	*P value*		OR	95% *CI*	*P value*
FVC% predicted	4	1.8	0.383	Fixed	0.98	0.96~0.99	0.001
hSP-D	2	0	0.837	Fixed	1.48	1.16~1.90	0.002
MMP-7	2	5.1	0.305	Fixed	1.48	1.13~1.93	0.004
CA-125	2	0	0.366	Fixed	1.19	1.05~1.34	0.006
KL-6	4	65.8	0.033	Random	2.21	1.24~3.94	0.007
DLCO	6	93.7	<0.001	Random	1.07	0.95~1.21	0.236
CEA	3	77.4	0.012	Random	1.83	0.77~4.34	0.171

#### 3.4.2 Descriptive analysis

For potential factors reported in only one included study, where meta-analysis pooling was not feasible, descriptive analysis was employed. Reported risk factors across included studies encompassed: elevated Lactate Dehydrogenase, elevated Cancer Antigen 50, elevated DAS28-58 (ESR), definite or possible usual interstitial pneumonia (UIP) pattern on imaging, ILD involvement > 10%, per unit increase in total fibrosis score, increased reticular opacity score, traction bronchiectasis, increased honeycombing score, elevated anti-CCP antibodies, advanced age, diffuse systemic sclerosis, positive anti-Scl-70 antibody, short telomere length, occurrence of acute or subacute ILD, diffuse alveolar damage on HRCT, pulmonary hypertension, and short-term progression of PPF. Relevant protective factors include: positive anti-centromere antibody, triple therapy with glucocorticoids, immunosuppressants, and antifibrotic drugs, parotid gland enlargement, arthritis, neutrophil-to-lymphocyte ratio, and positive anti-non-Jo-1 antibodies. Additionally, two studies each suggest that elevated carcinoembryonic antigen (CEA) and reduced Diffusing Capacity of the Lung for Carbon Monoxide (DLCO) may be risk factors for CTD-ILD progression to PPF, while another study indicates that elevated DLCO may serve as a protective factor.

#### 3.4.3 Sensitivity analysis and publication bias testing

Sensitivity analyses were conducted at both the statistical and definitional levels to evaluate the robustness of the pooled estimates. For factors examined in two or more studies, statistical sensitivity analyses were performed using both fixed-effect and random-effects models. In addition, leave-one-out analyses were conducted by sequentially removing each study to recalculate pooled OR values and 95% CI. Results showed that, except for CEA, the overall conclusions for the remaining factors remained stable regardless of model selection or exclusion of individual studies, indicating good statistical robustness of the meta-analytic findings (**Table 4**).

**Table 4:** Sensitivity analysis of influencing factors for progression to PPF in CTD-ILD.

Influencing factors	Fixed-effects model		Random-effects model	
	OR	95% *CI*	OR	95% *CI*
FVC% predicted	0.98	0.96~0.99	0.98	0.96~0.99
hSP-D	1.48	1.16~1.90	1.48	1.16~1.90
MMP-7	1.48	1.13~1.94	1.48	1.13~1.94
CA-125	1.19	1.05~1.34	1.19	1.05~1.34
KL-6	1.67	1.32~2.10	2.21	1.24~3.94
DLCO	1.01	0.99~1.02	1.07	0.95~1.27
CEA	1.47	1.05~2.05	1.82	0.77~4.34

At the definitional level, sensitivity analyses excluding studies that substantially deviated from the core construct of PPF were considered. However, no included study was identified as fundamentally inconsistent with the 2022 PPF definition. Therefore, a formal exclusion-based sensitivity analysis was not performed. Instead, transparency regarding variability in operational definitions was ensured through systematic documentation of observation windows, assessed domains, and progression thresholds across studies.

Owing to the limited number of studies included in the pooled analyses, formal assessments of publication bias using Egger’s or Begg’s tests were not performed because of their low statistical power, which may introduce a potential risk of publication bias.

## 4 Discussion

This systematic review and meta-analysis explored the incidence and influencing factors of PPF in patients with CTD-ILD. First, this study consolidated existing data and identified a PPF incidence rate of 29% in CTD-ILD patients. Second, strong evidence indicates that elevated levels of KL-6, hSP-D, MMP-7, and CA-125 are risk factors for PPF development. Finally, a higher baseline FVC% predicted was identified as a protective factor against PPF development in CTD-ILD.

### 4.1 Incidence of PPF in CTD-ILD

This study included 3,224 CTD-ILD patients from 20 cohort studies, confirming a PPF incidence rate of 29%. Although comparable to prior PF-ILD– or RP-ILD–based estimates (approximately 18–40%), the use of the 2022 guideline-defined, time-bounded PPF criteria provides greater specificity and clinical applicability for early risk stratification (33). To explore the sources of heterogeneity, stratified analyses were performed based on CTD subtypes, geographic region, age, and gender. The results revealed no statistically significant differences across subgroups, indicating that these factors are not primary sources of heterogeneity. Sensitivity analysis demonstrated that the incidence rate ranged from 28% to 30%. Combined with validation through the trim and fill method showing no substantial fluctuation in the pooled effect size, these findings indicate robust stability of the overall incidence rate. Residual heterogeneity may be associated with potential confounding factors such as sample size, duration of study follow-up, and diagnostic criteria.

The study revealed variations in PPF incidence across different CTD subtypes, with distinct underlying mechanisms exhibiting marked specificity. This pattern was particularly pronounced in IIM-ILD, SSc-ILD, and RA-ILD. IIM-ILD exhibits multiple specific autoantibodies, which not only serve as diagnostic markers but also act as key factors contributing to PPF. Studies indicate that autoantibodies such as anti-MDA5 and anti-Ro52 tend to trigger intense pulmonary inflammatory responses, leading to rapid deterioration in lung imaging and pulmonary function within a short period. This subsequently drives persistent fibrosis in lung tissue, significantly increasing the risk of PPF occurrence (34–36). The development of PPF in SSc-ILD patients is closely associated with autoantibodies, immune/inflammatory factors, and vascular endothelial injury (37). Anti-topoisomerase I antibody is a significant factor in the development of PPF, with clinical studies confirming its strong correlation with sustained decline in FVC (38, 39). Persistent activation of innate and adaptive immunity in SSc-ILD promotes overexpression of matrix metalloproteinase-9 through profibrotic signals such as transforming growth factor-β and interleukin-4, while macrophages release oxygen free radicals and inflammatory factors that drive fibrosis progression (40). Early endothelial injury further increases vascular permeability and releases platelet-derived growth factor, which continuously stimulates fibroblasts and drives lung tissue into an irreversible progressive fibrotic state (41). The incidence of PPF in RA-ILD is relatively high, which is consistent with findings reported by Olson A et al (33). This may be attributable to the higher proportion of UIP imaging patterns in RA-ILD, which itself serves as a strong predictor for fibrosis progression (42, 43). Simultaneously, RA patients often exhibit high titers of anti-citrullinated protein antibodies and positive rheumatoid factor. These autoantibodies not only closely correlate with the occurrence of ILD but also act as significant drivers for the sustained progression of PPF (44). It should be noted that subgroup analyses for pSS-ILD, SLE-ILD, and MCTD-ILD in this study were based on limited sample sizes (≤3 studies), failing to meet optimal sample size requirements for heterogeneity testing and pooling of effect sizes. Consequently, the reported incidence rates lack representativeness and may inadequately reflect real-world clinical scenarios. Conclusions warrant further validation through multicenter, large-sample cohort studies to clarify their true progression risks.

Regional analysis revealed no significant difference in the incidence of PPF among CTD-ILD patients across Asia, Europe, and Oceania, suggesting that the development of PPF in CTD-ILD patients is more likely related to the pathological mechanisms of the disease itself (e.g., types of autoantibodies) rather than geographical environmental differences. The study found that elderly CTD-ILD patients are more susceptible to PPF. This is attributed to the decline in lung tissue repair capacity, telomere shortening, and cellular senescence in the elderly, which predispose them to abnormal fibroblastic responses following recurrent injuries. This constitutes a key mechanism driving fibrosis progression (45). Elderly patients often have a history of smoking, cardiopulmonary comorbidities, and exposure to multiple medications, with longer exposure duration. These factors reduce functional reserve and promote disease progression (46). Clinical studies indicate that advanced age is an independent risk factor for CTD-ILD deterioration and mortality (47). Concurrently, delayed diagnosis and intervention due to non-specific symptoms or comorbidities in elderly patients increase the risk of disease progression. Elderly individuals often present with baseline pulmonary function decline and radiological evidence of fibrosis progression. These adverse baseline characteristics accelerate fibrosis progression, resulting in a higher incidence of PPF in this population. Regarding gender, males demonstrated a higher risk of developing PPF compared to females. Multiple studies indicate that male gender constitutes one of the risk factors for disease progression in CTD-ILD (48, 49). An animal study suggests that androgens can exacerbate bleomycin-induced pulmonary fibrosis, leading to increased fibroblast activation and greater deposition of extracellular matrix. Conversely, several *in vitro* and *in vivo* studies suggest that estrogen may inhibit certain profibrotic responses in fibroblasts, potentially explaining the male predisposition toward developing irreversible fibrosis (50). Telomere shortening and telomere-related gene variations have been demonstrated to be associated with susceptibility to fibrosis and rapid progression (51). Studies suggest a link between sex hormones and telomere dynamics, with androgen levels potentially influencing leukocyte telomere length; in cohort studies, patients with shorter telomeres are more prone to rapid progression of pulmonary fibrosis (52, 53). In most populations, males exhibit higher rates of smoking history and occupational exposure history. These factors promote lung epithelial damage, chronic inflammation, and fibrosis-driving pathways, leading to the progression of pulmonary fibrosis (54).

### 4.2 Influencing factors for PPF occurrence in CTD-ILD

This study identified multidimensional influencing factors for PPF development in CTD-ILD by integrating data from multiple clinical studies, providing high-level evidence-based evidence for clinical risk stratification, early intervention, and prognosis assessment. The study confirmed that KL-6, hSP-D, MMP-7, and CA-125 are risk factors for CTD-ILD progression to PPF; A higher baseline FVC% predicted served as a protective factor against progression to PPF in CTD-ILD patients (P < 0.05). KL-6, a biomarker of pulmonary epithelial cell injury, directly binds to cluster of differentiation 44 receptors on fibroblast surfaces. This interaction activates the PI3K/Akt signaling pathway, promoting fibroblast proliferation and type I collagen synthesis, thereby accelerating matrix deposition (55). Multiple studies have indicated that CTD-ILD patients with elevated baseline KL-6 levels are more prone to pulmonary function decline, radiographic progression, and higher mortality (56, 57). Longitudinal studies suggest that a dynamic upward trend in KL-6 levels often coincides with pulmonary function decline, HRCT progression, or clinical deterioration. Serial measurement of KL-6 can be utilized to monitor disease severity and facilitate early identification of progression (58). hSP-D is a secretory protein synthesized by alveolar type II epithelial cells and Clara cells. Multiple studies indicate that parenchymal lung damage induces massive release of hSP-D, and its serum levels directly correlate with the extent of interstitial lung injury (59, 60). When alveolar epithelium is subjected to chronic inflammation, repetitive injury, or immune attack, disruption of the alveolar-capillary barrier causes hSP-D to leak into peripheral blood. Elevated serum hSP-D levels thus serve as a sensitive indicator of epithelial damage or active disease (61). In CTD-ILD, immune-mediated persistent epithelial damage is a key driver of fibrosis progression. Therefore, elevated serum hSP-D levels reflect an active pathological process, indicating a higher risk of disease progression (62). MMP-7 is primarily expressed by damaged epithelial cells and certain immune cells. When alveolar epithelium is injured or activated, MMP-7 expression is upregulated and released into alveolar fluid and bloodstream. Consequently, elevated serum levels are considered a marker of epithelial injury and active remodeling (63). MMP-7 induces epithelial-mesenchymal transition, disrupting the epithelial barrier and facilitating the transformation of epithelial cells into mesenchymal cells (64). Additionally, elevated MMP-7 can directly or indirectly activate proliferation and differentiation signaling in fibroblasts, exacerbating the synthesis and deposition of extracellular matrix. Multiple studies indicate that elevated MMP-7 can cleave multiple matrix proteins and extracellular matrix-related factors, altering the matrix microenvironment. This facilitates fibroblast migration, proliferation, and matrix deposition, thereby promoting fibrotic progression (65–67). Cohort studies demonstrate that serum MMP-7 concentration in CTD-ILD patients is significantly positively correlated with the risk of ILD progression (68, 69). Statistical analysis results showed that CA-125 is also a risk factor contributing to PPF. CA-125 is primarily secreted by epithelial or mesothelial cells, and its levels are often elevated in CTD-ILD patients, reflecting active inflammation and epithelial damage. Clinical studies have found a significant positive correlation between serum CA-125 levels and pulmonary fibrosis progression, with some research suggesting that CA-125 has predictive value for identifying progressive ILD (19, 70). The advantage of CA-125 lies in its simplicity of detection and low cost, but its specificity is insufficient, often requiring comprehensive interpretation in conjunction with imaging, pulmonary function tests, and other biomarkers. Interestingly, our study showed that a higher baseline FVC% predicted is a protective factor against progression to PPF in CTD-ILD. This is because the indicator reflects pulmonary structural integrity, functional reserve, and disease control status. The American College of Rheumatology indicates that changes in FVC levels at 12 or 24 weeks hold significant prognostic value for disease progression at 52 weeks in patients with CTD-ILD. Patients with increased or minimally decreased FVC demonstrate a significantly reduced risk of ILD progression. Adequate pulmonary functional reserve can buffer against sustained autoimmune inflammatory damage to the lungs, preventing rapid pulmonary function deterioration into PPF (71). Beyond their biological plausibility, KL-6, hSP-D, and MMP-7 have potential value for clinical risk stratification in CTD-ILD and may help identify patients at increased risk of developing PPF at an earlier, potentially modifiable stage. When integrated with baseline lung function and imaging features, these biomarkers may facilitate closer monitoring and earlier, individualized therapeutic decision-making, highlighting the potential of biomarker-guided strategies for early screening and management of PPF in CTD-ILD.

Interpretation of these influencing factors should be approached with appropriate caution. Although KL-6, hSP-D, MMP-7, CA-125, and a higher baseline FVC% predicted were identified as factors associated with PPF in CTD-ILD, evidence for subtype-specific prediction remains limited, as most studies involved mixed CTD-ILD populations or were dominated by particular subtypes such as RA-ILD or SSc-ILD. Accordingly, while the present findings support an overall association between these influencing factors and PPF, they do not yet allow definitive conclusions regarding uniform applicability across all CTD subtypes. Subtype-related differences in immunopathology and fibrotic mechanisms may further influence these associations, underscoring the need for future large-scale, subtype-stratified prospective studies to refine and validate their clinical utility.

### 4.3 Strengths and limitations

Patients with CTD-ILD who develop PPF experience a markedly shortened median survival, highlighting the urgent clinical need to clarify the incidence of PPF and its influencing factors to facilitate early identification and timely intervention. By applying the latest international consensus definition of PPF, this study represents the first systematic integration of available evidence to quantify the incidence of PPF in CTD-ILD and to summarize factors associated with its development. Compared with previous studies based on broader constructs such as PF-ILD or RP-ILD, our findings offer greater specificity and potential clinical applicability. Furthermore, subgroup analyses by CTD subtypes, geographic region, age, and gender provide a more granular basis for risk stratification across diverse populations. Several limitations should nevertheless be acknowledged. Substantial heterogeneity in reported PPF incidence was observed across studies, likely reflecting variability in observation windows, diagnostic thresholds, and combinations of physiological, radiological, and symptomatic criteria, as also noted in the 2023 expert consensus statement (72). To enhance transparency and address this issue, we systematically summarized the operational definitions of PPF used across all included studies. Importantly, although operational details varied, no study employed definitions that fundamentally deviated from the core construct of progressive pulmonary fibrosis, supporting the overall robustness and clinical relevance of our findings. For certain rare biomarkers or imaging features, the limited number of available studies and small sample sizes precluded quantitative synthesis, potentially leading to underestimation of relevant influencing factors. Moreover, although most included studies were rated as high quality based on the NOS, the absence of domain-specific tools for prognostic research (e.g., QUIPS) may have resulted in residual bias, particularly related to time-varying confounders such as changes in treatment regimens. Accordingly, the identified factors should be interpreted as associations rather than causal determinants. Finally, as the definition and assessment of PPF continue to evolve, current unified criteria may not be uniformly applicable across all ILD subtypes. Notably, PPF patients with CTD-ILD appear to have more favorable survival than those with other forms of ILD, suggesting potential bias when applying a single PPF framework to a heterogeneous CTD-ILD population (73). Future large-scale, multicenter prospective cohort studies using optimized, CTD-ILD–specific PPF criteria and domain-based bias assessment frameworks are therefore warranted to further validate the identified factors and refine incidence estimates.

## 5 Conclusions

Current evidence indicates that CTD-ILD patients are at high risk for developing PPF, and this progression risk is associated with elevated levels of KL-6, hSP-D, MMP-7, and CA-125. These findings provide a quantitative basis for assessing PPF risk in CTD-ILD patients and confirm that PPF represents an adverse phenotype requiring focused attention in the clinical management of CTD-ILD patients. This study helps researchers, clinicians, and patients better understand the risk of CTD-ILD progressing to PPF and its influencing factors, providing a basis for developing preventive strategies to improve prognosis and prolong survival.

## Data Availability

The original contributions presented in the study are included in the article/**Supplementary Material**. Further inquiries can be directed to the corresponding author.
