# Supplementary material for: Incidence and influencing factors of progressive pulmonary fibrosis in connective tissue disease-associated interstitial lung disease: a systematic review and meta-analysis
Source: Front Immunol. 2026 Feb 4;17:1758437. doi: 10.3389/fimmu.2026.1758437 (PMC12913582; doi:10.3389/fimmu.2026.1758437)
Supplement: Supplementary file 1 [file Table1.docx]

Supplementary Material

**Table of Contents for Supplementary Material**

[1 Supplementary Table 1: Search Strategy 2](#_Toc219028160)

[2 Supplementary Table 2: Basic Characteristics and Quality Assessment Results of Included Studies 17](#_Toc219028161)

[3 Supplementary Table 3: Assessment of consistency with the PPF definition 19](#_Toc219028162)

[4 Meta-Analysis Results of PPF Incidence in CTD-ILD 22](#_Toc219028163)

[4.1 Subgroup Analysis 22](#_Toc219028164)

[4.1.1 Specific Types of CTD 22](#_Toc219028165)

[4.1.2 Mean age 22](#_Toc219028166)

[4.1.3 Region 23](#_Toc219028167)

[4.1.4 Gender 24](#_Toc219028168)

[4.2 Meta-regression analysis 25](#_Toc219028169)

[4.2.1 Specific Types of CTD 25](#_Toc219028170)

[4.2.2 Mean age 26](#_Toc219028171)

[4.2.3 Region 27](#_Toc219028172)

[4.2.4 Gender 27](#_Toc219028173)

[4.3 Sensitivity analysis 28](#_Toc219028174)

[4.4 Bias Assessment 29](#_Toc219028175)

[4.5 Localization of Heterogeneity Sources and Extreme Value Verification 30](#_Toc219028176)

[5 Meta-Analysis Results of Factors Influencing PPF Occurrence in Patients with CTD-ILD 31](#_Toc219028177)

[5.1 Forest Plot of CA-125 31](#_Toc219028178)

[5.2 Forest Plot of hSP-D 32](#_Toc219028179)

[5.3 Forest Plot of KL-6 32](#_Toc219028180)

[5.4 Forest Plot of MMP-7 32](#_Toc219028181)

[5.5 Forest Plot of FVC% predicted 33](#_Toc219028182)

[5.6 Forest Plot of CEA 33](#_Toc219028183)

[5.7 Forest Plot of DLCO 34](#_Toc219028184)

# Supplementary Table 1: Search Strategy

Search strategies for the different databases ran on **August 25, 2025**. This strategy is adapted to identify trials in other electronic databases

PubMed (4420)

| Search number | Query | Search Details | Results |
| --- | --- | --- | --- |
| 31 | ((((((((((((((((((("Arthritis, Rheumatoid"[Mesh]) OR (((rheumatoid arthritis) OR (Rheumatoid Arthritis)) OR (Arthritis, Rheumatoid))) OR ("Scleroderma, Systemic"[Mesh])) OR (((((scleroderma) OR (Sclerosis, Systemic)) OR (Systemic Scleroderma)) OR (Systemic Sclerosis)) OR (Scleroderma, Systemic))) OR ("Myositis"[Mesh])) OR ((((((((((((((((((((((((((((((antisynthetase syndrome) OR (myositis)) OR (Myositides)) OR (Inflammatory Muscle Diseases)) OR (Inflammatory Muscle Disease)) OR (Muscle Disease, Inflammatory)) OR (Inflammatory Myopathy)) OR (Inflammatory Myopathies)) OR (Myopathies, Inflammatory)) OR (Muscle Diseases, Inflammatory)) OR (Myopathy, Inflammatory)) OR (Myositis, Focal)) OR (Focal Myositides)) OR (Focal Myositis)) OR (Myositides, Focal)) OR (Myositis, Proliferative)) OR (Myositides, Proliferative)) OR (Proliferative Myositides)) OR (Proliferative Myositis)) OR (Myositis, Infectious)) OR (Infectious Myositides)) OR (Myositides, Infectious)) OR (Infectious Myositis)) OR (Idiopathic Inflammatory Myopathies)) OR (Myopathy, Idiopathic Inflammatory)) OR (Inflammatory Myopathies, Idiopathic)) OR (Myopathies, Idiopathic Inflammatory)) OR (Idiopathic Inflammatory Myopathy)) OR (Idiopathic Inflammatory Myositis)) OR (Inflammatory Myopathy, Idiopathic))) OR ("Polymyositis"[Mesh])) OR (((((((((((polymyositis) OR (Polymyositides)) OR (Myositis, Multiple)) OR (Multiple Myositis)) OR (Myositides, Multiple)) OR (Polymyositis Ossificans)) OR (Ossificans, Polymyositis)) OR (Polymyositis, Idiopathic)) OR (Idiopathic Polymyositides)) OR (Idiopathic Polymyositis)) OR (Polymyositides, Idiopathic))) OR ("Dermatomyositis"[Mesh])) OR ((((((((((((dermatomyositis) OR (Polymyositis-Dermatomyositis)) OR (Polymyositis Dermatomyositis)) OR (Dermatopolymyositis)) OR (Dermatomyositis, Adult Type)) OR (Adult Type Dermatomyositis)) OR (Dermatomyositis, Childhood Type)) OR (Childhood Type Dermatomyositis)) OR (Juvenile Dermatomyositis)) OR (Dermatomyositis, Juvenile)) OR (Juvenile Myositis)) OR (Myositis, Juvenile))) OR ("Sjogren's Syndrome"[Mesh])) OR (((((((sjogren syndrome) OR (Sjögren syndrome)) OR (Sjogrens Syndrome)) OR (Syndrome, Sjogren's)) OR (Sjogren Syndrome)) OR (Sicca Syndrome)) OR (Syndrome, Sicca))) OR ("Mixed Connective Tissue Disease"[Mesh])) OR (((((mixed connective tissue disease) OR (MCTD)) OR (Connective Tissue Disease, Mixed)) OR (Sharp Syndrome)) OR (Syndrome, Sharp))) OR ("Lupus Erythematosus, Systemic"[Mesh])) OR ((((((systemic lupus erythematosus) OR (Lupus Erythematosus Disseminatus)) OR (Systemic Lupus Erythematosus)) OR (Libman-Sacks Disease)) OR (Disease, Libman-Sacks)) OR (Libman Sacks Disease))) OR ("Connective Tissue Diseases"[Mesh])) OR ((((connective tissue disease) OR (Connective Tissue Disease)) OR (Disease, Connective Tissue)) OR (Diseases, Connective Tissue))) AND (("Lung Diseases, Interstitial"[Mesh]) OR ((((((((((((((interstitial lung disease) OR (Diffuse Parenchymal Lung Diseases)) OR (Interstitial Lung Diseases)) OR (Interstitial Lung Disease)) OR (Lung Disease, Interstitial)) OR (Diffuse Parenchymal Lung Disease)) OR (Pneumonia, Interstitial)) OR (Interstitial Pneumonia)) OR (Interstitial Pneumonias)) OR (Pneumonias, Interstitial)) OR (Pneumonitis, Interstitial)) OR (Interstitial Pneumonitides)) OR (Interstitial Pneumonitis)) OR (Pneumonitides, Interstitial)))) AND ((((((("Symptom Flare Up"[Mesh]) OR (((((((((((((((((((((((((((((((exacerbation) OR (Symptom Flare Up)) OR (Flare Ups, Symptom)) OR (Flare Up, Symptom)) OR (Symptom Flare Ups)) OR (Symptom Flareup)) OR (Flareups, Symptom)) OR (Flareup, Symptom)) OR (Symptom Flareups)) OR (Symptom Flare-up)) OR (Flare-ups, Symptom)) OR (Flare-up, Symptom)) OR (Symptom Flare-ups)) OR (Acute Symptom Flare)) OR (Acute Symptom Flares)) OR (Symptom Flare, Acute)) OR (Symptom Flaring Up)) OR (Flaring Up, Symptom)) OR (Symptom Exacerbation)) OR (Exacerbation, Symptom)) OR (Symptom Exacerbations)) OR (Symptom Worsening)) OR (Worsening, Symptom)) OR (Symptom Exaggeration)) OR (Exaggeration, Symptom)) OR (Symptom Exaggerations)) OR (Symptom Magnification)) OR (Magnification, Symptom)) OR (Symptom Magnifications)) OR (Symptom Increase)) OR (Increase, Symptom))) OR ("Disease Progression"[Mesh])) OR (((((((Disease Progression) OR (Progression, Disease)) OR (Disease Exacerbation)) OR (Exacerbation, Disease)) OR (Clinical Course)) OR (Clinical Progression)) OR (Progression, Clinical))) OR ("Clinical Deterioration"[Mesh])) OR ((((deterioration) OR (Clinical Deterioration)) OR (Clinical Deteriorations)) OR (Deterioration, Clinical))) OR ((((((((Rapid Progress) OR (Acute Exacerbation)) OR (Clinical Deterioration)) OR (Exacerbation)) OR (Course)) OR (Progression)) OR (Progress)) OR (Progressive))) | ("arthritis, rheumatoid"[MeSH Terms] OR ("arthritis, rheumatoid"[MeSH Terms] OR ("arthritis"[All Fields] AND "rheumatoid"[All Fields]) OR "rheumatoid arthritis"[All Fields] OR ("rheumatoid"[All Fields] AND "arthritis"[All Fields]) OR ("arthritis, rheumatoid"[MeSH Terms] OR ("arthritis"[All Fields] AND "rheumatoid"[All Fields]) OR "rheumatoid arthritis"[All Fields] OR ("rheumatoid"[All Fields] AND "arthritis"[All Fields])) OR ("arthritis, rheumatoid"[MeSH Terms] OR ("arthritis"[All Fields] AND "rheumatoid"[All Fields]) OR "rheumatoid arthritis"[All Fields] OR "arthritis rheumatoid"[All Fields])) OR "scleroderma, systemic"[MeSH Terms] OR ("scleroderma, systemic"[MeSH Terms] OR ("scleroderma"[All Fields] AND "systemic"[All Fields]) OR "systemic scleroderma"[All Fields] OR "scleroderma"[All Fields] OR "scleroderma, localized"[MeSH Terms] OR ("scleroderma"[All Fields] AND "localized"[All Fields]) OR "localized scleroderma"[All Fields] OR "sclerodermas"[All Fields] OR ("scleroderma, systemic"[MeSH Terms] OR ("scleroderma"[All Fields] AND "systemic"[All Fields]) OR "systemic scleroderma"[All Fields] OR ("sclerosis"[All Fields] AND "systemic"[All Fields]) OR "sclerosis systemic"[All Fields]) OR ("scleroderma, systemic"[MeSH Terms] OR ("scleroderma"[All Fields] AND "systemic"[All Fields]) OR "systemic scleroderma"[All Fields] OR ("systemic"[All Fields] AND "scleroderma"[All Fields])) OR ("scleroderma, systemic"[MeSH Terms] OR ("scleroderma"[All Fields] AND "systemic"[All Fields]) OR "systemic scleroderma"[All Fields] OR ("systemic"[All Fields] AND "sclerosis"[All Fields]) OR "systemic sclerosis"[All Fields]) OR ("scleroderma, systemic"[MeSH Terms] OR ("scleroderma"[All Fields] AND "systemic"[All Fields]) OR "systemic scleroderma"[All Fields] OR "scleroderma systemic"[All Fields])) OR "Myositis"[MeSH Terms] OR ("antisynthetase syndrome"[Supplementary Concept] OR "antisynthetase syndrome"[All Fields] OR ("Myositis"[MeSH Terms] OR "Myositis"[All Fields] OR "myositides"[All Fields]) OR ("Myositis"[MeSH Terms] OR "Myositis"[All Fields] OR "myositides"[All Fields]) OR ("Myositis"[MeSH Terms] OR "Myositis"[All Fields] OR ("inflammatory"[All Fields] AND "muscle"[All Fields] AND "diseases"[All Fields]) OR "inflammatory muscle diseases"[All Fields]) OR ("Myositis"[MeSH Terms] OR "Myositis"[All Fields] OR ("inflammatory"[All Fields] AND "muscle"[All Fields] AND "disease"[All Fields]) OR "inflammatory muscle disease"[All Fields]) OR ("Myositis"[MeSH Terms] OR "Myositis"[All Fields] OR ("muscle"[All Fields] AND "disease"[All Fields] AND "inflammatory"[All Fields])) OR ("Myositis"[MeSH Terms] OR "Myositis"[All Fields] OR ("inflammatory"[All Fields] AND "myopathy"[All Fields]) OR "inflammatory myopathy"[All Fields]) OR ("Myositis"[MeSH Terms] OR "Myositis"[All Fields] OR ("inflammatory"[All Fields] AND "myopathies"[All Fields]) OR "inflammatory myopathies"[All Fields]) OR ("Myositis"[MeSH Terms] OR "Myositis"[All Fields] OR ("myopathies"[All Fields] AND "inflammatory"[All Fields]) OR "myopathies inflammatory"[All Fields]) OR ("Myositis"[MeSH Terms] OR "Myositis"[All Fields] OR ("muscle"[All Fields] AND "diseases"[All Fields] AND "inflammatory"[All Fields]) OR "muscle diseases inflammatory"[All Fields]) OR ("Myositis"[MeSH Terms] OR "Myositis"[All Fields] OR ("myopathy"[All Fields] AND "inflammatory"[All Fields]) OR "myopathy inflammatory"[All Fields]) OR ("Myositis"[MeSH Terms] OR "Myositis"[All Fields] OR ("Myositis"[All Fields] AND "focal"[All Fields]) OR "myositis focal"[All Fields]) OR ("Myositis"[MeSH Terms] OR "Myositis"[All Fields] OR ("focal"[All Fields] AND "myositides"[All Fields])) OR ("Myositis"[MeSH Terms] OR "Myositis"[All Fields] OR ("focal"[All Fields] AND "Myositis"[All Fields]) OR "focal myositis"[All Fields]) OR ("Myositis"[MeSH Terms] OR "Myositis"[All Fields] OR ("myositides"[All Fields] AND "focal"[All Fields])) OR ("Myositis"[MeSH Terms] OR "Myositis"[All Fields] OR ("Myositis"[All Fields] AND "proliferative"[All Fields])) OR ("Myositis"[MeSH Terms] OR "Myositis"[All Fields] OR ("myositides"[All Fields] AND "proliferative"[All Fields])) OR ("Myositis"[MeSH Terms] OR "Myositis"[All Fields] OR ("proliferative"[All Fields] AND "myositides"[All Fields])) OR ("Myositis"[MeSH Terms] OR "Myositis"[All Fields] OR ("proliferative"[All Fields] AND "Myositis"[All Fields]) OR "proliferative myositis"[All Fields]) OR ("Myositis"[MeSH Terms] OR "Myositis"[All Fields] OR ("Myositis"[All Fields] AND "infectious"[All Fields]) OR "myositis infectious"[All Fields]) OR ("Myositis"[MeSH Terms] OR "Myositis"[All Fields] OR ("infectious"[All Fields] AND "myositides"[All Fields]) OR "infectious myositides"[All Fields]) OR ("Myositis"[MeSH Terms] OR "Myositis"[All Fields] OR ("myositides"[All Fields] AND "infectious"[All Fields])) OR ("Myositis"[MeSH Terms] OR "Myositis"[All Fields] OR ("infectious"[All Fields] AND "Myositis"[All Fields]) OR "infectious myositis"[All Fields]) OR ("Myositis"[MeSH Terms] OR "Myositis"[All Fields] OR ("idiopathic"[All Fields] AND "inflammatory"[All Fields] AND "myopathies"[All Fields]) OR "idiopathic inflammatory myopathies"[All Fields]) OR ("Myositis"[MeSH Terms] OR "Myositis"[All Fields] OR ("myopathy"[All Fields] AND "idiopathic"[All Fields] AND "inflammatory"[All Fields]) OR "myopathy idiopathic inflammatory"[All Fields]) OR ("Myositis"[MeSH Terms] OR "Myositis"[All Fields] OR ("inflammatory"[All Fields] AND "myopathies"[All Fields] AND "idiopathic"[All Fields])) OR ("Myositis"[MeSH Terms] OR "Myositis"[All Fields] OR ("myopathies"[All Fields] AND "idiopathic"[All Fields] AND "inflammatory"[All Fields]) OR "myopathies idiopathic inflammatory"[All Fields]) OR ("Myositis"[MeSH Terms] OR "Myositis"[All Fields] OR ("idiopathic"[All Fields] AND "inflammatory"[All Fields] AND "myopathy"[All Fields]) OR "idiopathic inflammatory myopathy"[All Fields]) OR ("Myositis"[MeSH Terms] OR "Myositis"[All Fields] OR ("idiopathic"[All Fields] AND "inflammatory"[All Fields] AND "Myositis"[All Fields]) OR "idiopathic inflammatory myositis"[All Fields]) OR ("Myositis"[MeSH Terms] OR "Myositis"[All Fields] OR ("inflammatory"[All Fields] AND "myopathy"[All Fields] AND "idiopathic"[All Fields]))) OR "Polymyositis"[MeSH Terms] OR ("Polymyositis"[MeSH Terms] OR "Polymyositis"[All Fields] OR ("Polymyositis"[MeSH Terms] OR "Polymyositis"[All Fields] OR "polymyositides"[All Fields]) OR ("Polymyositis"[MeSH Terms] OR "Polymyositis"[All Fields] OR ("Myositis"[All Fields] AND "multiple"[All Fields]) OR "myositis multiple"[All Fields]) OR ("Polymyositis"[MeSH Terms] OR "Polymyositis"[All Fields] OR ("multiple"[All Fields] AND "Myositis"[All Fields]) OR "multiple myositis"[All Fields]) OR ("Polymyositis"[MeSH Terms] OR "Polymyositis"[All Fields] OR ("myositides"[All Fields] AND "multiple"[All Fields])) OR ("Polymyositis"[MeSH Terms] OR "Polymyositis"[All Fields] OR ("Polymyositis"[All Fields] AND "ossificans"[All Fields]) OR "polymyositis ossificans"[All Fields]) OR ("Polymyositis"[MeSH Terms] OR "Polymyositis"[All Fields] OR ("ossificans"[All Fields] AND "Polymyositis"[All Fields]) OR "ossificans polymyositis"[All Fields]) OR ("Polymyositis"[MeSH Terms] OR "Polymyositis"[All Fields] OR ("Polymyositis"[All Fields] AND "idiopathic"[All Fields]) OR "polymyositis idiopathic"[All Fields]) OR ("Polymyositis"[MeSH Terms] OR "Polymyositis"[All Fields] OR ("idiopathic"[All Fields] AND "polymyositides"[All Fields])) OR ("Polymyositis"[MeSH Terms] OR "Polymyositis"[All Fields] OR ("idiopathic"[All Fields] AND "Polymyositis"[All Fields]) OR "idiopathic polymyositis"[All Fields]) OR ("Polymyositis"[MeSH Terms] OR "Polymyositis"[All Fields] OR ("polymyositides"[All Fields] AND "idiopathic"[All Fields]))) OR "Dermatomyositis"[MeSH Terms] OR ("Dermatomyositis"[MeSH Terms] OR "Dermatomyositis"[All Fields] OR ("Dermatomyositis"[MeSH Terms] OR "Dermatomyositis"[All Fields] OR ("Polymyositis"[All Fields] AND "Dermatomyositis"[All Fields]) OR "polymyositis dermatomyositis"[All Fields]) OR ("Dermatomyositis"[MeSH Terms] OR "Dermatomyositis"[All Fields] OR ("Polymyositis"[All Fields] AND "Dermatomyositis"[All Fields]) OR "polymyositis dermatomyositis"[All Fields]) OR ("Dermatomyositis"[MeSH Terms] OR "Dermatomyositis"[All Fields] OR "dermatopolymyositis"[All Fields]) OR ("Dermatomyositis"[MeSH Terms] OR "Dermatomyositis"[All Fields] OR ("Dermatomyositis"[All Fields] AND "adult"[All Fields] AND "type"[All Fields])) OR ("Dermatomyositis"[MeSH Terms] OR "Dermatomyositis"[All Fields] OR ("adult"[All Fields] AND "type"[All Fields] AND "Dermatomyositis"[All Fields]) OR "adult type dermatomyositis"[All Fields]) OR ("Dermatomyositis"[MeSH Terms] OR "Dermatomyositis"[All Fields] OR ("Dermatomyositis"[All Fields] AND "childhood"[All Fields] AND "type"[All Fields]) OR "dermatomyositis childhood type"[All Fields]) OR ("Dermatomyositis"[MeSH Terms] OR "Dermatomyositis"[All Fields] OR ("childhood"[All Fields] AND "type"[All Fields] AND "Dermatomyositis"[All Fields]) OR "childhood type dermatomyositis"[All Fields]) OR ("Dermatomyositis"[MeSH Terms] OR "Dermatomyositis"[All Fields] OR ("juvenile"[All Fields] AND "Dermatomyositis"[All Fields]) OR "juvenile dermatomyositis"[All Fields]) OR ("Dermatomyositis"[MeSH Terms] OR "Dermatomyositis"[All Fields] OR ("Dermatomyositis"[All Fields] AND "juvenile"[All Fields]) OR "dermatomyositis juvenile"[All Fields]) OR ("Dermatomyositis"[MeSH Terms] OR "Dermatomyositis"[All Fields] OR ("juvenile"[All Fields] AND "Myositis"[All Fields]) OR "juvenile myositis"[All Fields]) OR ("Dermatomyositis"[MeSH Terms] OR "Dermatomyositis"[All Fields] OR ("Myositis"[All Fields] AND "juvenile"[All Fields]) OR "myositis juvenile"[All Fields])) OR "Sjogren's Syndrome"[MeSH Terms] OR ("Sjogren's Syndrome"[MeSH Terms] OR ("sjogren s"[All Fields] AND "syndrome"[All Fields]) OR "Sjogren's Syndrome"[All Fields] OR ("sjogren"[All Fields] AND "syndrome"[All Fields]) OR "sjogren syndrome"[All Fields] OR ("Sjogren's Syndrome"[MeSH Terms] OR ("sjogren s"[All Fields] AND "syndrome"[All Fields]) OR "Sjogren's Syndrome"[All Fields] OR ("sjogren"[All Fields] AND "syndrome"[All Fields]) OR "sjogren syndrome"[All Fields]) OR ("Sjogren's Syndrome"[MeSH Terms] OR ("sjogren s"[All Fields] AND "syndrome"[All Fields]) OR "Sjogren's Syndrome"[All Fields] OR ("sjogrens"[All Fields] AND "syndrome"[All Fields]) OR "sjogrens syndrome"[All Fields]) OR ("Sjogren's Syndrome"[MeSH Terms] OR ("sjogren s"[All Fields] AND "syndrome"[All Fields]) OR "Sjogren's Syndrome"[All Fields] OR ("syndrome"[All Fields] AND "sjogren s"[All Fields]) OR "syndrome sjogren s"[All Fields]) OR ("Sjogren's Syndrome"[MeSH Terms] OR ("sjogren s"[All Fields] AND "syndrome"[All Fields]) OR "Sjogren's Syndrome"[All Fields] OR ("sjogren"[All Fields] AND "syndrome"[All Fields]) OR "sjogren syndrome"[All Fields]) OR ("Sjogren's Syndrome"[MeSH Terms] OR ("sjogren s"[All Fields] AND "syndrome"[All Fields]) OR "Sjogren's Syndrome"[All Fields] OR ("sicca"[All Fields] AND "syndrome"[All Fields]) OR "sicca syndrome"[All Fields]) OR ("Sjogren's Syndrome"[MeSH Terms] OR ("sjogren s"[All Fields] AND "syndrome"[All Fields]) OR "Sjogren's Syndrome"[All Fields] OR ("syndrome"[All Fields] AND "sicca"[All Fields]) OR "syndrome sicca"[All Fields])) OR "Mixed Connective Tissue Disease"[MeSH Terms] OR ("Mixed Connective Tissue Disease"[MeSH Terms] OR ("mixed"[All Fields] AND "connective"[All Fields] AND "tissue"[All Fields] AND "disease"[All Fields]) OR "Mixed Connective Tissue Disease"[All Fields] OR ("Mixed Connective Tissue Disease"[MeSH Terms] OR ("mixed"[All Fields] AND "connective"[All Fields] AND "tissue"[All Fields] AND "disease"[All Fields]) OR "Mixed Connective Tissue Disease"[All Fields] OR "mctd"[All Fields]) OR ("Mixed Connective Tissue Disease"[MeSH Terms] OR ("mixed"[All Fields] AND "connective"[All Fields] AND "tissue"[All Fields] AND "disease"[All Fields]) OR "Mixed Connective Tissue Disease"[All Fields] OR ("connective"[All Fields] AND "tissue"[All Fields] AND "disease"[All Fields] AND "mixed"[All Fields]) OR "connective tissue disease mixed"[All Fields]) OR ("Mixed Connective Tissue Disease"[MeSH Terms] OR ("mixed"[All Fields] AND "connective"[All Fields] AND "tissue"[All Fields] AND "disease"[All Fields]) OR "Mixed Connective Tissue Disease"[All Fields] OR ("sharp"[All Fields] AND "syndrome"[All Fields]) OR "sharp syndrome"[All Fields]) OR ("Mixed Connective Tissue Disease"[MeSH Terms] OR ("mixed"[All Fields] AND "connective"[All Fields] AND "tissue"[All Fields] AND "disease"[All Fields]) OR "Mixed Connective Tissue Disease"[All Fields] OR ("syndrome"[All Fields] AND "sharp"[All Fields]) OR "syndrome sharp"[All Fields])) OR "lupus erythematosus, systemic"[MeSH Terms] OR ("lupus erythematosus, systemic"[MeSH Terms] OR ("lupus"[All Fields] AND "erythematosus"[All Fields] AND "systemic"[All Fields]) OR "systemic lupus erythematosus"[All Fields] OR ("systemic"[All Fields] AND "lupus"[All Fields] AND "erythematosus"[All Fields]) OR ("lupus erythematosus, systemic"[MeSH Terms] OR ("lupus"[All Fields] AND "erythematosus"[All Fields] AND "systemic"[All Fields]) OR "systemic lupus erythematosus"[All Fields] OR ("lupus"[All Fields] AND "erythematosus"[All Fields] AND "disseminatus"[All Fields]) OR "lupus erythematosus disseminatus"[All Fields]) OR ("lupus erythematosus, systemic"[MeSH Terms] OR ("lupus"[All Fields] AND "erythematosus"[All Fields] AND "systemic"[All Fields]) OR "systemic lupus erythematosus"[All Fields] OR ("systemic"[All Fields] AND "lupus"[All Fields] AND "erythematosus"[All Fields])) OR ("lupus erythematosus, systemic"[MeSH Terms] OR ("lupus"[All Fields] AND "erythematosus"[All Fields] AND "systemic"[All Fields]) OR "systemic lupus erythematosus"[All Fields] OR ("libman"[All Fields] AND "sacks"[All Fields] AND "disease"[All Fields]) OR "libman sacks disease"[All Fields]) OR ("lupus erythematosus, systemic"[MeSH Terms] OR ("lupus"[All Fields] AND "erythematosus"[All Fields] AND "systemic"[All Fields]) OR "systemic lupus erythematosus"[All Fields] OR ("disease"[All Fields] AND "libman"[All Fields] AND "sacks"[All Fields]) OR "disease libman sacks"[All Fields]) OR ("lupus erythematosus, systemic"[MeSH Terms] OR ("lupus"[All Fields] AND "erythematosus"[All Fields] AND "systemic"[All Fields]) OR "systemic lupus erythematosus"[All Fields] OR ("libman"[All Fields] AND "sacks"[All Fields] AND "disease"[All Fields]) OR "libman sacks disease"[All Fields])) OR "Connective Tissue Diseases"[MeSH Terms] OR ("Connective Tissue Diseases"[MeSH Terms] OR ("connective"[All Fields] AND "tissue"[All Fields] AND "diseases"[All Fields]) OR "Connective Tissue Diseases"[All Fields] OR ("connective"[All Fields] AND "tissue"[All Fields] AND "disease"[All Fields]) OR "connective tissue disease"[All Fields] OR ("Connective Tissue Diseases"[MeSH Terms] OR ("connective"[All Fields] AND "tissue"[All Fields] AND "diseases"[All Fields]) OR "Connective Tissue Diseases"[All Fields] OR ("connective"[All Fields] AND "tissue"[All Fields] AND "disease"[All Fields]) OR "connective tissue disease"[All Fields]) OR ("Connective Tissue Diseases"[MeSH Terms] OR ("connective"[All Fields] AND "tissue"[All Fields] AND "diseases"[All Fields]) OR "Connective Tissue Diseases"[All Fields] OR ("disease"[All Fields] AND "connective"[All Fields] AND "tissue"[All Fields]) OR "disease connective tissue"[All Fields]) OR ("Connective Tissue Diseases"[MeSH Terms] OR ("connective"[All Fields] AND "tissue"[All Fields] AND "diseases"[All Fields]) OR "Connective Tissue Diseases"[All Fields] OR ("diseases"[All Fields] AND "connective"[All Fields] AND "tissue"[All Fields]) OR "diseases connective tissue"[All Fields]))) AND ("lung diseases, interstitial"[MeSH Terms] OR ("lung diseases, interstitial"[MeSH Terms] OR ("lung"[All Fields] AND "diseases"[All Fields] AND "interstitial"[All Fields]) OR "interstitial lung diseases"[All Fields] OR ("interstitial"[All Fields] AND "lung"[All Fields] AND "disease"[All Fields]) OR "interstitial lung disease"[All Fields] OR ("lung diseases, interstitial"[MeSH Terms] OR ("lung"[All Fields] AND "diseases"[All Fields] AND "interstitial"[All Fields]) OR "interstitial lung diseases"[All Fields] OR ("diffuse"[All Fields] AND "parenchymal"[All Fields] AND "lung"[All Fields] AND "diseases"[All Fields]) OR "diffuse parenchymal lung diseases"[All Fields]) OR ("lung diseases, interstitial"[MeSH Terms] OR ("lung"[All Fields] AND "diseases"[All Fields] AND "interstitial"[All Fields]) OR "interstitial lung diseases"[All Fields] OR ("interstitial"[All Fields] AND "lung"[All Fields] AND "diseases"[All Fields])) OR ("lung diseases, interstitial"[MeSH Terms] OR ("lung"[All Fields] AND "diseases"[All Fields] AND "interstitial"[All Fields]) OR "interstitial lung diseases"[All Fields] OR ("interstitial"[All Fields] AND "lung"[All Fields] AND "disease"[All Fields]) OR "interstitial lung disease"[All Fields]) OR ("lung diseases, interstitial"[MeSH Terms] OR ("lung"[All Fields] AND "diseases"[All Fields] AND "interstitial"[All Fields]) OR "interstitial lung diseases"[All Fields] OR ("lung"[All Fields] AND "disease"[All Fields] AND "interstitial"[All Fields]) OR "lung disease interstitial"[All Fields]) OR ("lung diseases, interstitial"[MeSH Terms] OR ("lung"[All Fields] AND "diseases"[All Fields] AND "interstitial"[All Fields]) OR "interstitial lung diseases"[All Fields] OR ("diffuse"[All Fields] AND "parenchymal"[All Fields] AND "lung"[All Fields] AND "disease"[All Fields]) OR "diffuse parenchymal lung disease"[All Fields]) OR ("lung diseases, interstitial"[MeSH Terms] OR ("lung"[All Fields] AND "diseases"[All Fields] AND "interstitial"[All Fields]) OR "interstitial lung diseases"[All Fields] OR ("pneumonia"[All Fields] AND "interstitial"[All Fields]) OR "pneumonia interstitial"[All Fields]) OR ("lung diseases, interstitial"[MeSH Terms] OR ("lung"[All Fields] AND "diseases"[All Fields] AND "interstitial"[All Fields]) OR "interstitial lung diseases"[All Fields] OR ("interstitial"[All Fields] AND "pneumonia"[All Fields]) OR "interstitial pneumonia"[All Fields]) OR ("lung diseases, interstitial"[MeSH Terms] OR ("lung"[All Fields] AND "diseases"[All Fields] AND "interstitial"[All Fields]) OR "interstitial lung diseases"[All Fields] OR ("interstitial"[All Fields] AND "pneumonias"[All Fields]) OR "interstitial pneumonias"[All Fields]) OR ("lung diseases, interstitial"[MeSH Terms] OR ("lung"[All Fields] AND "diseases"[All Fields] AND "interstitial"[All Fields]) OR "interstitial lung diseases"[All Fields] OR ("pneumonias"[All Fields] AND "interstitial"[All Fields]) OR "pneumonias interstitial"[All Fields]) OR ("lung diseases, interstitial"[MeSH Terms] OR ("lung"[All Fields] AND "diseases"[All Fields] AND "interstitial"[All Fields]) OR "interstitial lung diseases"[All Fields] OR ("pneumonitis"[All Fields] AND "interstitial"[All Fields]) OR "pneumonitis interstitial"[All Fields]) OR ("lung diseases, interstitial"[MeSH Terms] OR ("lung"[All Fields] AND "diseases"[All Fields] AND "interstitial"[All Fields]) OR "interstitial lung diseases"[All Fields] OR ("interstitial"[All Fields] AND "pneumonitides"[All Fields]) OR "interstitial pneumonitides"[All Fields]) OR ("lung diseases, interstitial"[MeSH Terms] OR ("lung"[All Fields] AND "diseases"[All Fields] AND "interstitial"[All Fields]) OR "interstitial lung diseases"[All Fields] OR ("interstitial"[All Fields] AND "pneumonitis"[All Fields]) OR "interstitial pneumonitis"[All Fields]) OR ("lung diseases, interstitial"[MeSH Terms] OR ("lung"[All Fields] AND "diseases"[All Fields] AND "interstitial"[All Fields]) OR "interstitial lung diseases"[All Fields] OR ("pneumonitides"[All Fields] AND "interstitial"[All Fields])))) AND ("Symptom Flare Up"[MeSH Terms] OR ("exacerbate"[All Fields] OR "exacerbated"[All Fields] OR "exacerbates"[All Fields] OR "exacerbating"[All Fields] OR "exacerbation"[All Fields] OR "exacerbations"[All Fields] OR "exacerbator"[All Fields] OR "exacerbators"[All Fields] OR ("Symptom Flare Up"[MeSH Terms] OR ("symptom"[All Fields] AND "flare"[All Fields] AND "up"[All Fields]) OR "Symptom Flare Up"[All Fields]) OR ("Symptom Flare Up"[MeSH Terms] OR ("symptom"[All Fields] AND "flare"[All Fields] AND "up"[All Fields]) OR "Symptom Flare Up"[All Fields] OR ("flare"[All Fields] AND "ups"[All Fields] AND "symptom"[All Fields])) OR ("Symptom Flare Up"[MeSH Terms] OR ("symptom"[All Fields] AND "flare"[All Fields] AND "up"[All Fields]) OR "Symptom Flare Up"[All Fields] OR ("flare"[All Fields] AND "up"[All Fields] AND "symptom"[All Fields]) OR "flare up symptom"[All Fields]) OR ("Symptom Flare Up"[MeSH Terms] OR ("symptom"[All Fields] AND "flare"[All Fields] AND "up"[All Fields]) OR "Symptom Flare Up"[All Fields] OR ("symptom"[All Fields] AND "flare"[All Fields] AND "ups"[All Fields]) OR "symptom flare ups"[All Fields]) OR ("Symptom Flare Up"[MeSH Terms] OR ("symptom"[All Fields] AND "flare"[All Fields] AND "up"[All Fields]) OR "Symptom Flare Up"[All Fields] OR ("symptom"[All Fields] AND "flareup"[All Fields])) OR ("Symptom Flare Up"[MeSH Terms] OR ("symptom"[All Fields] AND "flare"[All Fields] AND "up"[All Fields]) OR "Symptom Flare Up"[All Fields] OR ("flareups"[All Fields] AND "symptom"[All Fields])) OR ("Symptom Flare Up"[MeSH Terms] OR ("symptom"[All Fields] AND "flare"[All Fields] AND "up"[All Fields]) OR "Symptom Flare Up"[All Fields] OR ("flareup"[All Fields] AND "symptom"[All Fields])) OR ("Symptom Flare Up"[MeSH Terms] OR ("symptom"[All Fields] AND "flare"[All Fields] AND "up"[All Fields]) OR "Symptom Flare Up"[All Fields] OR ("symptom"[All Fields] AND "flareups"[All Fields])) OR ("Symptom Flare Up"[MeSH Terms] OR ("symptom"[All Fields] AND "flare"[All Fields] AND "up"[All Fields]) OR "Symptom Flare Up"[All Fields]) OR ("Symptom Flare Up"[MeSH Terms] OR ("symptom"[All Fields] AND "flare"[All Fields] AND "up"[All Fields]) OR "Symptom Flare Up"[All Fields] OR ("flare"[All Fields] AND "ups"[All Fields] AND "symptom"[All Fields])) OR ("Symptom Flare Up"[MeSH Terms] OR ("symptom"[All Fields] AND "flare"[All Fields] AND "up"[All Fields]) OR "Symptom Flare Up"[All Fields] OR ("flare"[All Fields] AND "up"[All Fields] AND "symptom"[All Fields]) OR "flare up symptom"[All Fields]) OR ("Symptom Flare Up"[MeSH Terms] OR ("symptom"[All Fields] AND "flare"[All Fields] AND "up"[All Fields]) OR "Symptom Flare Up"[All Fields] OR ("symptom"[All Fields] AND "flare"[All Fields] AND "ups"[All Fields]) OR "symptom flare ups"[All Fields]) OR ("Symptom Flare Up"[MeSH Terms] OR ("symptom"[All Fields] AND "flare"[All Fields] AND "up"[All Fields]) OR "Symptom Flare Up"[All Fields] OR ("acute"[All Fields] AND "symptom"[All Fields] AND "flare"[All Fields]) OR "acute symptom flare"[All Fields]) OR ("Symptom Flare Up"[MeSH Terms] OR ("symptom"[All Fields] AND "flare"[All Fields] AND "up"[All Fields]) OR "Symptom Flare Up"[All Fields] OR ("acute"[All Fields] AND "symptom"[All Fields] AND "flares"[All Fields]) OR "acute symptom flares"[All Fields]) OR ("Symptom Flare Up"[MeSH Terms] OR ("symptom"[All Fields] AND "flare"[All Fields] AND "up"[All Fields]) OR "Symptom Flare Up"[All Fields] OR ("symptom"[All Fields] AND "flare"[All Fields] AND "acute"[All Fields])) OR ("Symptom Flare Up"[MeSH Terms] OR ("symptom"[All Fields] AND "flare"[All Fields] AND "up"[All Fields]) OR "Symptom Flare Up"[All Fields] OR ("symptom"[All Fields] AND "flaring"[All Fields] AND "up"[All Fields])) OR ("Symptom Flare Up"[MeSH Terms] OR ("symptom"[All Fields] AND "flare"[All Fields] AND "up"[All Fields]) OR "Symptom Flare Up"[All Fields] OR ("flaring"[All Fields] AND "up"[All Fields] AND "symptom"[All Fields])) OR ("Symptom Flare Up"[MeSH Terms] OR ("symptom"[All Fields] AND "flare"[All Fields] AND "up"[All Fields]) OR "Symptom Flare Up"[All Fields] OR ("symptom"[All Fields] AND "exacerbation"[All Fields]) OR "symptom exacerbation"[All Fields]) OR ("Symptom Flare Up"[MeSH Terms] OR ("symptom"[All Fields] AND "flare"[All Fields] AND "up"[All Fields]) OR "Symptom Flare Up"[All Fields] OR ("exacerbation"[All Fields] AND "symptom"[All Fields]) OR "exacerbation symptom"[All Fields]) OR ("Symptom Flare Up"[MeSH Terms] OR ("symptom"[All Fields] AND "flare"[All Fields] AND "up"[All Fields]) OR "Symptom Flare Up"[All Fields] OR ("symptom"[All Fields] AND "exacerbations"[All Fields]) OR "symptom exacerbations"[All Fields]) OR ("Symptom Flare Up"[MeSH Terms] OR ("symptom"[All Fields] AND "flare"[All Fields] AND "up"[All Fields]) OR "Symptom Flare Up"[All Fields] OR ("symptom"[All Fields] AND "worsening"[All Fields]) OR "symptom worsening"[All Fields]) OR ("Symptom Flare Up"[MeSH Terms] OR ("symptom"[All Fields] AND "flare"[All Fields] AND "up"[All Fields]) OR "Symptom Flare Up"[All Fields] OR ("worsening"[All Fields] AND "symptom"[All Fields]) OR "worsening symptom"[All Fields]) OR ("Symptom Flare Up"[MeSH Terms] OR ("symptom"[All Fields] AND "flare"[All Fields] AND "up"[All Fields]) OR "Symptom Flare Up"[All Fields] OR ("symptom"[All Fields] AND "exaggeration"[All Fields]) OR "symptom exaggeration"[All Fields]) OR ("Symptom Flare Up"[MeSH Terms] OR ("symptom"[All Fields] AND "flare"[All Fields] AND "up"[All Fields]) OR "Symptom Flare Up"[All Fields] OR ("exaggeration"[All Fields] AND "symptom"[All Fields]) OR "exaggeration symptom"[All Fields]) OR ("Symptom Flare Up"[MeSH Terms] OR ("symptom"[All Fields] AND "flare"[All Fields] AND "up"[All Fields]) OR "Symptom Flare Up"[All Fields] OR ("symptom"[All Fields] AND "exaggerations"[All Fields])) OR ("Symptom Flare Up"[MeSH Terms] OR ("symptom"[All Fields] AND "flare"[All Fields] AND "up"[All Fields]) OR "Symptom Flare Up"[All Fields] OR ("symptom"[All Fields] AND "magnification"[All Fields]) OR "symptom magnification"[All Fields]) OR ("Symptom Flare Up"[MeSH Terms] OR ("symptom"[All Fields] AND "flare"[All Fields] AND "up"[All Fields]) OR "Symptom Flare Up"[All Fields] OR ("magnification"[All Fields] AND "symptom"[All Fields]) OR "magnification symptom"[All Fields]) OR ("Symptom Flare Up"[MeSH Terms] OR ("symptom"[All Fields] AND "flare"[All Fields] AND "up"[All Fields]) OR "Symptom Flare Up"[All Fields] OR ("symptom"[All Fields] AND "magnifications"[All Fields])) OR ("Symptom Flare Up"[MeSH Terms] OR ("symptom"[All Fields] AND "flare"[All Fields] AND "up"[All Fields]) OR "Symptom Flare Up"[All Fields] OR ("symptom"[All Fields] AND "increase"[All Fields]) OR "symptom increase"[All Fields]) OR ("Symptom Flare Up"[MeSH Terms] OR ("symptom"[All Fields] AND "flare"[All Fields] AND "up"[All Fields]) OR "Symptom Flare Up"[All Fields] OR ("increase"[All Fields] AND "symptom"[All Fields]) OR "increase symptom"[All Fields])) OR "Disease Progression"[MeSH Terms] OR ("Disease Progression"[MeSH Terms] OR ("disease"[All Fields] AND "progression"[All Fields]) OR "Disease Progression"[All Fields] OR ("Disease Progression"[MeSH Terms] OR ("disease"[All Fields] AND "progression"[All Fields]) OR "Disease Progression"[All Fields] OR ("progression"[All Fields] AND "disease"[All Fields]) OR "progression disease"[All Fields]) OR ("Disease Progression"[MeSH Terms] OR ("disease"[All Fields] AND "progression"[All Fields]) OR "Disease Progression"[All Fields] OR ("disease"[All Fields] AND "exacerbation"[All Fields]) OR "disease exacerbation"[All Fields]) OR ("Disease Progression"[MeSH Terms] OR ("disease"[All Fields] AND "progression"[All Fields]) OR "Disease Progression"[All Fields] OR ("exacerbation"[All Fields] AND "disease"[All Fields]) OR "exacerbation disease"[All Fields]) OR ("Disease Progression"[MeSH Terms] OR ("disease"[All Fields] AND "progression"[All Fields]) OR "Disease Progression"[All Fields] OR ("clinical"[All Fields] AND "course"[All Fields]) OR "clinical course"[All Fields]) OR ("Disease Progression"[MeSH Terms] OR ("disease"[All Fields] AND "progression"[All Fields]) OR "Disease Progression"[All Fields] OR ("clinical"[All Fields] AND "progression"[All Fields]) OR "clinical progression"[All Fields]) OR ("Disease Progression"[MeSH Terms] OR ("disease"[All Fields] AND "progression"[All Fields]) OR "Disease Progression"[All Fields] OR ("progression"[All Fields] AND "clinical"[All Fields]) OR "progression clinical"[All Fields])) OR "Clinical Deterioration"[MeSH Terms] OR ("deteriorate"[All Fields] OR "deteriorated"[All Fields] OR "deteriorates"[All Fields] OR "deteriorating"[All Fields] OR "deterioration"[All Fields] OR "deteriorations"[All Fields] OR "deteriorative"[All Fields] OR ("Clinical Deterioration"[MeSH Terms] OR ("clinical"[All Fields] AND "deterioration"[All Fields]) OR "Clinical Deterioration"[All Fields]) OR ("Clinical Deterioration"[MeSH Terms] OR ("clinical"[All Fields] AND "deterioration"[All Fields]) OR "Clinical Deterioration"[All Fields] OR ("clinical"[All Fields] AND "deteriorations"[All Fields]) OR "clinical deteriorations"[All Fields]) OR ("Clinical Deterioration"[MeSH Terms] OR ("clinical"[All Fields] AND "deterioration"[All Fields]) OR "Clinical Deterioration"[All Fields] OR ("deterioration"[All Fields] AND "clinical"[All Fields]) OR "deterioration clinical"[All Fields])) OR ((("rapid"[All Fields] OR "rapidities"[All Fields] OR "rapidity"[All Fields] OR "rapidness"[All Fields]) AND ("Disease Progression"[MeSH Terms] OR ("disease"[All Fields] AND "progression"[All Fields]) OR "Disease Progression"[All Fields] OR "progression"[All Fields] OR "progress"[All Fields] OR "progressed"[All Fields] OR "progresses"[All Fields] OR "progressing"[All Fields] OR "progressions"[All Fields] OR "progressive"[All Fields] OR "progressively"[All Fields] OR "progressives"[All Fields])) OR (("acute"[All Fields] OR "acutely"[All Fields] OR "acutes"[All Fields]) AND ("exacerbate"[All Fields] OR "exacerbated"[All Fields] OR "exacerbates"[All Fields] OR "exacerbating"[All Fields] OR "exacerbation"[All Fields] OR "exacerbations"[All Fields] OR "exacerbator"[All Fields] OR "exacerbators"[All Fields])) OR ("Clinical Deterioration"[MeSH Terms] OR ("clinical"[All Fields] AND "deterioration"[All Fields]) OR "Clinical Deterioration"[All Fields]) OR ("exacerbate"[All Fields] OR "exacerbated"[All Fields] OR "exacerbates"[All Fields] OR "exacerbating"[All Fields] OR "exacerbation"[All Fields] OR "exacerbations"[All Fields] OR "exacerbator"[All Fields] OR "exacerbators"[All Fields]) OR ("course"[All Fields] OR "course s"[All Fields] OR "courses"[All Fields]) OR ("Disease Progression"[MeSH Terms] OR ("disease"[All Fields] AND "progression"[All Fields]) OR "Disease Progression"[All Fields] OR "progression"[All Fields] OR "progress"[All Fields] OR "progressed"[All Fields] OR "progresses"[All Fields] OR "progressing"[All Fields] OR "progressions"[All Fields] OR "progressive"[All Fields] OR "progressively"[All Fields] OR "progressives"[All Fields]) OR ("Disease Progression"[MeSH Terms] OR ("disease"[All Fields] AND "progression"[All Fields]) OR "Disease Progression"[All Fields] OR "progression"[All Fields] OR "progress"[All Fields] OR "progressed"[All Fields] OR "progresses"[All Fields] OR "progressing"[All Fields] OR "progressions"[All Fields] OR "progressive"[All Fields] OR "progressively"[All Fields] OR "progressives"[All Fields]) OR ("Disease Progression"[MeSH Terms] OR ("disease"[All Fields] AND "progression"[All Fields]) OR "Disease Progression"[All Fields] OR "progression"[All Fields] OR "progress"[All Fields] OR "progressed"[All Fields] OR "progresses"[All Fields] OR "progressing"[All Fields] OR "progressions"[All Fields] OR "progressive"[All Fields] OR "progressively"[All Fields] OR "progressives"[All Fields]))) | 4,420 |
| 30 | (((((("Symptom Flare Up"[Mesh]) OR (((((((((((((((((((((((((((((((exacerbation) OR (Symptom Flare Up)) OR (Flare Ups, Symptom)) OR (Flare Up, Symptom)) OR (Symptom Flare Ups)) OR (Symptom Flareup)) OR (Flareups, Symptom)) OR (Flareup, Symptom)) OR (Symptom Flareups)) OR (Symptom Flare-up)) OR (Flare-ups, Symptom)) OR (Flare-up, Symptom)) OR (Symptom Flare-ups)) OR (Acute Symptom Flare)) OR (Acute Symptom Flares)) OR (Symptom Flare, Acute)) OR (Symptom Flaring Up)) OR (Flaring Up, Symptom)) OR (Symptom Exacerbation)) OR (Exacerbation, Symptom)) OR (Symptom Exacerbations)) OR (Symptom Worsening)) OR (Worsening, Symptom)) OR (Symptom Exaggeration)) OR (Exaggeration, Symptom)) OR (Symptom Exaggerations)) OR (Symptom Magnification)) OR (Magnification, Symptom)) OR (Symptom Magnifications)) OR (Symptom Increase)) OR (Increase, Symptom))) OR ("Disease Progression"[Mesh])) OR (((((((Disease Progression) OR (Progression, Disease)) OR (Disease Exacerbation)) OR (Exacerbation, Disease)) OR (Clinical Course)) OR (Clinical Progression)) OR (Progression, Clinical))) OR ("Clinical Deterioration"[Mesh])) OR ((((deterioration) OR (Clinical Deterioration)) OR (Clinical Deteriorations)) OR (Deterioration, Clinical))) OR ((((((((Rapid Progress) OR (Acute Exacerbation)) OR (Clinical Deterioration)) OR (Exacerbation)) OR (Course)) OR (Progression)) OR (Progress)) OR (Progressive)) | "Symptom Flare Up"[MeSH Terms] OR ("exacerbate"[All Fields] OR "exacerbated"[All Fields] OR "exacerbates"[All Fields] OR "exacerbating"[All Fields] OR "exacerbation"[All Fields] OR "exacerbations"[All Fields] OR "exacerbator"[All Fields] OR "exacerbators"[All Fields] OR ("Symptom Flare Up"[MeSH Terms] OR ("symptom"[All Fields] AND "flare"[All Fields] AND "up"[All Fields]) OR "Symptom Flare Up"[All Fields]) OR ("Symptom Flare Up"[MeSH Terms] OR ("symptom"[All Fields] AND "flare"[All Fields] AND "up"[All Fields]) OR "Symptom Flare Up"[All Fields] OR ("flare"[All Fields] AND "ups"[All Fields] AND "symptom"[All Fields])) OR ("Symptom Flare Up"[MeSH Terms] OR ("symptom"[All Fields] AND "flare"[All Fields] AND "up"[All Fields]) OR "Symptom Flare Up"[All Fields] OR ("flare"[All Fields] AND "up"[All Fields] AND "symptom"[All Fields]) OR "flare up symptom"[All Fields]) OR ("Symptom Flare Up"[MeSH Terms] OR ("symptom"[All Fields] AND "flare"[All Fields] AND "up"[All Fields]) OR "Symptom Flare Up"[All Fields] OR ("symptom"[All Fields] AND "flare"[All Fields] AND "ups"[All Fields]) OR "symptom flare ups"[All Fields]) OR ("Symptom Flare Up"[MeSH Terms] OR ("symptom"[All Fields] AND "flare"[All Fields] AND "up"[All Fields]) OR "Symptom Flare Up"[All Fields] OR ("symptom"[All Fields] AND "flareup"[All Fields])) OR ("Symptom Flare Up"[MeSH Terms] OR ("symptom"[All Fields] AND "flare"[All Fields] AND "up"[All Fields]) OR "Symptom Flare Up"[All Fields] OR ("flareups"[All Fields] AND "symptom"[All Fields])) OR ("Symptom Flare Up"[MeSH Terms] OR ("symptom"[All Fields] AND "flare"[All Fields] AND "up"[All Fields]) OR "Symptom Flare Up"[All Fields] OR ("flareup"[All Fields] AND "symptom"[All Fields])) OR ("Symptom Flare Up"[MeSH Terms] OR ("symptom"[All Fields] AND "flare"[All Fields] AND "up"[All Fields]) OR "Symptom Flare Up"[All Fields] OR ("symptom"[All Fields] AND "flareups"[All Fields])) OR ("Symptom Flare Up"[MeSH Terms] OR ("symptom"[All Fields] AND "flare"[All Fields] AND "up"[All Fields]) OR "Symptom Flare Up"[All Fields]) OR ("Symptom Flare Up"[MeSH Terms] OR ("symptom"[All Fields] AND "flare"[All Fields] AND "up"[All Fields]) OR "Symptom Flare Up"[All Fields] OR ("flare"[All Fields] AND "ups"[All Fields] AND "symptom"[All Fields])) OR ("Symptom Flare Up"[MeSH Terms] OR ("symptom"[All Fields] AND "flare"[All Fields] AND "up"[All Fields]) OR "Symptom Flare Up"[All Fields] OR ("flare"[All Fields] AND "up"[All Fields] AND "symptom"[All Fields]) OR "flare up symptom"[All Fields]) OR ("Symptom Flare Up"[MeSH Terms] OR ("symptom"[All Fields] AND "flare"[All Fields] AND "up"[All Fields]) OR "Symptom Flare Up"[All Fields] OR ("symptom"[All Fields] AND "flare"[All Fields] AND "ups"[All Fields]) OR "symptom flare ups"[All Fields]) OR ("Symptom Flare Up"[MeSH Terms] OR ("symptom"[All Fields] AND "flare"[All Fields] AND "up"[All Fields]) OR "Symptom Flare Up"[All Fields] OR ("acute"[All Fields] AND "symptom"[All Fields] AND "flare"[All Fields]) OR "acute symptom flare"[All Fields]) OR ("Symptom Flare Up"[MeSH Terms] OR ("symptom"[All Fields] AND "flare"[All Fields] AND "up"[All Fields]) OR "Symptom Flare Up"[All Fields] OR ("acute"[All Fields] AND "symptom"[All Fields] AND "flares"[All Fields]) OR "acute symptom flares"[All Fields]) OR ("Symptom Flare Up"[MeSH Terms] OR ("symptom"[All Fields] AND "flare"[All Fields] AND "up"[All Fields]) OR "Symptom Flare Up"[All Fields] OR ("symptom"[All Fields] AND "flare"[All Fields] AND "acute"[All Fields])) OR ("Symptom Flare Up"[MeSH Terms] OR ("symptom"[All Fields] AND "flare"[All Fields] AND "up"[All Fields]) OR "Symptom Flare Up"[All Fields] OR ("symptom"[All Fields] AND "flaring"[All Fields] AND "up"[All Fields])) OR ("Symptom Flare Up"[MeSH Terms] OR ("symptom"[All Fields] AND "flare"[All Fields] AND "up"[All Fields]) OR "Symptom Flare Up"[All Fields] OR ("flaring"[All Fields] AND "up"[All Fields] AND "symptom"[All Fields])) OR ("Symptom Flare Up"[MeSH Terms] OR ("symptom"[All Fields] AND "flare"[All Fields] AND "up"[All Fields]) OR "Symptom Flare Up"[All Fields] OR ("symptom"[All Fields] AND "exacerbation"[All Fields]) OR "symptom exacerbation"[All Fields]) OR ("Symptom Flare Up"[MeSH Terms] OR ("symptom"[All Fields] AND "flare"[All Fields] AND "up"[All Fields]) OR "Symptom Flare Up"[All Fields] OR ("exacerbation"[All Fields] AND "symptom"[All Fields]) OR "exacerbation symptom"[All Fields]) OR ("Symptom Flare Up"[MeSH Terms] OR ("symptom"[All Fields] AND "flare"[All Fields] AND "up"[All Fields]) OR "Symptom Flare Up"[All Fields] OR ("symptom"[All Fields] AND "exacerbations"[All Fields]) OR "symptom exacerbations"[All Fields]) OR ("Symptom Flare Up"[MeSH Terms] OR ("symptom"[All Fields] AND "flare"[All Fields] AND "up"[All Fields]) OR "Symptom Flare Up"[All Fields] OR ("symptom"[All Fields] AND "worsening"[All Fields]) OR "symptom worsening"[All Fields]) OR ("Symptom Flare Up"[MeSH Terms] OR ("symptom"[All Fields] AND "flare"[All Fields] AND "up"[All Fields]) OR "Symptom Flare Up"[All Fields] OR ("worsening"[All Fields] AND "symptom"[All Fields]) OR "worsening symptom"[All Fields]) OR ("Symptom Flare Up"[MeSH Terms] OR ("symptom"[All Fields] AND "flare"[All Fields] AND "up"[All Fields]) OR "Symptom Flare Up"[All Fields] OR ("symptom"[All Fields] AND "exaggeration"[All Fields]) OR "symptom exaggeration"[All Fields]) OR ("Symptom Flare Up"[MeSH Terms] OR ("symptom"[All Fields] AND "flare"[All Fields] AND "up"[All Fields]) OR "Symptom Flare Up"[All Fields] OR ("exaggeration"[All Fields] AND "symptom"[All Fields]) OR "exaggeration symptom"[All Fields]) OR ("Symptom Flare Up"[MeSH Terms] OR ("symptom"[All Fields] AND "flare"[All Fields] AND "up"[All Fields]) OR "Symptom Flare Up"[All Fields] OR ("symptom"[All Fields] AND "exaggerations"[All Fields])) OR ("Symptom Flare Up"[MeSH Terms] OR ("symptom"[All Fields] AND "flare"[All Fields] AND "up"[All Fields]) OR "Symptom Flare Up"[All Fields] OR ("symptom"[All Fields] AND "magnification"[All Fields]) OR "symptom magnification"[All Fields]) OR ("Symptom Flare Up"[MeSH Terms] OR ("symptom"[All Fields] AND "flare"[All Fields] AND "up"[All Fields]) OR "Symptom Flare Up"[All Fields] OR ("magnification"[All Fields] AND "symptom"[All Fields]) OR "magnification symptom"[All Fields]) OR ("Symptom Flare Up"[MeSH Terms] OR ("symptom"[All Fields] AND "flare"[All Fields] AND "up"[All Fields]) OR "Symptom Flare Up"[All Fields] OR ("symptom"[All Fields] AND "magnifications"[All Fields])) OR ("Symptom Flare Up"[MeSH Terms] OR ("symptom"[All Fields] AND "flare"[All Fields] AND "up"[All Fields]) OR "Symptom Flare Up"[All Fields] OR ("symptom"[All Fields] AND "increase"[All Fields]) OR "symptom increase"[All Fields]) OR ("Symptom Flare Up"[MeSH Terms] OR ("symptom"[All Fields] AND "flare"[All Fields] AND "up"[All Fields]) OR "Symptom Flare Up"[All Fields] OR ("increase"[All Fields] AND "symptom"[All Fields]) OR "increase symptom"[All Fields])) OR "Disease Progression"[MeSH Terms] OR ("Disease Progression"[MeSH Terms] OR ("disease"[All Fields] AND "progression"[All Fields]) OR "Disease Progression"[All Fields] OR ("Disease Progression"[MeSH Terms] OR ("disease"[All Fields] AND "progression"[All Fields]) OR "Disease Progression"[All Fields] OR ("progression"[All Fields] AND "disease"[All Fields]) OR "progression disease"[All Fields]) OR ("Disease Progression"[MeSH Terms] OR ("disease"[All Fields] AND "progression"[All Fields]) OR "Disease Progression"[All Fields] OR ("disease"[All Fields] AND "exacerbation"[All Fields]) OR "disease exacerbation"[All Fields]) OR ("Disease Progression"[MeSH Terms] OR ("disease"[All Fields] AND "progression"[All Fields]) OR "Disease Progression"[All Fields] OR ("exacerbation"[All Fields] AND "disease"[All Fields]) OR "exacerbation disease"[All Fields]) OR ("Disease Progression"[MeSH Terms] OR ("disease"[All Fields] AND "progression"[All Fields]) OR "Disease Progression"[All Fields] OR ("clinical"[All Fields] AND "course"[All Fields]) OR "clinical course"[All Fields]) OR ("Disease Progression"[MeSH Terms] OR ("disease"[All Fields] AND "progression"[All Fields]) OR "Disease Progression"[All Fields] OR ("clinical"[All Fields] AND "progression"[All Fields]) OR "clinical progression"[All Fields]) OR ("Disease Progression"[MeSH Terms] OR ("disease"[All Fields] AND "progression"[All Fields]) OR "Disease Progression"[All Fields] OR ("progression"[All Fields] AND "clinical"[All Fields]) OR "progression clinical"[All Fields])) OR "Clinical Deterioration"[MeSH Terms] OR ("deteriorate"[All Fields] OR "deteriorated"[All Fields] OR "deteriorates"[All Fields] OR "deteriorating"[All Fields] OR "deterioration"[All Fields] OR "deteriorations"[All Fields] OR "deteriorative"[All Fields] OR ("Clinical Deterioration"[MeSH Terms] OR ("clinical"[All Fields] AND "deterioration"[All Fields]) OR "Clinical Deterioration"[All Fields]) OR ("Clinical Deterioration"[MeSH Terms] OR ("clinical"[All Fields] AND "deterioration"[All Fields]) OR "Clinical Deterioration"[All Fields] OR ("clinical"[All Fields] AND "deteriorations"[All Fields]) OR "clinical deteriorations"[All Fields]) OR ("Clinical Deterioration"[MeSH Terms] OR ("clinical"[All Fields] AND "deterioration"[All Fields]) OR "Clinical Deterioration"[All Fields] OR ("deterioration"[All Fields] AND "clinical"[All Fields]) OR "deterioration clinical"[All Fields])) OR ((("rapid"[All Fields] OR "rapidities"[All Fields] OR "rapidity"[All Fields] OR "rapidness"[All Fields]) AND ("Disease Progression"[MeSH Terms] OR ("disease"[All Fields] AND "progression"[All Fields]) OR "Disease Progression"[All Fields] OR "progression"[All Fields] OR "progress"[All Fields] OR "progressed"[All Fields] OR "progresses"[All Fields] OR "progressing"[All Fields] OR "progressions"[All Fields] OR "progressive"[All Fields] OR "progressively"[All Fields] OR "progressives"[All Fields])) OR (("acute"[All Fields] OR "acutely"[All Fields] OR "acutes"[All Fields]) AND ("exacerbate"[All Fields] OR "exacerbated"[All Fields] OR "exacerbates"[All Fields] OR "exacerbating"[All Fields] OR "exacerbation"[All Fields] OR "exacerbations"[All Fields] OR "exacerbator"[All Fields] OR "exacerbators"[All Fields])) OR ("Clinical Deterioration"[MeSH Terms] OR ("clinical"[All Fields] AND "deterioration"[All Fields]) OR "Clinical Deterioration"[All Fields]) OR ("exacerbate"[All Fields] OR "exacerbated"[All Fields] OR "exacerbates"[All Fields] OR "exacerbating"[All Fields] OR "exacerbation"[All Fields] OR "exacerbations"[All Fields] OR "exacerbator"[All Fields] OR "exacerbators"[All Fields]) OR ("course"[All Fields] OR "course s"[All Fields] OR "courses"[All Fields]) OR ("Disease Progression"[MeSH Terms] OR ("disease"[All Fields] AND "progression"[All Fields]) OR "Disease Progression"[All Fields] OR "progression"[All Fields] OR "progress"[All Fields] OR "progressed"[All Fields] OR "progresses"[All Fields] OR "progressing"[All Fields] OR "progressions"[All Fields] OR "progressive"[All Fields] OR "progressively"[All Fields] OR "progressives"[All Fields]) OR ("Disease Progression"[MeSH Terms] OR ("disease"[All Fields] AND "progression"[All Fields]) OR "Disease Progression"[All Fields] OR "progression"[All Fields] OR "progress"[All Fields] OR "progressed"[All Fields] OR "progresses"[All Fields] OR "progressing"[All Fields] OR "progressions"[All Fields] OR "progressive"[All Fields] OR "progressively"[All Fields] OR "progressives"[All Fields]) OR ("Disease Progression"[MeSH Terms] OR ("disease"[All Fields] AND "progression"[All Fields]) OR "Disease Progression"[All Fields] OR "progression"[All Fields] OR "progress"[All Fields] OR "progressed"[All Fields] OR "progresses"[All Fields] OR "progressing"[All Fields] OR "progressions"[All Fields] OR "progressive"[All Fields] OR "progressively"[All Fields] OR "progressives"[All Fields])) | 2,772,550 |
| 29 | (((((((Rapid Progress) OR (Acute Exacerbation)) OR (Clinical Deterioration)) OR (Exacerbation)) OR (Course)) OR (Progression)) OR (Progress)) OR (Progressive) | (("rapid"[All Fields] OR "rapidities"[All Fields] OR "rapidity"[All Fields] OR "rapidness"[All Fields]) AND ("disease progression"[MeSH Terms] OR ("disease"[All Fields] AND "progression"[All Fields]) OR "disease progression"[All Fields] OR "progression"[All Fields] OR "progress"[All Fields] OR "progressed"[All Fields] OR "progresses"[All Fields] OR "progressing"[All Fields] OR "progressions"[All Fields] OR "progressive"[All Fields] OR "progressively"[All Fields] OR "progressives"[All Fields])) OR (("acute"[All Fields] OR "acutely"[All Fields] OR "acutes"[All Fields]) AND ("exacerbate"[All Fields] OR "exacerbated"[All Fields] OR "exacerbates"[All Fields] OR "exacerbating"[All Fields] OR "exacerbation"[All Fields] OR "exacerbations"[All Fields] OR "exacerbator"[All Fields] OR "exacerbators"[All Fields])) OR ("clinical deterioration"[MeSH Terms] OR ("clinical"[All Fields] AND "deterioration"[All Fields]) OR "clinical deterioration"[All Fields]) OR ("exacerbate"[All Fields] OR "exacerbated"[All Fields] OR "exacerbates"[All Fields] OR "exacerbating"[All Fields] OR "exacerbation"[All Fields] OR "exacerbations"[All Fields] OR "exacerbator"[All Fields] OR "exacerbators"[All Fields]) OR ("course"[All Fields] OR "course s"[All Fields] OR "courses"[All Fields]) OR ("disease progression"[MeSH Terms] OR ("disease"[All Fields] AND "progression"[All Fields]) OR "disease progression"[All Fields] OR "progression"[All Fields] OR "progress"[All Fields] OR "progressed"[All Fields] OR "progresses"[All Fields] OR "progressing"[All Fields] OR "progressions"[All Fields] OR "progressive"[All Fields] OR "progressively"[All Fields] OR "progressives"[All Fields]) OR ("disease progression"[MeSH Terms] OR ("disease"[All Fields] AND "progression"[All Fields]) OR "disease progression"[All Fields] OR "progression"[All Fields] OR "progress"[All Fields] OR "progressed"[All Fields] OR "progresses"[All Fields] OR "progressing"[All Fields] OR "progressions"[All Fields] OR "progressive"[All Fields] OR "progressively"[All Fields] OR "progressives"[All Fields]) OR ("disease progression"[MeSH Terms] OR ("disease"[All Fields] AND "progression"[All Fields]) OR "disease progression"[All Fields] OR "progression"[All Fields] OR "progress"[All Fields] OR "progressed"[All Fields] OR "progresses"[All Fields] OR "progressing"[All Fields] OR "progressions"[All Fields] OR "progressive"[All Fields] OR "progressively"[All Fields] OR "progressives"[All Fields]) | 2,645,536 |
| 28 | (((deterioration) OR (Clinical Deterioration)) OR (Clinical Deteriorations)) OR (Deterioration, Clinical) | "deteriorate"[All Fields] OR "deteriorated"[All Fields] OR "deteriorates"[All Fields] OR "deteriorating"[All Fields] OR "deterioration"[All Fields] OR "deteriorations"[All Fields] OR "deteriorative"[All Fields] OR ("clinical deterioration"[MeSH Terms] OR ("clinical"[All Fields] AND "deterioration"[All Fields]) OR "clinical deterioration"[All Fields]) OR ("clinical deterioration"[MeSH Terms] OR ("clinical"[All Fields] AND "deterioration"[All Fields]) OR "clinical deterioration"[All Fields] OR ("clinical"[All Fields] AND "deteriorations"[All Fields]) OR "clinical deteriorations"[All Fields]) OR ("clinical deterioration"[MeSH Terms] OR ("clinical"[All Fields] AND "deterioration"[All Fields]) OR "clinical deterioration"[All Fields] OR ("deterioration"[All Fields] AND "clinical"[All Fields]) OR "deterioration clinical"[All Fields]) | 180,920 |
| 27 | "Clinical Deterioration"[Mesh] | "Clinical Deterioration"[MeSH Terms] | 841 |
| 26 | ((((((Disease Progression) OR (Progression, Disease)) OR (Disease Exacerbation)) OR (Exacerbation, Disease)) OR (Clinical Course)) OR (Clinical Progression)) OR (Progression, Clinical) | "disease progression"[MeSH Terms] OR ("disease"[All Fields] AND "progression"[All Fields]) OR "disease progression"[All Fields] OR ("disease progression"[MeSH Terms] OR ("disease"[All Fields] AND "progression"[All Fields]) OR "disease progression"[All Fields] OR ("progression"[All Fields] AND "disease"[All Fields]) OR "progression disease"[All Fields]) OR ("disease progression"[MeSH Terms] OR ("disease"[All Fields] AND "progression"[All Fields]) OR "disease progression"[All Fields] OR ("disease"[All Fields] AND "exacerbation"[All Fields]) OR "disease exacerbation"[All Fields]) OR ("disease progression"[MeSH Terms] OR ("disease"[All Fields] AND "progression"[All Fields]) OR "disease progression"[All Fields] OR ("exacerbation"[All Fields] AND "disease"[All Fields]) OR "exacerbation disease"[All Fields]) OR ("disease progression"[MeSH Terms] OR ("disease"[All Fields] AND "progression"[All Fields]) OR "disease progression"[All Fields] OR ("clinical"[All Fields] AND "course"[All Fields]) OR "clinical course"[All Fields]) OR ("disease progression"[MeSH Terms] OR ("disease"[All Fields] AND "progression"[All Fields]) OR "disease progression"[All Fields] OR ("clinical"[All Fields] AND "progression"[All Fields]) OR "clinical progression"[All Fields]) OR ("disease progression"[MeSH Terms] OR ("disease"[All Fields] AND "progression"[All Fields]) OR "disease progression"[All Fields] OR ("progression"[All Fields] AND "clinical"[All Fields]) OR "progression clinical"[All Fields]) | 978,396 |
| 25 | "Disease Progression"[Mesh] | "Disease Progression"[MeSH Terms] | 224,200 |
| 24 | ((((((((((((((((((((((((((((((exacerbation) OR (Symptom Flare Up)) OR (Flare Ups, Symptom)) OR (Flare Up, Symptom)) OR (Symptom Flare Ups)) OR (Symptom Flareup)) OR (Flareups, Symptom)) OR (Flareup, Symptom)) OR (Symptom Flareups)) OR (Symptom Flare-up)) OR (Flare-ups, Symptom)) OR (Flare-up, Symptom)) OR (Symptom Flare-ups)) OR (Acute Symptom Flare)) OR (Acute Symptom Flares)) OR (Symptom Flare, Acute)) OR (Symptom Flaring Up)) OR (Flaring Up, Symptom)) OR (Symptom Exacerbation)) OR (Exacerbation, Symptom)) OR (Symptom Exacerbations)) OR (Symptom Worsening)) OR (Worsening, Symptom)) OR (Symptom Exaggeration)) OR (Exaggeration, Symptom)) OR (Symptom Exaggerations)) OR (Symptom Magnification)) OR (Magnification, Symptom)) OR (Symptom Magnifications)) OR (Symptom Increase)) OR (Increase, Symptom) | "exacerbate"[All Fields] OR "exacerbated"[All Fields] OR "exacerbates"[All Fields] OR "exacerbating"[All Fields] OR "exacerbation"[All Fields] OR "exacerbations"[All Fields] OR "exacerbator"[All Fields] OR "exacerbators"[All Fields] OR ("symptom flare up"[MeSH Terms] OR ("symptom"[All Fields] AND "flare"[All Fields] AND "up"[All Fields]) OR "symptom flare up"[All Fields]) OR ("symptom flare up"[MeSH Terms] OR ("symptom"[All Fields] AND "flare"[All Fields] AND "up"[All Fields]) OR "symptom flare up"[All Fields] OR ("flare"[All Fields] AND "ups"[All Fields] AND "symptom"[All Fields])) OR ("symptom flare up"[MeSH Terms] OR ("symptom"[All Fields] AND "flare"[All Fields] AND "up"[All Fields]) OR "symptom flare up"[All Fields] OR ("flare"[All Fields] AND "up"[All Fields] AND "symptom"[All Fields]) OR "flare up symptom"[All Fields]) OR ("symptom flare up"[MeSH Terms] OR ("symptom"[All Fields] AND "flare"[All Fields] AND "up"[All Fields]) OR "symptom flare up"[All Fields] OR ("symptom"[All Fields] AND "flare"[All Fields] AND "ups"[All Fields]) OR "symptom flare ups"[All Fields]) OR ("symptom flare up"[MeSH Terms] OR ("symptom"[All Fields] AND "flare"[All Fields] AND "up"[All Fields]) OR "symptom flare up"[All Fields] OR ("symptom"[All Fields] AND "flareup"[All Fields])) OR ("symptom flare up"[MeSH Terms] OR ("symptom"[All Fields] AND "flare"[All Fields] AND "up"[All Fields]) OR "symptom flare up"[All Fields] OR ("flareups"[All Fields] AND "symptom"[All Fields])) OR ("symptom flare up"[MeSH Terms] OR ("symptom"[All Fields] AND "flare"[All Fields] AND "up"[All Fields]) OR "symptom flare up"[All Fields] OR ("flareup"[All Fields] AND "symptom"[All Fields])) OR ("symptom flare up"[MeSH Terms] OR ("symptom"[All Fields] AND "flare"[All Fields] AND "up"[All Fields]) OR "symptom flare up"[All Fields] OR ("symptom"[All Fields] AND "flareups"[All Fields])) OR ("symptom flare up"[MeSH Terms] OR ("symptom"[All Fields] AND "flare"[All Fields] AND "up"[All Fields]) OR "symptom flare up"[All Fields]) OR ("symptom flare up"[MeSH Terms] OR ("symptom"[All Fields] AND "flare"[All Fields] AND "up"[All Fields]) OR "symptom flare up"[All Fields] OR ("flare"[All Fields] AND "ups"[All Fields] AND "symptom"[All Fields])) OR ("symptom flare up"[MeSH Terms] OR ("symptom"[All Fields] AND "flare"[All Fields] AND "up"[All Fields]) OR "symptom flare up"[All Fields] OR ("flare"[All Fields] AND "up"[All Fields] AND "symptom"[All Fields]) OR "flare up symptom"[All Fields]) OR ("symptom flare up"[MeSH Terms] OR ("symptom"[All Fields] AND "flare"[All Fields] AND "up"[All Fields]) OR "symptom flare up"[All Fields] OR ("symptom"[All Fields] AND "flare"[All Fields] AND "ups"[All Fields]) OR "symptom flare ups"[All Fields]) OR ("symptom flare up"[MeSH Terms] OR ("symptom"[All Fields] AND "flare"[All Fields] AND "up"[All Fields]) OR "symptom flare up"[All Fields] OR ("acute"[All Fields] AND "symptom"[All Fields] AND "flare"[All Fields]) OR "acute symptom flare"[All Fields]) OR ("symptom flare up"[MeSH Terms] OR ("symptom"[All Fields] AND "flare"[All Fields] AND "up"[All Fields]) OR "symptom flare up"[All Fields] OR ("acute"[All Fields] AND "symptom"[All Fields] AND "flares"[All Fields]) OR "acute symptom flares"[All Fields]) OR ("symptom flare up"[MeSH Terms] OR ("symptom"[All Fields] AND "flare"[All Fields] AND "up"[All Fields]) OR "symptom flare up"[All Fields] OR ("symptom"[All Fields] AND "flare"[All Fields] AND "acute"[All Fields])) OR ("symptom flare up"[MeSH Terms] OR ("symptom"[All Fields] AND "flare"[All Fields] AND "up"[All Fields]) OR "symptom flare up"[All Fields] OR ("symptom"[All Fields] AND "flaring"[All Fields] AND "up"[All Fields])) OR ("symptom flare up"[MeSH Terms] OR ("symptom"[All Fields] AND "flare"[All Fields] AND "up"[All Fields]) OR "symptom flare up"[All Fields] OR ("flaring"[All Fields] AND "up"[All Fields] AND "symptom"[All Fields])) OR ("symptom flare up"[MeSH Terms] OR ("symptom"[All Fields] AND "flare"[All Fields] AND "up"[All Fields]) OR "symptom flare up"[All Fields] OR ("symptom"[All Fields] AND "exacerbation"[All Fields]) OR "symptom exacerbation"[All Fields]) OR ("symptom flare up"[MeSH Terms] OR ("symptom"[All Fields] AND "flare"[All Fields] AND "up"[All Fields]) OR "symptom flare up"[All Fields] OR ("exacerbation"[All Fields] AND "symptom"[All Fields]) OR "exacerbation symptom"[All Fields]) OR ("symptom flare up"[MeSH Terms] OR ("symptom"[All Fields] AND "flare"[All Fields] AND "up"[All Fields]) OR "symptom flare up"[All Fields] OR ("symptom"[All Fields] AND "exacerbations"[All Fields]) OR "symptom exacerbations"[All Fields]) OR ("symptom flare up"[MeSH Terms] OR ("symptom"[All Fields] AND "flare"[All Fields] AND "up"[All Fields]) OR "symptom flare up"[All Fields] OR ("symptom"[All Fields] AND "worsening"[All Fields]) OR "symptom worsening"[All Fields]) OR ("symptom flare up"[MeSH Terms] OR ("symptom"[All Fields] AND "flare"[All Fields] AND "up"[All Fields]) OR "symptom flare up"[All Fields] OR ("worsening"[All Fields] AND "symptom"[All Fields]) OR "worsening symptom"[All Fields]) OR ("symptom flare up"[MeSH Terms] OR ("symptom"[All Fields] AND "flare"[All Fields] AND "up"[All Fields]) OR "symptom flare up"[All Fields] OR ("symptom"[All Fields] AND "exaggeration"[All Fields]) OR "symptom exaggeration"[All Fields]) OR ("symptom flare up"[MeSH Terms] OR ("symptom"[All Fields] AND "flare"[All Fields] AND "up"[All Fields]) OR "symptom flare up"[All Fields] OR ("exaggeration"[All Fields] AND "symptom"[All Fields]) OR "exaggeration symptom"[All Fields]) OR ("symptom flare up"[MeSH Terms] OR ("symptom"[All Fields] AND "flare"[All Fields] AND "up"[All Fields]) OR "symptom flare up"[All Fields] OR ("symptom"[All Fields] AND "exaggerations"[All Fields])) OR ("symptom flare up"[MeSH Terms] OR ("symptom"[All Fields] AND "flare"[All Fields] AND "up"[All Fields]) OR "symptom flare up"[All Fields] OR ("symptom"[All Fields] AND "magnification"[All Fields]) OR "symptom magnification"[All Fields]) OR ("symptom flare up"[MeSH Terms] OR ("symptom"[All Fields] AND "flare"[All Fields] AND "up"[All Fields]) OR "symptom flare up"[All Fields] OR ("magnification"[All Fields] AND "symptom"[All Fields]) OR "magnification symptom"[All Fields]) OR ("symptom flare up"[MeSH Terms] OR ("symptom"[All Fields] AND "flare"[All Fields] AND "up"[All Fields]) OR "symptom flare up"[All Fields] OR ("symptom"[All Fields] AND "magnifications"[All Fields])) OR ("symptom flare up"[MeSH Terms] OR ("symptom"[All Fields] AND "flare"[All Fields] AND "up"[All Fields]) OR "symptom flare up"[All Fields] OR ("symptom"[All Fields] AND "increase"[All Fields]) OR "symptom increase"[All Fields]) OR ("symptom flare up"[MeSH Terms] OR ("symptom"[All Fields] AND "flare"[All Fields] AND "up"[All Fields]) OR "symptom flare up"[All Fields] OR ("increase"[All Fields] AND "symptom"[All Fields]) OR "increase symptom"[All Fields]) | 208,282 |
| 23 | "Symptom Flare Up"[Mesh] | "Symptom Flare Up"[MeSH Terms] | 1,461 |
| 22 | ("Lung Diseases, Interstitial"[Mesh]) OR ((((((((((((((interstitial lung disease) OR (Diffuse Parenchymal Lung Diseases)) OR (Interstitial Lung Diseases)) OR (Interstitial Lung Disease)) OR (Lung Disease, Interstitial)) OR (Diffuse Parenchymal Lung Disease)) OR (Pneumonia, Interstitial)) OR (Interstitial Pneumonia)) OR (Interstitial Pneumonias)) OR (Pneumonias, Interstitial)) OR (Pneumonitis, Interstitial)) OR (Interstitial Pneumonitides)) OR (Interstitial Pneumonitis)) OR (Pneumonitides, Interstitial)) | "lung diseases, interstitial"[MeSH Terms] OR ("lung diseases, interstitial"[MeSH Terms] OR ("lung"[All Fields] AND "diseases"[All Fields] AND "interstitial"[All Fields]) OR "interstitial lung diseases"[All Fields] OR ("interstitial"[All Fields] AND "lung"[All Fields] AND "disease"[All Fields]) OR "interstitial lung disease"[All Fields] OR ("lung diseases, interstitial"[MeSH Terms] OR ("lung"[All Fields] AND "diseases"[All Fields] AND "interstitial"[All Fields]) OR "interstitial lung diseases"[All Fields] OR ("diffuse"[All Fields] AND "parenchymal"[All Fields] AND "lung"[All Fields] AND "diseases"[All Fields]) OR "diffuse parenchymal lung diseases"[All Fields]) OR ("lung diseases, interstitial"[MeSH Terms] OR ("lung"[All Fields] AND "diseases"[All Fields] AND "interstitial"[All Fields]) OR "interstitial lung diseases"[All Fields] OR ("interstitial"[All Fields] AND "lung"[All Fields] AND "diseases"[All Fields])) OR ("lung diseases, interstitial"[MeSH Terms] OR ("lung"[All Fields] AND "diseases"[All Fields] AND "interstitial"[All Fields]) OR "interstitial lung diseases"[All Fields] OR ("interstitial"[All Fields] AND "lung"[All Fields] AND "disease"[All Fields]) OR "interstitial lung disease"[All Fields]) OR ("lung diseases, interstitial"[MeSH Terms] OR ("lung"[All Fields] AND "diseases"[All Fields] AND "interstitial"[All Fields]) OR "interstitial lung diseases"[All Fields] OR ("lung"[All Fields] AND "disease"[All Fields] AND "interstitial"[All Fields]) OR "lung disease interstitial"[All Fields]) OR ("lung diseases, interstitial"[MeSH Terms] OR ("lung"[All Fields] AND "diseases"[All Fields] AND "interstitial"[All Fields]) OR "interstitial lung diseases"[All Fields] OR ("diffuse"[All Fields] AND "parenchymal"[All Fields] AND "lung"[All Fields] AND "disease"[All Fields]) OR "diffuse parenchymal lung disease"[All Fields]) OR ("lung diseases, interstitial"[MeSH Terms] OR ("lung"[All Fields] AND "diseases"[All Fields] AND "interstitial"[All Fields]) OR "interstitial lung diseases"[All Fields] OR ("pneumonia"[All Fields] AND "interstitial"[All Fields]) OR "pneumonia interstitial"[All Fields]) OR ("lung diseases, interstitial"[MeSH Terms] OR ("lung"[All Fields] AND "diseases"[All Fields] AND "interstitial"[All Fields]) OR "interstitial lung diseases"[All Fields] OR ("interstitial"[All Fields] AND "pneumonia"[All Fields]) OR "interstitial pneumonia"[All Fields]) OR ("lung diseases, interstitial"[MeSH Terms] OR ("lung"[All Fields] AND "diseases"[All Fields] AND "interstitial"[All Fields]) OR "interstitial lung diseases"[All Fields] OR ("interstitial"[All Fields] AND "pneumonias"[All Fields]) OR "interstitial pneumonias"[All Fields]) OR ("lung diseases, interstitial"[MeSH Terms] OR ("lung"[All Fields] AND "diseases"[All Fields] AND "interstitial"[All Fields]) OR "interstitial lung diseases"[All Fields] OR ("pneumonias"[All Fields] AND "interstitial"[All Fields]) OR "pneumonias interstitial"[All Fields]) OR ("lung diseases, interstitial"[MeSH Terms] OR ("lung"[All Fields] AND "diseases"[All Fields] AND "interstitial"[All Fields]) OR "interstitial lung diseases"[All Fields] OR ("pneumonitis"[All Fields] AND "interstitial"[All Fields]) OR "pneumonitis interstitial"[All Fields]) OR ("lung diseases, interstitial"[MeSH Terms] OR ("lung"[All Fields] AND "diseases"[All Fields] AND "interstitial"[All Fields]) OR "interstitial lung diseases"[All Fields] OR ("interstitial"[All Fields] AND "pneumonitides"[All Fields]) OR "interstitial pneumonitides"[All Fields]) OR ("lung diseases, interstitial"[MeSH Terms] OR ("lung"[All Fields] AND "diseases"[All Fields] AND "interstitial"[All Fields]) OR "interstitial lung diseases"[All Fields] OR ("interstitial"[All Fields] AND "pneumonitis"[All Fields]) OR "interstitial pneumonitis"[All Fields]) OR ("lung diseases, interstitial"[MeSH Terms] OR ("lung"[All Fields] AND "diseases"[All Fields] AND "interstitial"[All Fields]) OR "interstitial lung diseases"[All Fields] OR ("pneumonitides"[All Fields] AND "interstitial"[All Fields]))) | 111,870 |
| 21 | (((((((((((((interstitial lung disease) OR (Diffuse Parenchymal Lung Diseases)) OR (Interstitial Lung Diseases)) OR (Interstitial Lung Disease)) OR (Lung Disease, Interstitial)) OR (Diffuse Parenchymal Lung Disease)) OR (Pneumonia, Interstitial)) OR (Interstitial Pneumonia)) OR (Interstitial Pneumonias)) OR (Pneumonias, Interstitial)) OR (Pneumonitis, Interstitial)) OR (Interstitial Pneumonitides)) OR (Interstitial Pneumonitis)) OR (Pneumonitides, Interstitial) | "lung diseases, interstitial"[MeSH Terms] OR ("lung"[All Fields] AND "diseases"[All Fields] AND "interstitial"[All Fields]) OR "interstitial lung diseases"[All Fields] OR ("interstitial"[All Fields] AND "lung"[All Fields] AND "disease"[All Fields]) OR "interstitial lung disease"[All Fields] OR ("lung diseases, interstitial"[MeSH Terms] OR ("lung"[All Fields] AND "diseases"[All Fields] AND "interstitial"[All Fields]) OR "interstitial lung diseases"[All Fields] OR ("diffuse"[All Fields] AND "parenchymal"[All Fields] AND "lung"[All Fields] AND "diseases"[All Fields]) OR "diffuse parenchymal lung diseases"[All Fields]) OR ("lung diseases, interstitial"[MeSH Terms] OR ("lung"[All Fields] AND "diseases"[All Fields] AND "interstitial"[All Fields]) OR "interstitial lung diseases"[All Fields] OR ("interstitial"[All Fields] AND "lung"[All Fields] AND "diseases"[All Fields])) OR ("lung diseases, interstitial"[MeSH Terms] OR ("lung"[All Fields] AND "diseases"[All Fields] AND "interstitial"[All Fields]) OR "interstitial lung diseases"[All Fields] OR ("interstitial"[All Fields] AND "lung"[All Fields] AND "disease"[All Fields]) OR "interstitial lung disease"[All Fields]) OR ("lung diseases, interstitial"[MeSH Terms] OR ("lung"[All Fields] AND "diseases"[All Fields] AND "interstitial"[All Fields]) OR "interstitial lung diseases"[All Fields] OR ("lung"[All Fields] AND "disease"[All Fields] AND "interstitial"[All Fields]) OR "lung disease interstitial"[All Fields]) OR ("lung diseases, interstitial"[MeSH Terms] OR ("lung"[All Fields] AND "diseases"[All Fields] AND "interstitial"[All Fields]) OR "interstitial lung diseases"[All Fields] OR ("diffuse"[All Fields] AND "parenchymal"[All Fields] AND "lung"[All Fields] AND "disease"[All Fields]) OR "diffuse parenchymal lung disease"[All Fields]) OR ("lung diseases, interstitial"[MeSH Terms] OR ("lung"[All Fields] AND "diseases"[All Fields] AND "interstitial"[All Fields]) OR "interstitial lung diseases"[All Fields] OR ("pneumonia"[All Fields] AND "interstitial"[All Fields]) OR "pneumonia interstitial"[All Fields]) OR ("lung diseases, interstitial"[MeSH Terms] OR ("lung"[All Fields] AND "diseases"[All Fields] AND "interstitial"[All Fields]) OR "interstitial lung diseases"[All Fields] OR ("interstitial"[All Fields] AND "pneumonia"[All Fields]) OR "interstitial pneumonia"[All Fields]) OR ("lung diseases, interstitial"[MeSH Terms] OR ("lung"[All Fields] AND "diseases"[All Fields] AND "interstitial"[All Fields]) OR "interstitial lung diseases"[All Fields] OR ("interstitial"[All Fields] AND "pneumonias"[All Fields]) OR "interstitial pneumonias"[All Fields]) OR ("lung diseases, interstitial"[MeSH Terms] OR ("lung"[All Fields] AND "diseases"[All Fields] AND "interstitial"[All Fields]) OR "interstitial lung diseases"[All Fields] OR ("pneumonias"[All Fields] AND "interstitial"[All Fields]) OR "pneumonias interstitial"[All Fields]) OR ("lung diseases, interstitial"[MeSH Terms] OR ("lung"[All Fields] AND "diseases"[All Fields] AND "interstitial"[All Fields]) OR "interstitial lung diseases"[All Fields] OR ("pneumonitis"[All Fields] AND "interstitial"[All Fields]) OR "pneumonitis interstitial"[All Fields]) OR ("lung diseases, interstitial"[MeSH Terms] OR ("lung"[All Fields] AND "diseases"[All Fields] AND "interstitial"[All Fields]) OR "interstitial lung diseases"[All Fields] OR ("interstitial"[All Fields] AND "pneumonitides"[All Fields]) OR "interstitial pneumonitides"[All Fields]) OR ("lung diseases, interstitial"[MeSH Terms] OR ("lung"[All Fields] AND "diseases"[All Fields] AND "interstitial"[All Fields]) OR "interstitial lung diseases"[All Fields] OR ("interstitial"[All Fields] AND "pneumonitis"[All Fields]) OR "interstitial pneumonitis"[All Fields]) OR ("lung diseases, interstitial"[MeSH Terms] OR ("lung"[All Fields] AND "diseases"[All Fields] AND "interstitial"[All Fields]) OR "interstitial lung diseases"[All Fields] OR ("pneumonitides"[All Fields] AND "interstitial"[All Fields])) | 111,870 |
| 20 | "Lung Diseases, Interstitial"[Mesh] | "lung diseases, interstitial"[MeSH Terms] | 91,061 |
| 19 | ((((((((((((((((("Arthritis, Rheumatoid"[Mesh]) OR (((rheumatoid arthritis) OR (Rheumatoid Arthritis)) OR (Arthritis, Rheumatoid))) OR ("Scleroderma, Systemic"[Mesh])) OR (((((scleroderma) OR (Sclerosis, Systemic)) OR (Systemic Scleroderma)) OR (Systemic Sclerosis)) OR (Scleroderma, Systemic))) OR ("Myositis"[Mesh])) OR ((((((((((((((((((((((((((((((antisynthetase syndrome) OR (myositis)) OR (Myositides)) OR (Inflammatory Muscle Diseases)) OR (Inflammatory Muscle Disease)) OR (Muscle Disease, Inflammatory)) OR (Inflammatory Myopathy)) OR (Inflammatory Myopathies)) OR (Myopathies, Inflammatory)) OR (Muscle Diseases, Inflammatory)) OR (Myopathy, Inflammatory)) OR (Myositis, Focal)) OR (Focal Myositides)) OR (Focal Myositis)) OR (Myositides, Focal)) OR (Myositis, Proliferative)) OR (Myositides, Proliferative)) OR (Proliferative Myositides)) OR (Proliferative Myositis)) OR (Myositis, Infectious)) OR (Infectious Myositides)) OR (Myositides, Infectious)) OR (Infectious Myositis)) OR (Idiopathic Inflammatory Myopathies)) OR (Myopathy, Idiopathic Inflammatory)) OR (Inflammatory Myopathies, Idiopathic)) OR (Myopathies, Idiopathic Inflammatory)) OR (Idiopathic Inflammatory Myopathy)) OR (Idiopathic Inflammatory Myositis)) OR (Inflammatory Myopathy, Idiopathic))) OR ("Polymyositis"[Mesh])) OR (((((((((((polymyositis) OR (Polymyositides)) OR (Myositis, Multiple)) OR (Multiple Myositis)) OR (Myositides, Multiple)) OR (Polymyositis Ossificans)) OR (Ossificans, Polymyositis)) OR (Polymyositis, Idiopathic)) OR (Idiopathic Polymyositides)) OR (Idiopathic Polymyositis)) OR (Polymyositides, Idiopathic))) OR ("Dermatomyositis"[Mesh])) OR ((((((((((((dermatomyositis) OR (Polymyositis-Dermatomyositis)) OR (Polymyositis Dermatomyositis)) OR (Dermatopolymyositis)) OR (Dermatomyositis, Adult Type)) OR (Adult Type Dermatomyositis)) OR (Dermatomyositis, Childhood Type)) OR (Childhood Type Dermatomyositis)) OR (Juvenile Dermatomyositis)) OR (Dermatomyositis, Juvenile)) OR (Juvenile Myositis)) OR (Myositis, Juvenile))) OR ("Sjogren's Syndrome"[Mesh])) OR (((((((sjogren syndrome) OR (Sjögren syndrome)) OR (Sjogrens Syndrome)) OR (Syndrome, Sjogren's)) OR (Sjogren Syndrome)) OR (Sicca Syndrome)) OR (Syndrome, Sicca))) OR ("Mixed Connective Tissue Disease"[Mesh])) OR (((((mixed connective tissue disease) OR (MCTD)) OR (Connective Tissue Disease, Mixed)) OR (Sharp Syndrome)) OR (Syndrome, Sharp))) OR ("Lupus Erythematosus, Systemic"[Mesh])) OR ((((((systemic lupus erythematosus) OR (Lupus Erythematosus Disseminatus)) OR (Systemic Lupus Erythematosus)) OR (Libman-Sacks Disease)) OR (Disease, Libman-Sacks)) OR (Libman Sacks Disease))) OR ("Connective Tissue Diseases"[Mesh])) OR ((((connective tissue disease) OR (Connective Tissue Disease)) OR (Disease, Connective Tissue)) OR (Diseases, Connective Tissue)) | "arthritis, rheumatoid"[MeSH Terms] OR ("arthritis, rheumatoid"[MeSH Terms] OR ("arthritis"[All Fields] AND "rheumatoid"[All Fields]) OR "rheumatoid arthritis"[All Fields] OR ("rheumatoid"[All Fields] AND "arthritis"[All Fields]) OR ("arthritis, rheumatoid"[MeSH Terms] OR ("arthritis"[All Fields] AND "rheumatoid"[All Fields]) OR "rheumatoid arthritis"[All Fields] OR ("rheumatoid"[All Fields] AND "arthritis"[All Fields])) OR ("arthritis, rheumatoid"[MeSH Terms] OR ("arthritis"[All Fields] AND "rheumatoid"[All Fields]) OR "rheumatoid arthritis"[All Fields] OR "arthritis rheumatoid"[All Fields])) OR "scleroderma, systemic"[MeSH Terms] OR ("scleroderma, systemic"[MeSH Terms] OR ("scleroderma"[All Fields] AND "systemic"[All Fields]) OR "systemic scleroderma"[All Fields] OR "scleroderma"[All Fields] OR "scleroderma, localized"[MeSH Terms] OR ("scleroderma"[All Fields] AND "localized"[All Fields]) OR "localized scleroderma"[All Fields] OR "sclerodermas"[All Fields] OR ("scleroderma, systemic"[MeSH Terms] OR ("scleroderma"[All Fields] AND "systemic"[All Fields]) OR "systemic scleroderma"[All Fields] OR ("sclerosis"[All Fields] AND "systemic"[All Fields]) OR "sclerosis systemic"[All Fields]) OR ("scleroderma, systemic"[MeSH Terms] OR ("scleroderma"[All Fields] AND "systemic"[All Fields]) OR "systemic scleroderma"[All Fields] OR ("systemic"[All Fields] AND "scleroderma"[All Fields])) OR ("scleroderma, systemic"[MeSH Terms] OR ("scleroderma"[All Fields] AND "systemic"[All Fields]) OR "systemic scleroderma"[All Fields] OR ("systemic"[All Fields] AND "sclerosis"[All Fields]) OR "systemic sclerosis"[All Fields]) OR ("scleroderma, systemic"[MeSH Terms] OR ("scleroderma"[All Fields] AND "systemic"[All Fields]) OR "systemic scleroderma"[All Fields] OR "scleroderma systemic"[All Fields])) OR "Myositis"[MeSH Terms] OR ("antisynthetase syndrome"[Supplementary Concept] OR "antisynthetase syndrome"[All Fields] OR ("Myositis"[MeSH Terms] OR "Myositis"[All Fields] OR "myositides"[All Fields]) OR ("Myositis"[MeSH Terms] OR "Myositis"[All Fields] OR "myositides"[All Fields]) OR ("Myositis"[MeSH Terms] OR "Myositis"[All Fields] OR ("inflammatory"[All Fields] AND "muscle"[All Fields] AND "diseases"[All Fields]) OR "inflammatory muscle diseases"[All Fields]) OR ("Myositis"[MeSH Terms] OR "Myositis"[All Fields] OR ("inflammatory"[All Fields] AND "muscle"[All Fields] AND "disease"[All Fields]) OR "inflammatory muscle disease"[All Fields]) OR ("Myositis"[MeSH Terms] OR "Myositis"[All Fields] OR ("muscle"[All Fields] AND "disease"[All Fields] AND "inflammatory"[All Fields])) OR ("Myositis"[MeSH Terms] OR "Myositis"[All Fields] OR ("inflammatory"[All Fields] AND "myopathy"[All Fields]) OR "inflammatory myopathy"[All Fields]) OR ("Myositis"[MeSH Terms] OR "Myositis"[All Fields] OR ("inflammatory"[All Fields] AND "myopathies"[All Fields]) OR "inflammatory myopathies"[All Fields]) OR ("Myositis"[MeSH Terms] OR "Myositis"[All Fields] OR ("myopathies"[All Fields] AND "inflammatory"[All Fields]) OR "myopathies inflammatory"[All Fields]) OR ("Myositis"[MeSH Terms] OR "Myositis"[All Fields] OR ("muscle"[All Fields] AND "diseases"[All Fields] AND "inflammatory"[All Fields]) OR "muscle diseases inflammatory"[All Fields]) OR ("Myositis"[MeSH Terms] OR "Myositis"[All Fields] OR ("myopathy"[All Fields] AND "inflammatory"[All Fields]) OR "myopathy inflammatory"[All Fields]) OR ("Myositis"[MeSH Terms] OR "Myositis"[All Fields] OR ("Myositis"[All Fields] AND "focal"[All Fields]) OR "myositis focal"[All Fields]) OR ("Myositis"[MeSH Terms] OR "Myositis"[All Fields] OR ("focal"[All Fields] AND "myositides"[All Fields])) OR ("Myositis"[MeSH Terms] OR "Myositis"[All Fields] OR ("focal"[All Fields] AND "Myositis"[All Fields]) OR "focal myositis"[All Fields]) OR ("Myositis"[MeSH Terms] OR "Myositis"[All Fields] OR ("myositides"[All Fields] AND "focal"[All Fields])) OR ("Myositis"[MeSH Terms] OR "Myositis"[All Fields] OR ("Myositis"[All Fields] AND "proliferative"[All Fields])) OR ("Myositis"[MeSH Terms] OR "Myositis"[All Fields] OR ("myositides"[All Fields] AND "proliferative"[All Fields])) OR ("Myositis"[MeSH Terms] OR "Myositis"[All Fields] OR ("proliferative"[All Fields] AND "myositides"[All Fields])) OR ("Myositis"[MeSH Terms] OR "Myositis"[All Fields] OR ("proliferative"[All Fields] AND "Myositis"[All Fields]) OR "proliferative myositis"[All Fields]) OR ("Myositis"[MeSH Terms] OR "Myositis"[All Fields] OR ("Myositis"[All Fields] AND "infectious"[All Fields]) OR "myositis infectious"[All Fields]) OR ("Myositis"[MeSH Terms] OR "Myositis"[All Fields] OR ("infectious"[All Fields] AND "myositides"[All Fields]) OR "infectious myositides"[All Fields]) OR ("Myositis"[MeSH Terms] OR "Myositis"[All Fields] OR ("myositides"[All Fields] AND "infectious"[All Fields])) OR ("Myositis"[MeSH Terms] OR "Myositis"[All Fields] OR ("infectious"[All Fields] AND "Myositis"[All Fields]) OR "infectious myositis"[All Fields]) OR ("Myositis"[MeSH Terms] OR "Myositis"[All Fields] OR ("idiopathic"[All Fields] AND "inflammatory"[All Fields] AND "myopathies"[All Fields]) OR "idiopathic inflammatory myopathies"[All Fields]) OR ("Myositis"[MeSH Terms] OR "Myositis"[All Fields] OR ("myopathy"[All Fields] AND "idiopathic"[All Fields] AND "inflammatory"[All Fields]) OR "myopathy idiopathic inflammatory"[All Fields]) OR ("Myositis"[MeSH Terms] OR "Myositis"[All Fields] OR ("inflammatory"[All Fields] AND "myopathies"[All Fields] AND "idiopathic"[All Fields])) OR ("Myositis"[MeSH Terms] OR "Myositis"[All Fields] OR ("myopathies"[All Fields] AND "idiopathic"[All Fields] AND "inflammatory"[All Fields]) OR "myopathies idiopathic inflammatory"[All Fields]) OR ("Myositis"[MeSH Terms] OR "Myositis"[All Fields] OR ("idiopathic"[All Fields] AND "inflammatory"[All Fields] AND "myopathy"[All Fields]) OR "idiopathic inflammatory myopathy"[All Fields]) OR ("Myositis"[MeSH Terms] OR "Myositis"[All Fields] OR ("idiopathic"[All Fields] AND "inflammatory"[All Fields] AND "Myositis"[All Fields]) OR "idiopathic inflammatory myositis"[All Fields]) OR ("Myositis"[MeSH Terms] OR "Myositis"[All Fields] OR ("inflammatory"[All Fields] AND "myopathy"[All Fields] AND "idiopathic"[All Fields]))) OR "Polymyositis"[MeSH Terms] OR ("Polymyositis"[MeSH Terms] OR "Polymyositis"[All Fields] OR ("Polymyositis"[MeSH Terms] OR "Polymyositis"[All Fields] OR "polymyositides"[All Fields]) OR ("Polymyositis"[MeSH Terms] OR "Polymyositis"[All Fields] OR ("Myositis"[All Fields] AND "multiple"[All Fields]) OR "myositis multiple"[All Fields]) OR ("Polymyositis"[MeSH Terms] OR "Polymyositis"[All Fields] OR ("multiple"[All Fields] AND "Myositis"[All Fields]) OR "multiple myositis"[All Fields]) OR ("Polymyositis"[MeSH Terms] OR "Polymyositis"[All Fields] OR ("myositides"[All Fields] AND "multiple"[All Fields])) OR ("Polymyositis"[MeSH Terms] OR "Polymyositis"[All Fields] OR ("Polymyositis"[All Fields] AND "ossificans"[All Fields]) OR "polymyositis ossificans"[All Fields]) OR ("Polymyositis"[MeSH Terms] OR "Polymyositis"[All Fields] OR ("ossificans"[All Fields] AND "Polymyositis"[All Fields]) OR "ossificans polymyositis"[All Fields]) OR ("Polymyositis"[MeSH Terms] OR "Polymyositis"[All Fields] OR ("Polymyositis"[All Fields] AND "idiopathic"[All Fields]) OR "polymyositis idiopathic"[All Fields]) OR ("Polymyositis"[MeSH Terms] OR "Polymyositis"[All Fields] OR ("idiopathic"[All Fields] AND "polymyositides"[All Fields])) OR ("Polymyositis"[MeSH Terms] OR "Polymyositis"[All Fields] OR ("idiopathic"[All Fields] AND "Polymyositis"[All Fields]) OR "idiopathic polymyositis"[All Fields]) OR ("Polymyositis"[MeSH Terms] OR "Polymyositis"[All Fields] OR ("polymyositides"[All Fields] AND "idiopathic"[All Fields]))) OR "Dermatomyositis"[MeSH Terms] OR ("Dermatomyositis"[MeSH Terms] OR "Dermatomyositis"[All Fields] OR ("Dermatomyositis"[MeSH Terms] OR "Dermatomyositis"[All Fields] OR ("Polymyositis"[All Fields] AND "Dermatomyositis"[All Fields]) OR "polymyositis dermatomyositis"[All Fields]) OR ("Dermatomyositis"[MeSH Terms] OR "Dermatomyositis"[All Fields] OR ("Polymyositis"[All Fields] AND "Dermatomyositis"[All Fields]) OR "polymyositis dermatomyositis"[All Fields]) OR ("Dermatomyositis"[MeSH Terms] OR "Dermatomyositis"[All Fields] OR "dermatopolymyositis"[All Fields]) OR ("Dermatomyositis"[MeSH Terms] OR "Dermatomyositis"[All Fields] OR ("Dermatomyositis"[All Fields] AND "adult"[All Fields] AND "type"[All Fields])) OR ("Dermatomyositis"[MeSH Terms] OR "Dermatomyositis"[All Fields] OR ("adult"[All Fields] AND "type"[All Fields] AND "Dermatomyositis"[All Fields]) OR "adult type dermatomyositis"[All Fields]) OR ("Dermatomyositis"[MeSH Terms] OR "Dermatomyositis"[All Fields] OR ("Dermatomyositis"[All Fields] AND "childhood"[All Fields] AND "type"[All Fields]) OR "dermatomyositis childhood type"[All Fields]) OR ("Dermatomyositis"[MeSH Terms] OR "Dermatomyositis"[All Fields] OR ("childhood"[All Fields] AND "type"[All Fields] AND "Dermatomyositis"[All Fields]) OR "childhood type dermatomyositis"[All Fields]) OR ("Dermatomyositis"[MeSH Terms] OR "Dermatomyositis"[All Fields] OR ("juvenile"[All Fields] AND "Dermatomyositis"[All Fields]) OR "juvenile dermatomyositis"[All Fields]) OR ("Dermatomyositis"[MeSH Terms] OR "Dermatomyositis"[All Fields] OR ("Dermatomyositis"[All Fields] AND "juvenile"[All Fields]) OR "dermatomyositis juvenile"[All Fields]) OR ("Dermatomyositis"[MeSH Terms] OR "Dermatomyositis"[All Fields] OR ("juvenile"[All Fields] AND "Myositis"[All Fields]) OR "juvenile myositis"[All Fields]) OR ("Dermatomyositis"[MeSH Terms] OR "Dermatomyositis"[All Fields] OR ("Myositis"[All Fields] AND "juvenile"[All Fields]) OR "myositis juvenile"[All Fields])) OR "Sjogren's Syndrome"[MeSH Terms] OR ("Sjogren's Syndrome"[MeSH Terms] OR ("sjogren s"[All Fields] AND "syndrome"[All Fields]) OR "Sjogren's Syndrome"[All Fields] OR ("sjogren"[All Fields] AND "syndrome"[All Fields]) OR "sjogren syndrome"[All Fields] OR ("Sjogren's Syndrome"[MeSH Terms] OR ("sjogren s"[All Fields] AND "syndrome"[All Fields]) OR "Sjogren's Syndrome"[All Fields] OR ("sjogren"[All Fields] AND "syndrome"[All Fields]) OR "sjogren syndrome"[All Fields]) OR ("Sjogren's Syndrome"[MeSH Terms] OR ("sjogren s"[All Fields] AND "syndrome"[All Fields]) OR "Sjogren's Syndrome"[All Fields] OR ("sjogrens"[All Fields] AND "syndrome"[All Fields]) OR "sjogrens syndrome"[All Fields]) OR ("Sjogren's Syndrome"[MeSH Terms] OR ("sjogren s"[All Fields] AND "syndrome"[All Fields]) OR "Sjogren's Syndrome"[All Fields] OR ("syndrome"[All Fields] AND "sjogren s"[All Fields]) OR "syndrome sjogren s"[All Fields]) OR ("Sjogren's Syndrome"[MeSH Terms] OR ("sjogren s"[All Fields] AND "syndrome"[All Fields]) OR "Sjogren's Syndrome"[All Fields] OR ("sjogren"[All Fields] AND "syndrome"[All Fields]) OR "sjogren syndrome"[All Fields]) OR ("Sjogren's Syndrome"[MeSH Terms] OR ("sjogren s"[All Fields] AND "syndrome"[All Fields]) OR "Sjogren's Syndrome"[All Fields] OR ("sicca"[All Fields] AND "syndrome"[All Fields]) OR "sicca syndrome"[All Fields]) OR ("Sjogren's Syndrome"[MeSH Terms] OR ("sjogren s"[All Fields] AND "syndrome"[All Fields]) OR "Sjogren's Syndrome"[All Fields] OR ("syndrome"[All Fields] AND "sicca"[All Fields]) OR "syndrome sicca"[All Fields])) OR "Mixed Connective Tissue Disease"[MeSH Terms] OR ("Mixed Connective Tissue Disease"[MeSH Terms] OR ("mixed"[All Fields] AND "connective"[All Fields] AND "tissue"[All Fields] AND "disease"[All Fields]) OR "Mixed Connective Tissue Disease"[All Fields] OR ("Mixed Connective Tissue Disease"[MeSH Terms] OR ("mixed"[All Fields] AND "connective"[All Fields] AND "tissue"[All Fields] AND "disease"[All Fields]) OR "Mixed Connective Tissue Disease"[All Fields] OR "mctd"[All Fields]) OR ("Mixed Connective Tissue Disease"[MeSH Terms] OR ("mixed"[All Fields] AND "connective"[All Fields] AND "tissue"[All Fields] AND "disease"[All Fields]) OR "Mixed Connective Tissue Disease"[All Fields] OR ("connective"[All Fields] AND "tissue"[All Fields] AND "disease"[All Fields] AND "mixed"[All Fields]) OR "connective tissue disease mixed"[All Fields]) OR ("Mixed Connective Tissue Disease"[MeSH Terms] OR ("mixed"[All Fields] AND "connective"[All Fields] AND "tissue"[All Fields] AND "disease"[All Fields]) OR "Mixed Connective Tissue Disease"[All Fields] OR ("sharp"[All Fields] AND "syndrome"[All Fields]) OR "sharp syndrome"[All Fields]) OR ("Mixed Connective Tissue Disease"[MeSH Terms] OR ("mixed"[All Fields] AND "connective"[All Fields] AND "tissue"[All Fields] AND "disease"[All Fields]) OR "Mixed Connective Tissue Disease"[All Fields] OR ("syndrome"[All Fields] AND "sharp"[All Fields]) OR "syndrome sharp"[All Fields])) OR "lupus erythematosus, systemic"[MeSH Terms] OR ("lupus erythematosus, systemic"[MeSH Terms] OR ("lupus"[All Fields] AND "erythematosus"[All Fields] AND "systemic"[All Fields]) OR "systemic lupus erythematosus"[All Fields] OR ("systemic"[All Fields] AND "lupus"[All Fields] AND "erythematosus"[All Fields]) OR ("lupus erythematosus, systemic"[MeSH Terms] OR ("lupus"[All Fields] AND "erythematosus"[All Fields] AND "systemic"[All Fields]) OR "systemic lupus erythematosus"[All Fields] OR ("lupus"[All Fields] AND "erythematosus"[All Fields] AND "disseminatus"[All Fields]) OR "lupus erythematosus disseminatus"[All Fields]) OR ("lupus erythematosus, systemic"[MeSH Terms] OR ("lupus"[All Fields] AND "erythematosus"[All Fields] AND "systemic"[All Fields]) OR "systemic lupus erythematosus"[All Fields] OR ("systemic"[All Fields] AND "lupus"[All Fields] AND "erythematosus"[All Fields])) OR ("lupus erythematosus, systemic"[MeSH Terms] OR ("lupus"[All Fields] AND "erythematosus"[All Fields] AND "systemic"[All Fields]) OR "systemic lupus erythematosus"[All Fields] OR ("libman"[All Fields] AND "sacks"[All Fields] AND "disease"[All Fields]) OR "libman sacks disease"[All Fields]) OR ("lupus erythematosus, systemic"[MeSH Terms] OR ("lupus"[All Fields] AND "erythematosus"[All Fields] AND "systemic"[All Fields]) OR "systemic lupus erythematosus"[All Fields] OR ("disease"[All Fields] AND "libman"[All Fields] AND "sacks"[All Fields]) OR "disease libman sacks"[All Fields]) OR ("lupus erythematosus, systemic"[MeSH Terms] OR ("lupus"[All Fields] AND "erythematosus"[All Fields] AND "systemic"[All Fields]) OR "systemic lupus erythematosus"[All Fields] OR ("libman"[All Fields] AND "sacks"[All Fields] AND "disease"[All Fields]) OR "libman sacks disease"[All Fields])) OR "Connective Tissue Diseases"[MeSH Terms] OR ("Connective Tissue Diseases"[MeSH Terms] OR ("connective"[All Fields] AND "tissue"[All Fields] AND "diseases"[All Fields]) OR "Connective Tissue Diseases"[All Fields] OR ("connective"[All Fields] AND "tissue"[All Fields] AND "disease"[All Fields]) OR "connective tissue disease"[All Fields] OR ("Connective Tissue Diseases"[MeSH Terms] OR ("connective"[All Fields] AND "tissue"[All Fields] AND "diseases"[All Fields]) OR "Connective Tissue Diseases"[All Fields] OR ("connective"[All Fields] AND "tissue"[All Fields] AND "disease"[All Fields]) OR "connective tissue disease"[All Fields]) OR ("Connective Tissue Diseases"[MeSH Terms] OR ("connective"[All Fields] AND "tissue"[All Fields] AND "diseases"[All Fields]) OR "Connective Tissue Diseases"[All Fields] OR ("disease"[All Fields] AND "connective"[All Fields] AND "tissue"[All Fields]) OR "disease connective tissue"[All Fields]) OR ("Connective Tissue Diseases"[MeSH Terms] OR ("connective"[All Fields] AND "tissue"[All Fields] AND "diseases"[All Fields]) OR "Connective Tissue Diseases"[All Fields] OR ("diseases"[All Fields] AND "connective"[All Fields] AND "tissue"[All Fields]) OR "diseases connective tissue"[All Fields])) | 499,979 |
| 18 | (((connective tissue disease) OR (Connective Tissue Disease)) OR (Disease, Connective Tissue)) OR (Diseases, Connective Tissue) | "connective tissue diseases"[MeSH Terms] OR ("connective"[All Fields] AND "tissue"[All Fields] AND "diseases"[All Fields]) OR "connective tissue diseases"[All Fields] OR ("connective"[All Fields] AND "tissue"[All Fields] AND "disease"[All Fields]) OR "connective tissue disease"[All Fields] OR ("connective tissue diseases"[MeSH Terms] OR ("connective"[All Fields] AND "tissue"[All Fields] AND "diseases"[All Fields]) OR "connective tissue diseases"[All Fields] OR ("connective"[All Fields] AND "tissue"[All Fields] AND "disease"[All Fields]) OR "connective tissue disease"[All Fields]) OR ("connective tissue diseases"[MeSH Terms] OR ("connective"[All Fields] AND "tissue"[All Fields] AND "diseases"[All Fields]) OR "connective tissue diseases"[All Fields] OR ("disease"[All Fields] AND "connective"[All Fields] AND "tissue"[All Fields]) OR "disease connective tissue"[All Fields]) OR ("connective tissue diseases"[MeSH Terms] OR ("connective"[All Fields] AND "tissue"[All Fields] AND "diseases"[All Fields]) OR "connective tissue diseases"[All Fields] OR ("diseases"[All Fields] AND "connective"[All Fields] AND "tissue"[All Fields]) OR "diseases connective tissue"[All Fields]) | 391,395 |
| 17 | "Connective Tissue Diseases"[Mesh] | "Connective Tissue Diseases"[MeSH Terms] | 367,300 |
| 16 | (((((systemic lupus erythematosus) OR (Lupus Erythematosus Disseminatus)) OR (Systemic Lupus Erythematosus)) OR (Libman-Sacks Disease)) OR (Disease, Libman-Sacks)) OR (Libman Sacks Disease) | "lupus erythematosus, systemic"[MeSH Terms] OR ("lupus"[All Fields] AND "erythematosus"[All Fields] AND "systemic"[All Fields]) OR "systemic lupus erythematosus"[All Fields] OR ("systemic"[All Fields] AND "lupus"[All Fields] AND "erythematosus"[All Fields]) OR ("lupus erythematosus, systemic"[MeSH Terms] OR ("lupus"[All Fields] AND "erythematosus"[All Fields] AND "systemic"[All Fields]) OR "systemic lupus erythematosus"[All Fields] OR ("lupus"[All Fields] AND "erythematosus"[All Fields] AND "disseminatus"[All Fields]) OR "lupus erythematosus disseminatus"[All Fields]) OR ("lupus erythematosus, systemic"[MeSH Terms] OR ("lupus"[All Fields] AND "erythematosus"[All Fields] AND "systemic"[All Fields]) OR "systemic lupus erythematosus"[All Fields] OR ("systemic"[All Fields] AND "lupus"[All Fields] AND "erythematosus"[All Fields])) OR ("lupus erythematosus, systemic"[MeSH Terms] OR ("lupus"[All Fields] AND "erythematosus"[All Fields] AND "systemic"[All Fields]) OR "systemic lupus erythematosus"[All Fields] OR ("libman"[All Fields] AND "sacks"[All Fields] AND "disease"[All Fields]) OR "libman sacks disease"[All Fields]) OR ("lupus erythematosus, systemic"[MeSH Terms] OR ("lupus"[All Fields] AND "erythematosus"[All Fields] AND "systemic"[All Fields]) OR "systemic lupus erythematosus"[All Fields] OR ("disease"[All Fields] AND "libman"[All Fields] AND "sacks"[All Fields]) OR "disease libman sacks"[All Fields]) OR ("lupus erythematosus, systemic"[MeSH Terms] OR ("lupus"[All Fields] AND "erythematosus"[All Fields] AND "systemic"[All Fields]) OR "systemic lupus erythematosus"[All Fields] OR ("libman"[All Fields] AND "sacks"[All Fields] AND "disease"[All Fields]) OR "libman sacks disease"[All Fields]) | 90,999 |
| 15 | "Lupus Erythematosus, Systemic"[Mesh] | "lupus erythematosus, systemic"[MeSH Terms] | 71,363 |
| 14 | ((((mixed connective tissue disease) OR (MCTD)) OR (Connective Tissue Disease, Mixed)) OR (Sharp Syndrome)) OR (Syndrome, Sharp) | "mixed connective tissue disease"[MeSH Terms] OR ("mixed"[All Fields] AND "connective"[All Fields] AND "tissue"[All Fields] AND "disease"[All Fields]) OR "mixed connective tissue disease"[All Fields] OR ("mixed connective tissue disease"[MeSH Terms] OR ("mixed"[All Fields] AND "connective"[All Fields] AND "tissue"[All Fields] AND "disease"[All Fields]) OR "mixed connective tissue disease"[All Fields] OR "mctd"[All Fields]) OR ("mixed connective tissue disease"[MeSH Terms] OR ("mixed"[All Fields] AND "connective"[All Fields] AND "tissue"[All Fields] AND "disease"[All Fields]) OR "mixed connective tissue disease"[All Fields] OR ("connective"[All Fields] AND "tissue"[All Fields] AND "disease"[All Fields] AND "mixed"[All Fields]) OR "connective tissue disease mixed"[All Fields]) OR ("mixed connective tissue disease"[MeSH Terms] OR ("mixed"[All Fields] AND "connective"[All Fields] AND "tissue"[All Fields] AND "disease"[All Fields]) OR "mixed connective tissue disease"[All Fields] OR ("sharp"[All Fields] AND "syndrome"[All Fields]) OR "sharp syndrome"[All Fields]) OR ("mixed connective tissue disease"[MeSH Terms] OR ("mixed"[All Fields] AND "connective"[All Fields] AND "tissue"[All Fields] AND "disease"[All Fields]) OR "mixed connective tissue disease"[All Fields] OR ("syndrome"[All Fields] AND "sharp"[All Fields]) OR "syndrome sharp"[All Fields]) | 7,129 |
| 13 | "Mixed Connective Tissue Disease"[Mesh] | "Mixed Connective Tissue Disease"[MeSH Terms] | 1,772 |
| 12 | ((((((sjogren syndrome) OR (Sjögren syndrome)) OR (Sjogrens Syndrome)) OR (Syndrome, Sjogren's)) OR (Sjogren Syndrome)) OR (Sicca Syndrome)) OR (Syndrome, Sicca) | "sjogren s syndrome"[MeSH Terms] OR ("sjogren s"[All Fields] AND "syndrome"[All Fields]) OR "sjogren s syndrome"[All Fields] OR ("sjogren"[All Fields] AND "syndrome"[All Fields]) OR "sjogren syndrome"[All Fields] OR ("sjogren s syndrome"[MeSH Terms] OR ("sjogren s"[All Fields] AND "syndrome"[All Fields]) OR "sjogren s syndrome"[All Fields] OR ("sjogren"[All Fields] AND "syndrome"[All Fields]) OR "sjogren syndrome"[All Fields]) OR ("sjogren s syndrome"[MeSH Terms] OR ("sjogren s"[All Fields] AND "syndrome"[All Fields]) OR "sjogren s syndrome"[All Fields] OR ("sjogrens"[All Fields] AND "syndrome"[All Fields]) OR "sjogrens syndrome"[All Fields]) OR ("sjogren s syndrome"[MeSH Terms] OR ("sjogren s"[All Fields] AND "syndrome"[All Fields]) OR "sjogren s syndrome"[All Fields] OR ("syndrome"[All Fields] AND "sjogren s"[All Fields]) OR "syndrome sjogren s"[All Fields]) OR ("sjogren s syndrome"[MeSH Terms] OR ("sjogren s"[All Fields] AND "syndrome"[All Fields]) OR "sjogren s syndrome"[All Fields] OR ("sjogren"[All Fields] AND "syndrome"[All Fields]) OR "sjogren syndrome"[All Fields]) OR ("sjogren s syndrome"[MeSH Terms] OR ("sjogren s"[All Fields] AND "syndrome"[All Fields]) OR "sjogren s syndrome"[All Fields] OR ("sicca"[All Fields] AND "syndrome"[All Fields]) OR "sicca syndrome"[All Fields]) OR ("sjogren s syndrome"[MeSH Terms] OR ("sjogren s"[All Fields] AND "syndrome"[All Fields]) OR "sjogren s syndrome"[All Fields] OR ("syndrome"[All Fields] AND "sicca"[All Fields]) OR "syndrome sicca"[All Fields]) | 23,811 |
| 11 | "Sjogren's Syndrome"[Mesh] | "Sjogren's Syndrome"[MeSH Terms] | 15,763 |
| 10 | (((((((((((dermatomyositis) OR (Polymyositis-Dermatomyositis)) OR (Polymyositis Dermatomyositis)) OR (Dermatopolymyositis)) OR (Dermatomyositis, Adult Type)) OR (Adult Type Dermatomyositis)) OR (Dermatomyositis, Childhood Type)) OR (Childhood Type Dermatomyositis)) OR (Juvenile Dermatomyositis)) OR (Dermatomyositis, Juvenile)) OR (Juvenile Myositis)) OR (Myositis, Juvenile) | "dermatomyositis"[MeSH Terms] OR "dermatomyositis"[All Fields] OR ("dermatomyositis"[MeSH Terms] OR "dermatomyositis"[All Fields] OR ("polymyositis"[All Fields] AND "dermatomyositis"[All Fields]) OR "polymyositis dermatomyositis"[All Fields]) OR ("dermatomyositis"[MeSH Terms] OR "dermatomyositis"[All Fields] OR ("polymyositis"[All Fields] AND "dermatomyositis"[All Fields]) OR "polymyositis dermatomyositis"[All Fields]) OR ("dermatomyositis"[MeSH Terms] OR "dermatomyositis"[All Fields] OR "dermatopolymyositis"[All Fields]) OR ("dermatomyositis"[MeSH Terms] OR "dermatomyositis"[All Fields] OR ("dermatomyositis"[All Fields] AND "adult"[All Fields] AND "type"[All Fields])) OR ("dermatomyositis"[MeSH Terms] OR "dermatomyositis"[All Fields] OR ("adult"[All Fields] AND "type"[All Fields] AND "dermatomyositis"[All Fields]) OR "adult type dermatomyositis"[All Fields]) OR ("dermatomyositis"[MeSH Terms] OR "dermatomyositis"[All Fields] OR ("dermatomyositis"[All Fields] AND "childhood"[All Fields] AND "type"[All Fields]) OR "dermatomyositis childhood type"[All Fields]) OR ("dermatomyositis"[MeSH Terms] OR "dermatomyositis"[All Fields] OR ("childhood"[All Fields] AND "type"[All Fields] AND "dermatomyositis"[All Fields]) OR "childhood type dermatomyositis"[All Fields]) OR ("dermatomyositis"[MeSH Terms] OR "dermatomyositis"[All Fields] OR ("juvenile"[All Fields] AND "dermatomyositis"[All Fields]) OR "juvenile dermatomyositis"[All Fields]) OR ("dermatomyositis"[MeSH Terms] OR "dermatomyositis"[All Fields] OR ("dermatomyositis"[All Fields] AND "juvenile"[All Fields]) OR "dermatomyositis juvenile"[All Fields]) OR ("dermatomyositis"[MeSH Terms] OR "dermatomyositis"[All Fields] OR ("juvenile"[All Fields] AND "myositis"[All Fields]) OR "juvenile myositis"[All Fields]) OR ("dermatomyositis"[MeSH Terms] OR "dermatomyositis"[All Fields] OR ("myositis"[All Fields] AND "juvenile"[All Fields]) OR "myositis juvenile"[All Fields]) | 14,243 |
| 9 | "Dermatomyositis"[Mesh] | "Dermatomyositis"[MeSH Terms] | 9,687 |
| 7 | "Polymyositis"[Mesh] | "Polymyositis"[MeSH Terms] | 11,245 |
| 8 | ((((((((((polymyositis) OR (Polymyositides)) OR (Myositis, Multiple)) OR (Multiple Myositis)) OR (Myositides, Multiple)) OR (Polymyositis Ossificans)) OR (Ossificans, Polymyositis)) OR (Polymyositis, Idiopathic)) OR (Idiopathic Polymyositides)) OR (Idiopathic Polymyositis)) OR (Polymyositides, Idiopathic) | "polymyositis"[MeSH Terms] OR "polymyositis"[All Fields] OR ("polymyositis"[MeSH Terms] OR "polymyositis"[All Fields] OR "polymyositides"[All Fields]) OR ("polymyositis"[MeSH Terms] OR "polymyositis"[All Fields] OR ("myositis"[All Fields] AND "multiple"[All Fields]) OR "myositis multiple"[All Fields]) OR ("polymyositis"[MeSH Terms] OR "polymyositis"[All Fields] OR ("multiple"[All Fields] AND "myositis"[All Fields]) OR "multiple myositis"[All Fields]) OR ("polymyositis"[MeSH Terms] OR "polymyositis"[All Fields] OR ("myositides"[All Fields] AND "multiple"[All Fields])) OR ("polymyositis"[MeSH Terms] OR "polymyositis"[All Fields] OR ("polymyositis"[All Fields] AND "ossificans"[All Fields]) OR "polymyositis ossificans"[All Fields]) OR ("polymyositis"[MeSH Terms] OR "polymyositis"[All Fields] OR ("ossificans"[All Fields] AND "polymyositis"[All Fields]) OR "ossificans polymyositis"[All Fields]) OR ("polymyositis"[MeSH Terms] OR "polymyositis"[All Fields] OR ("polymyositis"[All Fields] AND "idiopathic"[All Fields]) OR "polymyositis idiopathic"[All Fields]) OR ("polymyositis"[MeSH Terms] OR "polymyositis"[All Fields] OR ("idiopathic"[All Fields] AND "polymyositides"[All Fields])) OR ("polymyositis"[MeSH Terms] OR "polymyositis"[All Fields] OR ("idiopathic"[All Fields] AND "polymyositis"[All Fields]) OR "idiopathic polymyositis"[All Fields]) OR ("polymyositis"[MeSH Terms] OR "polymyositis"[All Fields] OR ("polymyositides"[All Fields] AND "idiopathic"[All Fields])) | 15,779 |
| 6 | (((((((((((((((((((((((((((((antisynthetase syndrome) OR (myositis)) OR (Myositides)) OR (Inflammatory Muscle Diseases)) OR (Inflammatory Muscle Disease)) OR (Muscle Disease, Inflammatory)) OR (Inflammatory Myopathy)) OR (Inflammatory Myopathies)) OR (Myopathies, Inflammatory)) OR (Muscle Diseases, Inflammatory)) OR (Myopathy, Inflammatory)) OR (Myositis, Focal)) OR (Focal Myositides)) OR (Focal Myositis)) OR (Myositides, Focal)) OR (Myositis, Proliferative)) OR (Myositides, Proliferative)) OR (Proliferative Myositides)) OR (Proliferative Myositis)) OR (Myositis, Infectious)) OR (Infectious Myositides)) OR (Myositides, Infectious)) OR (Infectious Myositis)) OR (Idiopathic Inflammatory Myopathies)) OR (Myopathy, Idiopathic Inflammatory)) OR (Inflammatory Myopathies, Idiopathic)) OR (Myopathies, Idiopathic Inflammatory)) OR (Idiopathic Inflammatory Myopathy)) OR (Idiopathic Inflammatory Myositis)) OR (Inflammatory Myopathy, Idiopathic) | "antisynthetase syndrome"[Supplementary Concept] OR "antisynthetase syndrome"[All Fields] OR ("myositis"[MeSH Terms] OR "myositis"[All Fields] OR "myositides"[All Fields]) OR ("myositis"[MeSH Terms] OR "myositis"[All Fields] OR "myositides"[All Fields]) OR ("myositis"[MeSH Terms] OR "myositis"[All Fields] OR ("inflammatory"[All Fields] AND "muscle"[All Fields] AND "diseases"[All Fields]) OR "inflammatory muscle diseases"[All Fields]) OR ("myositis"[MeSH Terms] OR "myositis"[All Fields] OR ("inflammatory"[All Fields] AND "muscle"[All Fields] AND "disease"[All Fields]) OR "inflammatory muscle disease"[All Fields]) OR ("myositis"[MeSH Terms] OR "myositis"[All Fields] OR ("muscle"[All Fields] AND "disease"[All Fields] AND "inflammatory"[All Fields])) OR ("myositis"[MeSH Terms] OR "myositis"[All Fields] OR ("inflammatory"[All Fields] AND "myopathy"[All Fields]) OR "inflammatory myopathy"[All Fields]) OR ("myositis"[MeSH Terms] OR "myositis"[All Fields] OR ("inflammatory"[All Fields] AND "myopathies"[All Fields]) OR "inflammatory myopathies"[All Fields]) OR ("myositis"[MeSH Terms] OR "myositis"[All Fields] OR ("myopathies"[All Fields] AND "inflammatory"[All Fields]) OR "myopathies inflammatory"[All Fields]) OR ("myositis"[MeSH Terms] OR "myositis"[All Fields] OR ("muscle"[All Fields] AND "diseases"[All Fields] AND "inflammatory"[All Fields]) OR "muscle diseases inflammatory"[All Fields]) OR ("myositis"[MeSH Terms] OR "myositis"[All Fields] OR ("myopathy"[All Fields] AND "inflammatory"[All Fields]) OR "myopathy inflammatory"[All Fields]) OR ("myositis"[MeSH Terms] OR "myositis"[All Fields] OR ("myositis"[All Fields] AND "focal"[All Fields]) OR "myositis focal"[All Fields]) OR ("myositis"[MeSH Terms] OR "myositis"[All Fields] OR ("focal"[All Fields] AND "myositides"[All Fields])) OR ("myositis"[MeSH Terms] OR "myositis"[All Fields] OR ("focal"[All Fields] AND "myositis"[All Fields]) OR "focal myositis"[All Fields]) OR ("myositis"[MeSH Terms] OR "myositis"[All Fields] OR ("myositides"[All Fields] AND "focal"[All Fields])) OR ("myositis"[MeSH Terms] OR "myositis"[All Fields] OR ("myositis"[All Fields] AND "proliferative"[All Fields])) OR ("myositis"[MeSH Terms] OR "myositis"[All Fields] OR ("myositides"[All Fields] AND "proliferative"[All Fields])) OR ("myositis"[MeSH Terms] OR "myositis"[All Fields] OR ("proliferative"[All Fields] AND "myositides"[All Fields])) OR ("myositis"[MeSH Terms] OR "myositis"[All Fields] OR ("proliferative"[All Fields] AND "myositis"[All Fields]) OR "proliferative myositis"[All Fields]) OR ("myositis"[MeSH Terms] OR "myositis"[All Fields] OR ("myositis"[All Fields] AND "infectious"[All Fields]) OR "myositis infectious"[All Fields]) OR ("myositis"[MeSH Terms] OR "myositis"[All Fields] OR ("infectious"[All Fields] AND "myositides"[All Fields]) OR "infectious myositides"[All Fields]) OR ("myositis"[MeSH Terms] OR "myositis"[All Fields] OR ("myositides"[All Fields] AND "infectious"[All Fields])) OR ("myositis"[MeSH Terms] OR "myositis"[All Fields] OR ("infectious"[All Fields] AND "myositis"[All Fields]) OR "infectious myositis"[All Fields]) OR ("myositis"[MeSH Terms] OR "myositis"[All Fields] OR ("idiopathic"[All Fields] AND "inflammatory"[All Fields] AND "myopathies"[All Fields]) OR "idiopathic inflammatory myopathies"[All Fields]) OR ("myositis"[MeSH Terms] OR "myositis"[All Fields] OR ("myopathy"[All Fields] AND "idiopathic"[All Fields] AND "inflammatory"[All Fields]) OR "myopathy idiopathic inflammatory"[All Fields]) OR ("myositis"[MeSH Terms] OR "myositis"[All Fields] OR ("inflammatory"[All Fields] AND "myopathies"[All Fields] AND "idiopathic"[All Fields])) OR ("myositis"[MeSH Terms] OR "myositis"[All Fields] OR ("myopathies"[All Fields] AND "idiopathic"[All Fields] AND "inflammatory"[All Fields]) OR "myopathies idiopathic inflammatory"[All Fields]) OR ("myositis"[MeSH Terms] OR "myositis"[All Fields] OR ("idiopathic"[All Fields] AND "inflammatory"[All Fields] AND "myopathy"[All Fields]) OR "idiopathic inflammatory myopathy"[All Fields]) OR ("myositis"[MeSH Terms] OR "myositis"[All Fields] OR ("idiopathic"[All Fields] AND "inflammatory"[All Fields] AND "myositis"[All Fields]) OR "idiopathic inflammatory myositis"[All Fields]) OR ("myositis"[MeSH Terms] OR "myositis"[All Fields] OR ("inflammatory"[All Fields] AND "myopathy"[All Fields] AND "idiopathic"[All Fields])) | 51,988 |
| 5 | "Myositis"[Mesh] | "Myositis"[MeSH Terms] | 24,084 |
| 4 | ((((scleroderma) OR (Sclerosis, Systemic)) OR (Systemic Scleroderma)) OR (Systemic Sclerosis)) OR (Scleroderma, Systemic) | "scleroderma, systemic"[MeSH Terms] OR ("scleroderma"[All Fields] AND "systemic"[All Fields]) OR "systemic scleroderma"[All Fields] OR "scleroderma"[All Fields] OR "scleroderma, localized"[MeSH Terms] OR ("scleroderma"[All Fields] AND "localized"[All Fields]) OR "localized scleroderma"[All Fields] OR "sclerodermas"[All Fields] OR ("scleroderma, systemic"[MeSH Terms] OR ("scleroderma"[All Fields] AND "systemic"[All Fields]) OR "systemic scleroderma"[All Fields] OR ("sclerosis"[All Fields] AND "systemic"[All Fields]) OR "sclerosis systemic"[All Fields]) OR ("scleroderma, systemic"[MeSH Terms] OR ("scleroderma"[All Fields] AND "systemic"[All Fields]) OR "systemic scleroderma"[All Fields] OR ("systemic"[All Fields] AND "scleroderma"[All Fields])) OR ("scleroderma, systemic"[MeSH Terms] OR ("scleroderma"[All Fields] AND "systemic"[All Fields]) OR "systemic scleroderma"[All Fields] OR ("systemic"[All Fields] AND "sclerosis"[All Fields]) OR "systemic sclerosis"[All Fields]) OR ("scleroderma, systemic"[MeSH Terms] OR ("scleroderma"[All Fields] AND "systemic"[All Fields]) OR "systemic scleroderma"[All Fields] OR "scleroderma systemic"[All Fields]) | 43,275 |
| 3 | "Scleroderma, Systemic"[Mesh] | "scleroderma, systemic"[MeSH Terms] | 24,510 |
| 2 | ((rheumatoid arthritis) OR (Rheumatoid Arthritis)) OR (Arthritis, Rheumatoid) | "arthritis, rheumatoid"[MeSH Terms] OR ("arthritis"[All Fields] AND "rheumatoid"[All Fields]) OR "rheumatoid arthritis"[All Fields] OR ("rheumatoid"[All Fields] AND "arthritis"[All Fields]) OR ("arthritis, rheumatoid"[MeSH Terms] OR ("arthritis"[All Fields] AND "rheumatoid"[All Fields]) OR "rheumatoid arthritis"[All Fields] OR ("rheumatoid"[All Fields] AND "arthritis"[All Fields])) OR ("arthritis, rheumatoid"[MeSH Terms] OR ("arthritis"[All Fields] AND "rheumatoid"[All Fields]) OR "rheumatoid arthritis"[All Fields] OR "arthritis rheumatoid"[All Fields]) | 181,699 |
| 1 | "Arthritis, Rheumatoid"[Mesh] | "arthritis, rheumatoid"[MeSH Terms] | 133,201 |

# Supplementary Table 2: Basic Characteristics and Quality Assessment Results of Included Studies

| **Included studies** | **Study design** | **Study**  **Country** | **Duration of the Study** | **Mean age (years)** | **Total sample size (male/female)** | **Number of PPF cases** | **Incidence rate (%)** | **Disease type** | **CTD type** | **Influencing factors** | **NOS score** |
| --- | --- | --- | --- | --- | --- | --- | --- | --- | --- | --- | --- |
| Xueyan Shan, 2025 | Single-center retrospective cohort study | China | 2018.1-2022.12 | 54.5±10.9 | 69（18/51） | 27 | 39.13 | IIM-ILD | anti-PL7-positive ASS | 1,2 | 8 |
| Sung Hae Chang, 2025 | Multicenter prospective cohort study | South Korea | 2015.1-2020.10 | 66.4±8.2 | 138（42/96） | 48 | 34.78 | RA-ILD | RA | 5,6,7,8,9,10,11,12,13,14,15 | 8 |
| Anna-Maria Hoffmann-Vold, 2025 | Multicenter prospective cohort study | Norway/Switzerland | 2001.1-2019.12 | 48.0±14.6 | 231（55/176） | 39 | 16.88 | SSc-ILD | SSc | - | 8 |
| Sung Hae Chang, 2025 | Multicenter prospective cohort study | South Korea | 2015.1-2021.9 | 66.5±8.3 | 136（41/95） | 47 | 34.56 | RA-ILD | RA | 14,16 | 8 |
| Noboro Sato, 2025 | Single-center retrospective cohort study | Japan | 2015.4-2022.3 | - | 79（22/57） | 14 | 17.72 | IIM-ILD | PM, DM, CADM | - | 6 |
| Kathleen Morrisroe, 2024 | Multicenter prospective cohort study | Australia | 2008.1-2022.11 | - | 464（85/379） | 180 | 38.79 | SSc-ILD | SSc | 17,18,19,20 | 8 |
| Yura Ahn, 2024 | Single-center retrospective cohort study | South Korea | 2007.4-2022.10 | 62.8±11.7 | 97（37/60） | 14 | 14.43 | CTD-ILD | RA, IIM, SSc, pSS, SLE, UCTD, MCTD | - | 8 |
| Yuanying Wang, 2024 | Single-center prospective cohort study | China | 2018.1-2023.1 | 60.1±11.6 | 224（80/144） | 63 | 28.13 | CTD-ILD | IIM, pSS, UCTD, OCTD, RA, SSc, SLE | 2,3 | 8 |
| Kinan El Husseini, 2025 | Multicenter prospective cohort study | France | - | 66.1±10.2 | 101（46/55） | 33 | 32.67 | RA-ILD | RA | 21,22 | 9 |
| Andreina Manfredi, 2024 | Multicenter prospective cohort study | Italy | 2022.7-2023.1 | - | 72（14/58） | 26 | 36.11 | pSS-ILD | pSS | - | 7 |
| Jongmin Lee, 2023 | Single-center retrospective cohort study | South Korea | 2019.1-2022.5 | - | 107（18/89） | 41 | 38.32 | CTD-ILD | SSc, RA, pSS, IIM, SLE, MCTD, OCTD | - | 7 |
| Ju Kwang Lee, 2023 | Single-center retrospective cohort study | South Korea | 2007.4-2022.10 | - | 197（77/120） | 37 | 18.78 | CTD-ILD | RA, IIM, SSc, SLE, pSS, UCTD, MCTD | 14 | 8 |
| Huijuan Wang, 2023 | Single-center retrospective cohort study | China | 2017.1-2022.6 | 58.6±11.3 | 307（109/198） | 94 | 30.62 | IIM-ILD | anti-MSA positive IIM | 12,23,24,25 | 8 |
| Kathleen Morrisroe, 2025 | Multicenter prospective cohort study | Australia | 2008.1-2024.11 | 58.3±13.1 | 356（71/285） | 52 | 14.61 | SSc-ILD | SSc | - | 8 |
| Yu-Hsiang Chiu, 2023 | Single-center retrospective cohort study | Netherlands | 2005-2021 | - | 230（108/122） | 53 | 23.04% | CTD-ILD | RA, IIM, pSS, UCTD, SSc, MCTD, SLE, OCTD, SpA, ANCA | - | 9 |
| Jia-Jia Fan, 2024 | Single-center retrospective cohort study | China | 2015.1-2022.12 | - | 120 | 52 | 44.07% | CTD-ILD | SSc, RA, IIM, ASS, MCTD, ANCA, MPA, AOSD, AS | - | 8 |
| Ji Hoon Jang, 2024 | Single-center retrospective cohort study | South Korea | 2010.1-2023.6 | - | 95（36/59） | 45 | 47.37% | CTD-ILD | CTD | - | 8 |
| Jakob Höppner, 2024 | Single-center retrospective cohort study | Germany | 2018.1-2022.5 | 60.1±12.9 | 50（17/33） | 23 | 46% | CTD-ILD | RA, SSc, IIM, pSS, SpA, SLE, MCTD, UCTD | - | 6 |
| Anaïs Roeser, 2025 | Single-center retrospective cohort study | France | 2006.1-2022.12 | - | 79（18/61） | 18 | 22.78% | SSc-ILD | SSc | 26,27 | 9 |
| Hongyan Fu, 2023 | Single-center retrospective cohort study | China | 2017.10-2020.6 | 56.6±12.6 | 72（21/51） | 18 | 25.00% | IIM-ILD | PM, DM | 14,30,31 | 8 |
| Jianping Diao, 2025 | Multicenter retrospective case-control study | China | 2010.6-2023.10 | 54.1±11.3 | 66（3/63） | 29 | - | pSS-ILD | pSS | 12,28,29 | 8 |
| Jiamin Song, 2025 | Single-center retrospective case-control study | China | 2015.1-2023.9 | 63.2±10.7 | 58（12/46） | 11 | - | pSS-ILD | pSS | 3,4 | 8 |

Note: ①Case-control studies were excluded from the incidence rate meta-analysis and only included in the analysis of influencing factors. ②Influencing factors: 1=Lactate dehydrogenase (LDH), 2=Carbohydrate antigen 125 (CA-125), 3=Carcinoembryonic antigen (CEA), 4=Carbohydrate antigen 50 (CA-50), 5=Low disease activity (DAS28-ESR), 6=Definite/probable UIP pattern on imaging, 7=ILD involvement extent >10%, 8=Total fibrosis score, 9=Reticular opacity score, 10=Traction bronchiectasis/bronchiolectasis score, 11=Honeycombing score, 12=Diffusing capacity for carbon monoxide (DLCO), 13=Anti-CCP antibodies, 14=KL-6, 15=hSP-D, 16=Matrix Metalloproteinase-7 (MMP-7), 17=Advanced age, 18=Diffuse cutaneous systemic sclerosis (dcSSc), 19=Anti-centromere antibody, 20=Anti-Scl-70 antibody, 21=forced vital capacity percentage predicted (FVC% predicted), 22=Short telomere length, 23=Acute/subacute ILD onset, 24=Diffuse alveolar damage (DAD) on HRCT, 25=Triple therapy with glucocorticoids, immunosuppressants and antifibrotic drugs, 26=Pulmonary hypertension, 27=Short-term progression, 28=Parotid gland enlargement, 29=Arthritis, 30=Neutrophil-to-lymphocyte ratio (NLR), 31=Positive non-Jo-1 antibodies. ③Among CTD subtypes including UCTD, OCTD, SpA, ANCA, MPA, AOSD, and AS, the incidence of PPF remains unclear due to insufficient data on PPF occurrence cases and total sample sizes in included literature, preventing Meta-analysis or descriptive analysis.

# Supplementary Table 3: Assessment of consistency with the PPF definition

| **Included studies** | **Observation window** | **Symptoms assessed**  **(Yes or No)** | **Physiologic criteria**  **(Yes or No)** | **HRCT criteria**  **(Yes or No)** | **Consistency with 2022 guideline*（Full/Partial/Deviated）** |
| --- | --- | --- | --- | --- | --- |
| Xueyan Shan, 2025 | 12 months | Yes (worsening respiratory symptoms) | Yes (an absolute decline in FVC >5% predicted and/or DLCO corrected for Hb >10% predicted) | Yes (radiologically demonstrated expansion of fibrosis extent) | Full |
| Jiamin Song, 2025 | 12 months | Yes (worsening respiratory symptoms) | Yes (an absolute decline in FVC >5% predicted and/or DLCO corrected for Hb >10% predicted) | Yes (increased extent/severity of fibrotic features, e.g., aggravated traction bronchiectasis, new ground-glass opacities with bronchiectasis/fine reticulation, expanded and coarsened reticulation, increased honeycombing, reduced lung volume) | Full |
| Sung Hae Chang, 2025 | 12 months | No (Relevant data not collected) | Yes (an absolute decline in FVC >5% predicted and/or DLCO corrected for Hb >10% predicted) | Yes (increased lesion extent/severity, or new reticulation/honeycombing/ground-glass opacities with bronchiectasis) | Partial |
| Hoffmann-Vold AM, 2025 | 12 months | Yes (worsening respiratory symptoms) | Yes (an absolute decline in FVC >5% predicted and/or DLCO corrected for Hb >10% predicted) | Yes (radiologists confirmed ILD progression according to guidelines) | Full |
| Sung Hae Chang, 2025 | 12 months | No (Relevant data not collected) | Yes (an absolute decline in FVC >5% predicted and/or DLCO corrected for Hb >10% predicted) | Yes (increased lesion extent/severity, or new ground-glass opacities with bronchiectasis/reticulation/honeycombing) | Partial |
| Noboro Sato, 2025 | 12 months | Yes (worsening respiratory symptoms) | Yes (an absolute decline in FVC >5% predicted and/or DLCO corrected for Hb >10% predicted) | Yes (increased extent/severity of traction bronchiectasis, new ground-glass opacities with bronchiectasis/fine reticulation, expanded and coarsened reticulation, increased honeycombing, aggravated lobar volume loss) | Full |
| Kathleen Morrisroe, 2024 | 12 months | Yes (worsening respiratory symptoms) | Yes (an absolute decline in FVC >5% predicted and/or DLCO corrected for Hb >10% predicted) | Yes (repeat HRCT within 12 months of ILD diagnosis showed expanded fibrosis extent) | Full |
| Yura Ahn, 2024 | 12 months | Yes (worsening respiratory symptoms) | Yes (an absolute decline in FVC >5% predicted and/or DLCO corrected for Hb >10% predicted) | Yes (Visual assessment: aggravated bronchiectasis, new ground-glass opacities with bronchiectasis/fine reticulation, expanded and coarsened reticulation, increased honeycombing; or quantitative assessment: ΔQILD＞=4%) | Full |
| Yuanying Wang, 2024 | 12 months | Yes (worsening respiratory symptoms) | Yes (an absolute decline in FVC >5% predicted and/or DLCO corrected for Hb >10% predicted) | Yes (radiological progression determined by 2 ILD experts via blinded assessment, discrepancies resolved by consensus) | Full |
| Kinan El Husseini, 2025 | 12 months | No (Relevant data not collected) | Yes (an absolute decline in FVC >5% predicted and/or DLCO corrected for Hb >10% predicted) | No (longitudinal imaging data not systematically collected, not included in progression definition) | Partial |
| Andreina Manfredi, 2024 | 24 months | No (Relevant data not collected) | Yes (an absolute decline in FVC >5% predicted and/or DLCO corrected for Hb >10% predicted) | Yes (increased extent/severity of traction bronchiectasis, new ground-glass opacities with bronchiectasis/fine reticulation, expanded and coarsened reticulation, increased honeycombing, aggravated lobar volume loss) | Partial |
| Jongmin Lee, 2023 | 12 months | Yes (worsening respiratory symptoms) | Yes (an absolute decline in FVC >5% predicted and/or DLCO corrected for Hb >10% predicted) | Yes (increased extent/severity of traction bronchiectasis, new ground-glass opacities with bronchiectasis/fine reticulation, expanded and coarsened reticulation, increased honeycombing, aggravated lobar volume loss) | Full |
| Ju Kwang Lee, 2023 | 12 months | Yes (worsening respiratory symptoms) | Yes (an absolute decline in FVC >5% predicted and/or DLCO corrected for Hb >10% predicted) | Yes (blinded assessment by 2 radiologists, discrepancies resolved by consensus, showing increased fibrosis extent) | Full |
| Huijuan Wang, 2023 | 12 months | Yes (worsening respiratory symptoms) | Yes (an absolute decline in FVC >5% predicted and/or DLCO corrected for Hb >10% predicted) | Yes (increased extent/severity of traction bronchiectasis, new ground-glass opacities with bronchiectasis/fine reticulation, expanded and coarsened reticulation, increased honeycombing, aggravated lobar volume loss) | Full |
| Kathleen Morrisroe, 2025 | 12 months | Yes (worsening respiratory symptoms) | Yes (an absolute decline in FVC >5% predicted and/or DLCO corrected for Hb >10% predicted) | Yes (radiologically demonstrated fibrotic progression, in accordance with guideline definitions) | Full |
| Yu-Hsiang Chiu, 2023 | 12 months | Yes (worsening respiratory symptoms) | Yes (an absolute decline in FVC >5% predicted and/or DLCO corrected for Hb >10% predicted) | Yes (blinded assessment by 2 thoracic radiologists, showing fibrotic progression) | Full |
| Jia-Jia Fan, 2024 | 12 months | Yes (worsening respiratory symptoms) | Yes (an absolute decline in FVC >5% predicted and/or DLCO corrected for Hb >10% predicted) | Yes (increased extent/severity of traction bronchiectasis, new ground-glass opacities with bronchiectasis/fine reticulation, expanded and coarsened reticulation, increased honeycombing, aggravated lobar volume loss) | Full |
| Ji Hoon Jang, 2024 | 12 months | Yes (worsening respiratory symptoms) | Yes (an absolute decline in FVC >5% predicted and/or DLCO corrected for Hb >10% predicted) | Yes (determined by radiology experts: increased extent/severity of bronchiectasis, new ground-glass opacities with bronchiectasis/fine reticulation, expanded and coarsened reticulation, increased honeycombing, aggravated lobar volume loss) | Full |
| Jakob Höppner, 2024 | 12 months | No (Relevant data not collected) | Yes (an absolute decline in FVC >5% predicted and/or DLCO corrected for Hb >10% predicted) | Yes (determined by radiologists during multidisciplinary discussion: increased extent/severity of bronchiectasis, new ground-glass opacities with bronchiectasis/fine reticulation, expanded and coarsened reticulation, increased honeycombing, aggravated lobar volume loss) | Partial |
| Anaïs Roeser, 2025 | 12 months | Yes (worsening respiratory symptoms) | Yes (an absolute decline in FVC >5% predicted and/or DLCO corrected for Hb >10% predicted) | Yes (central review by radiologists with 20 years of experience: increased extent/severity of bronchiectasis, new ground-glass opacities with bronchiectasis/fine reticulation, expanded and coarsened reticulation, increased honeycombing, aggravated lobar volume loss) | Full |
| Jianping Diao, 2025 | 12 months | Yes (worsening respiratory symptoms) | Yes (an absolute decline in FVC >5% predicted and/or DLCO corrected for Hb >10% predicted) | Yes (increased extent of reticulation/bronchiectasis, new ground-glass opacities with bronchiectasis/reticulation, coarsened reticulation, aggravated honeycombing, reduced lung volume) | Full |
| Hongyan Fu, 2023 | 12 months | Yes (worsening respiratory symptoms) | Yes (an absolute decline in FVC >5% predicted and/or DLCO corrected for Hb >10% predicted) | Yes (blinded assessment by 2 thoracic radiologists, discrepancies resolved by consensus, showing extension of fibrotic manifestations such as reticulation, bronchiectasis, and honeycombing) | Full |

# Meta-Analysis Results of PPF Incidence in CTD-ILD

## Subgroup Analysis

**4.1.1 Specific Types of CTD**


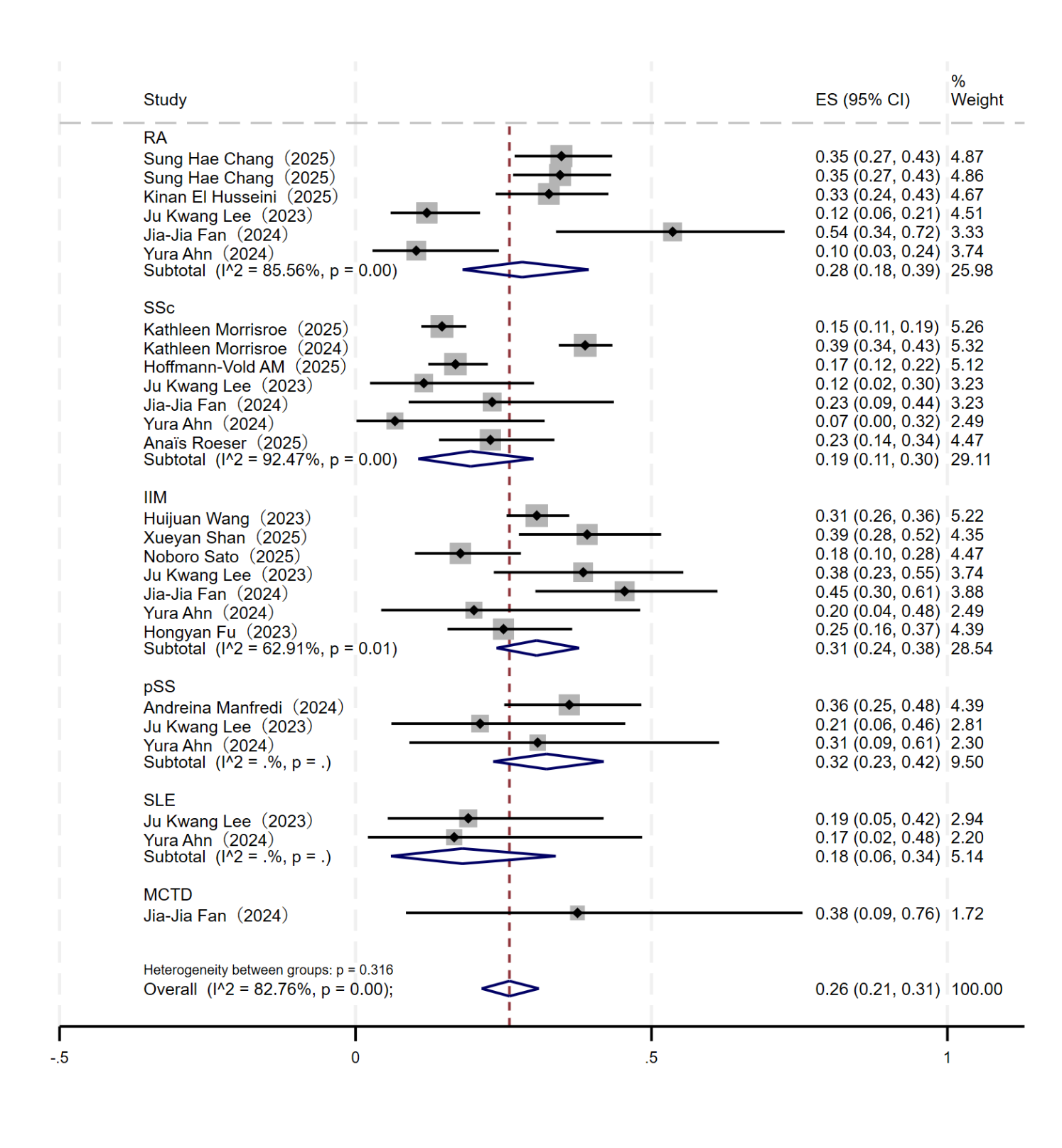


**Figure 1** Forest plot of subgroup analysis of PPF incidence in patients with CTD-ILD by specific types of CTD

**4.1.2 Mean age**


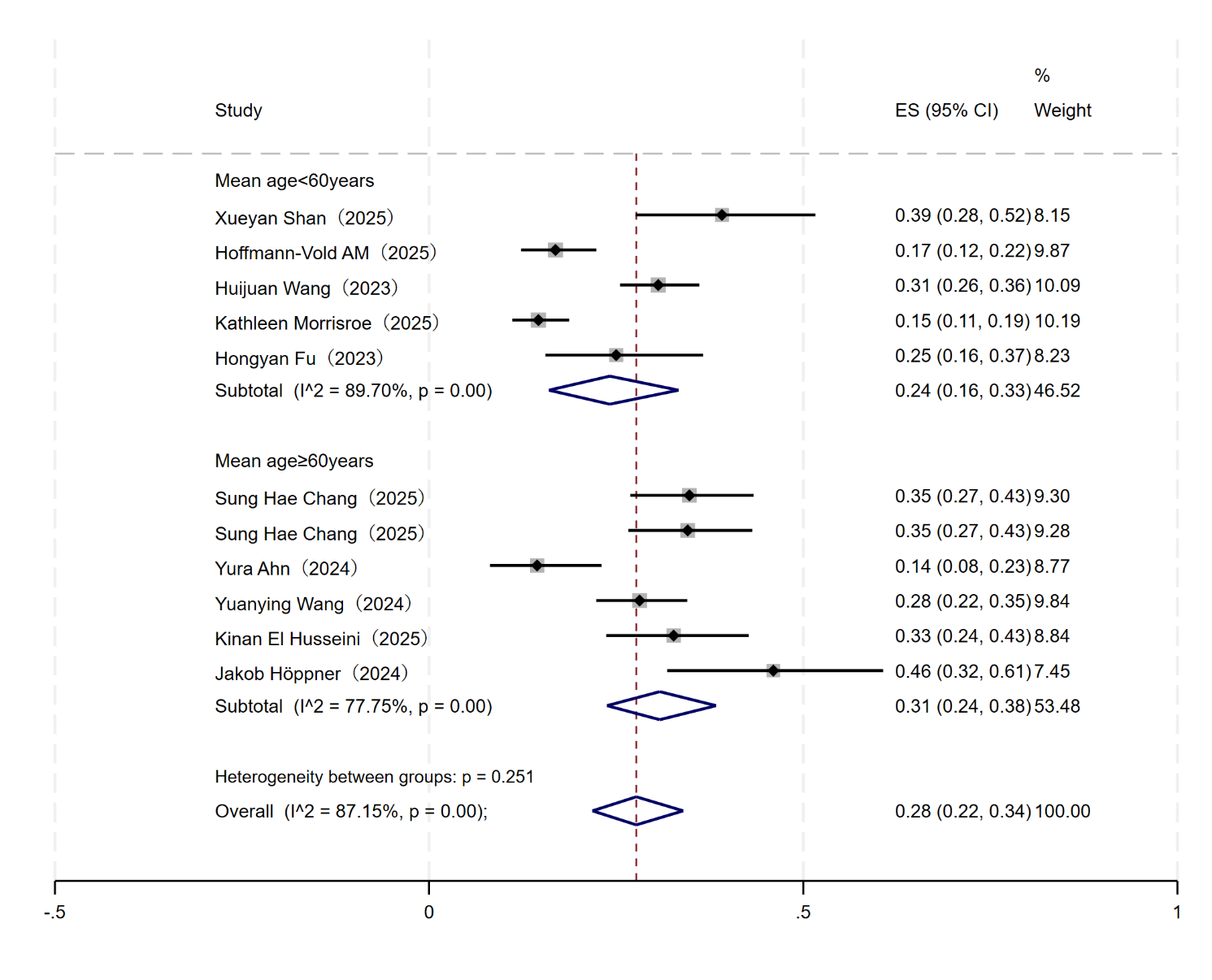


**Figure 2** Forest plot of subgroup analysis of PPF incidence in patients with CTD-ILD by age

**4.1.3 Region**


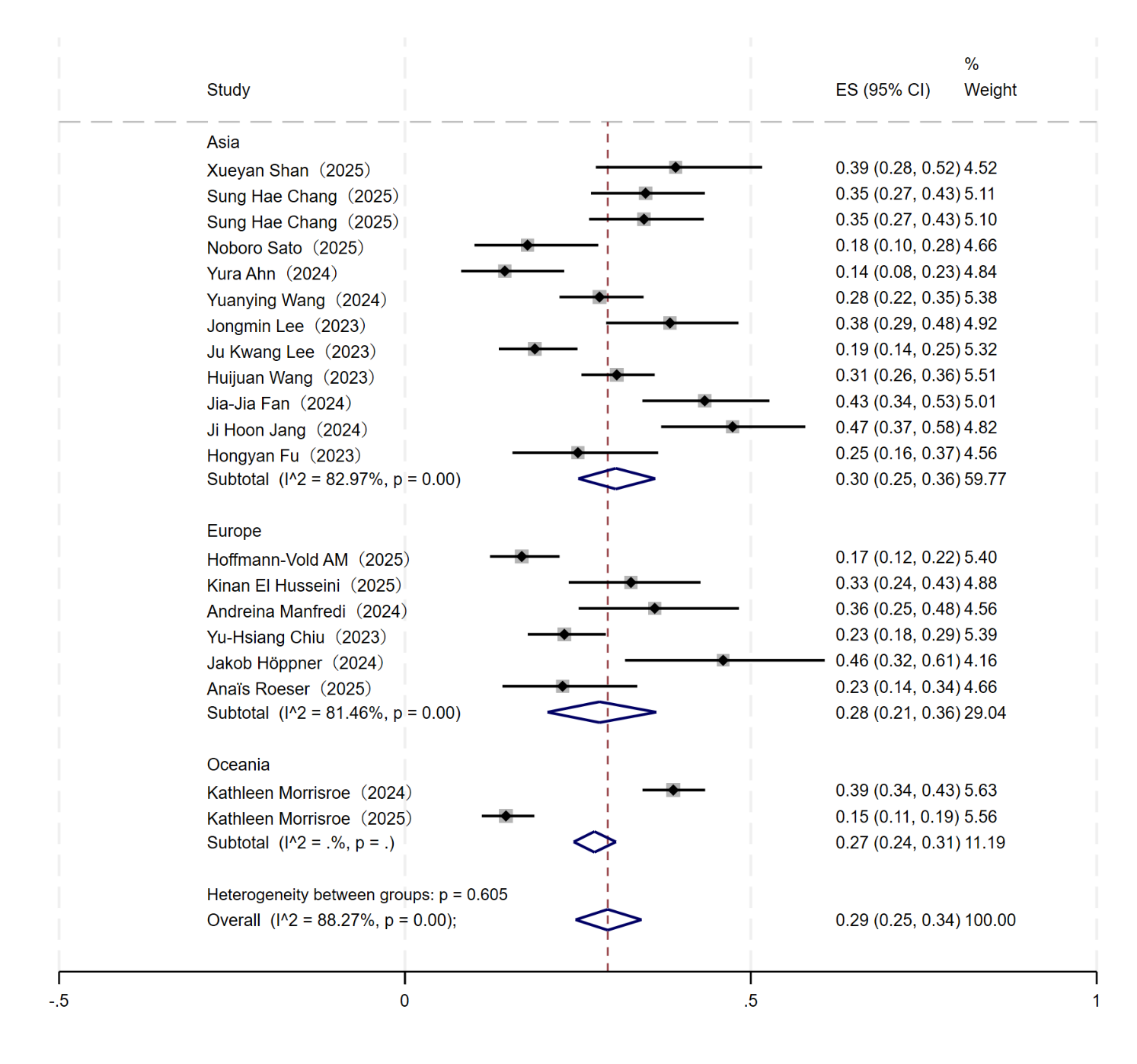


**Figure 3** Forest plot of subgroup analysis of PPF incidence in patients with CTD-ILD by region

**4.1.4 Gender**


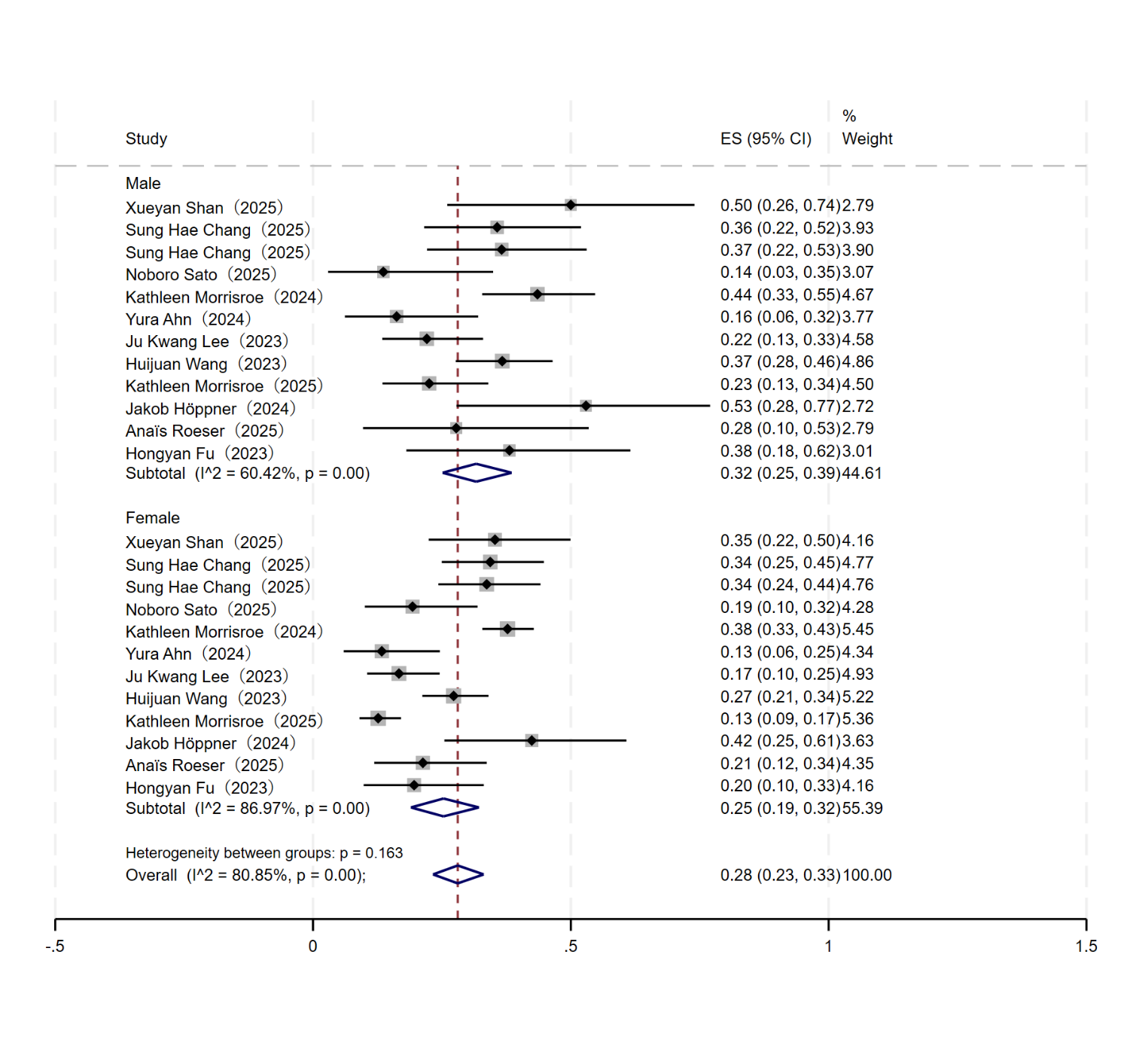


**Figure 4** Forest plot of subgroup analysis of PPF incidence in patients with CTD-ILD by gender

## Meta-regression analysis

**4.2.1 Specific Types of CTD**


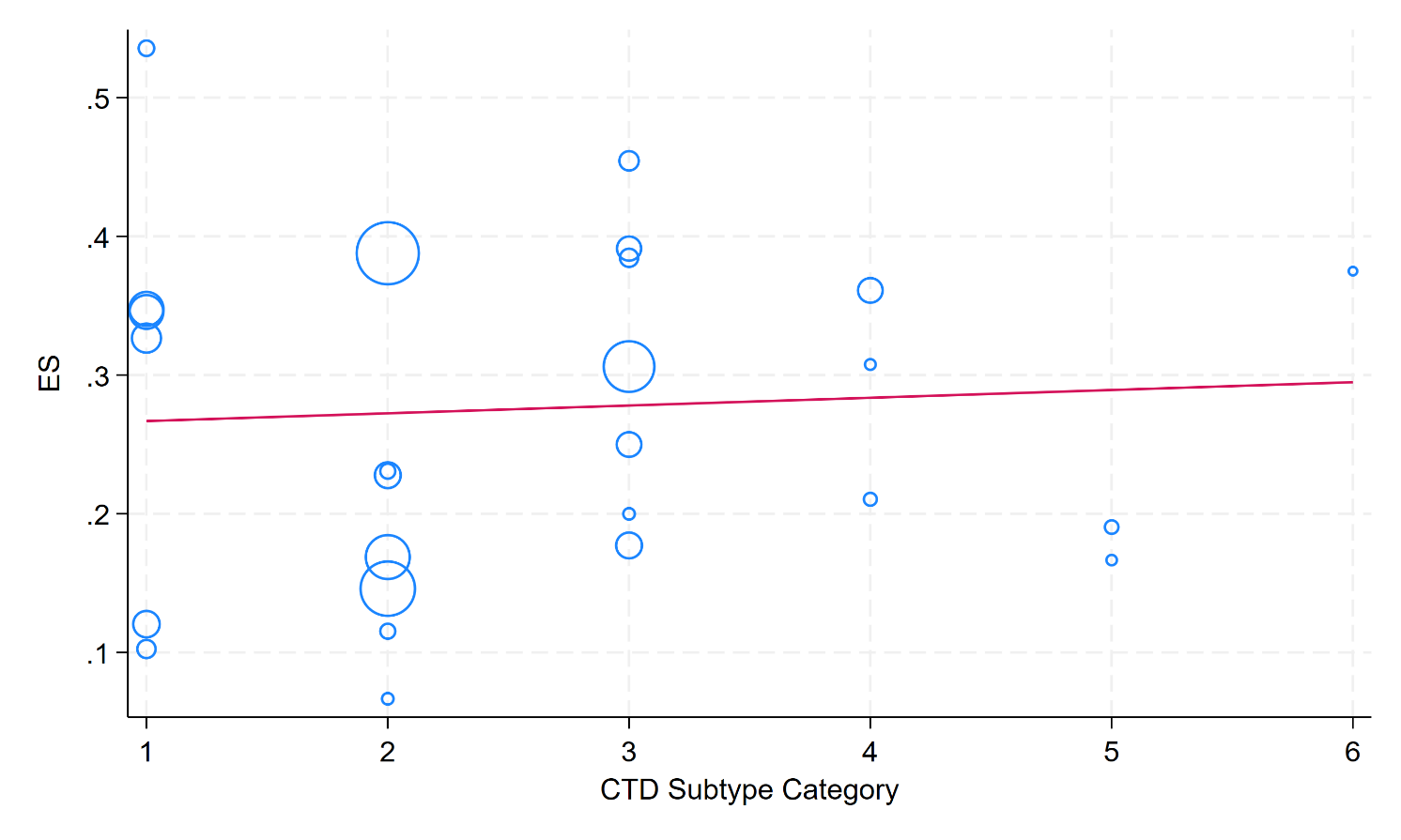
 **Figure 5** Meta-regression bubble plot of the incidence of PPF in patients with CTD-ILD using CTD subtypes as a covariate

**4.2.2 Mean age**


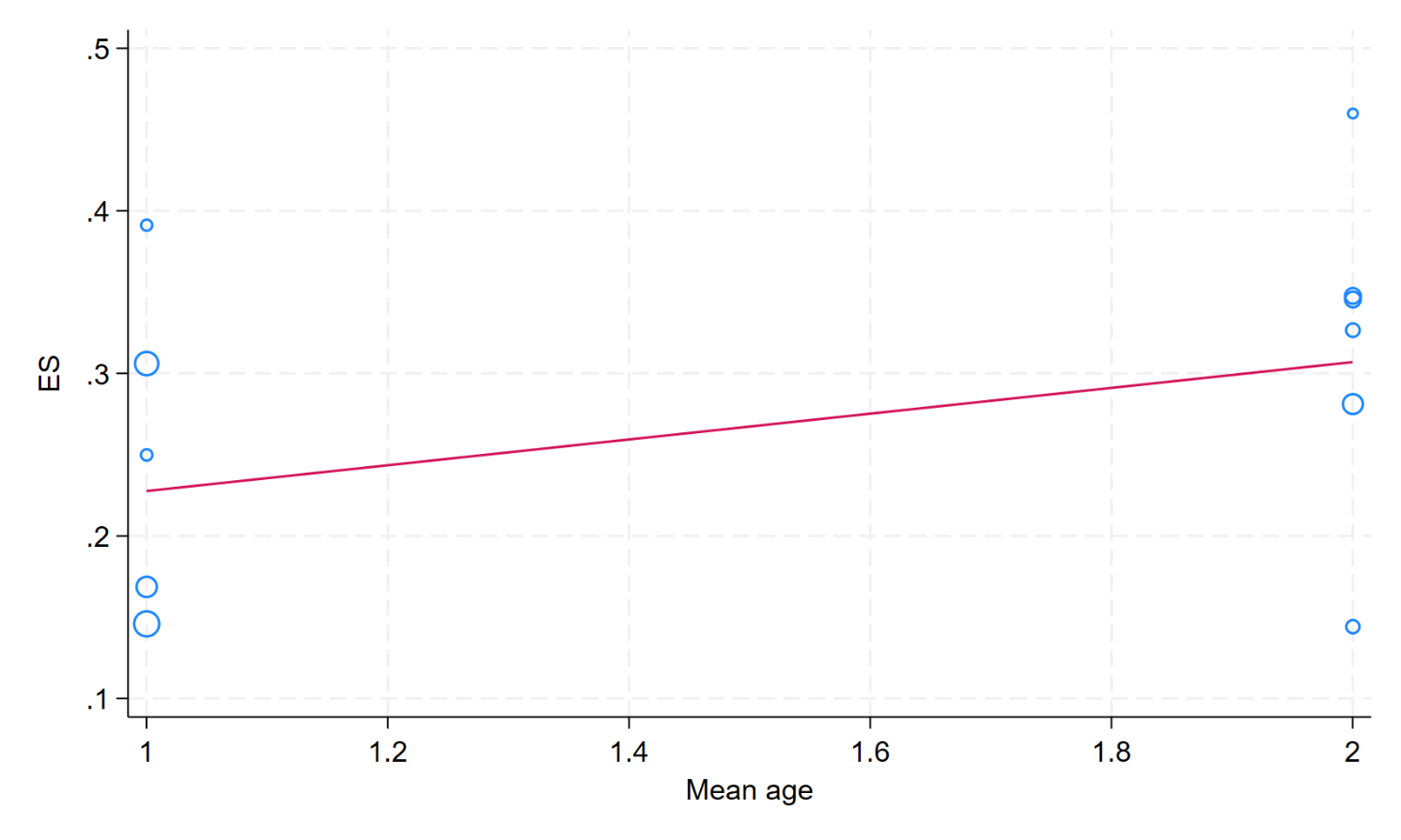


**Figure 6** Meta-regression bubble plot of the incidence of PPF in patients with CTD-ILD using Mean age as a covariate

**4.2.3 Region**


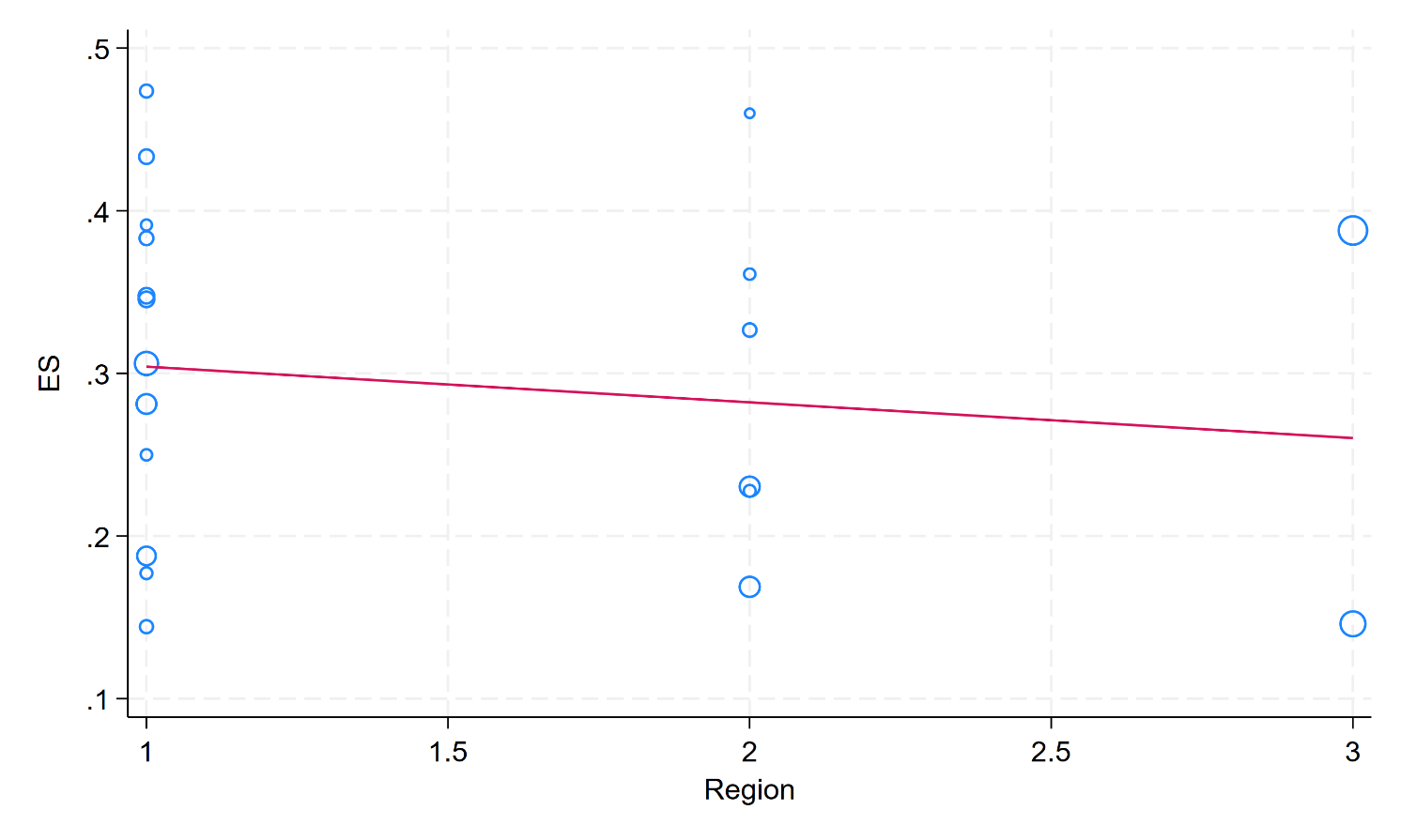


**Figure 7** Meta-regression bubble plot of the incidence of PPF in patients with CTD-ILD using geographic region as a covariate

**4.2.4 Gender**


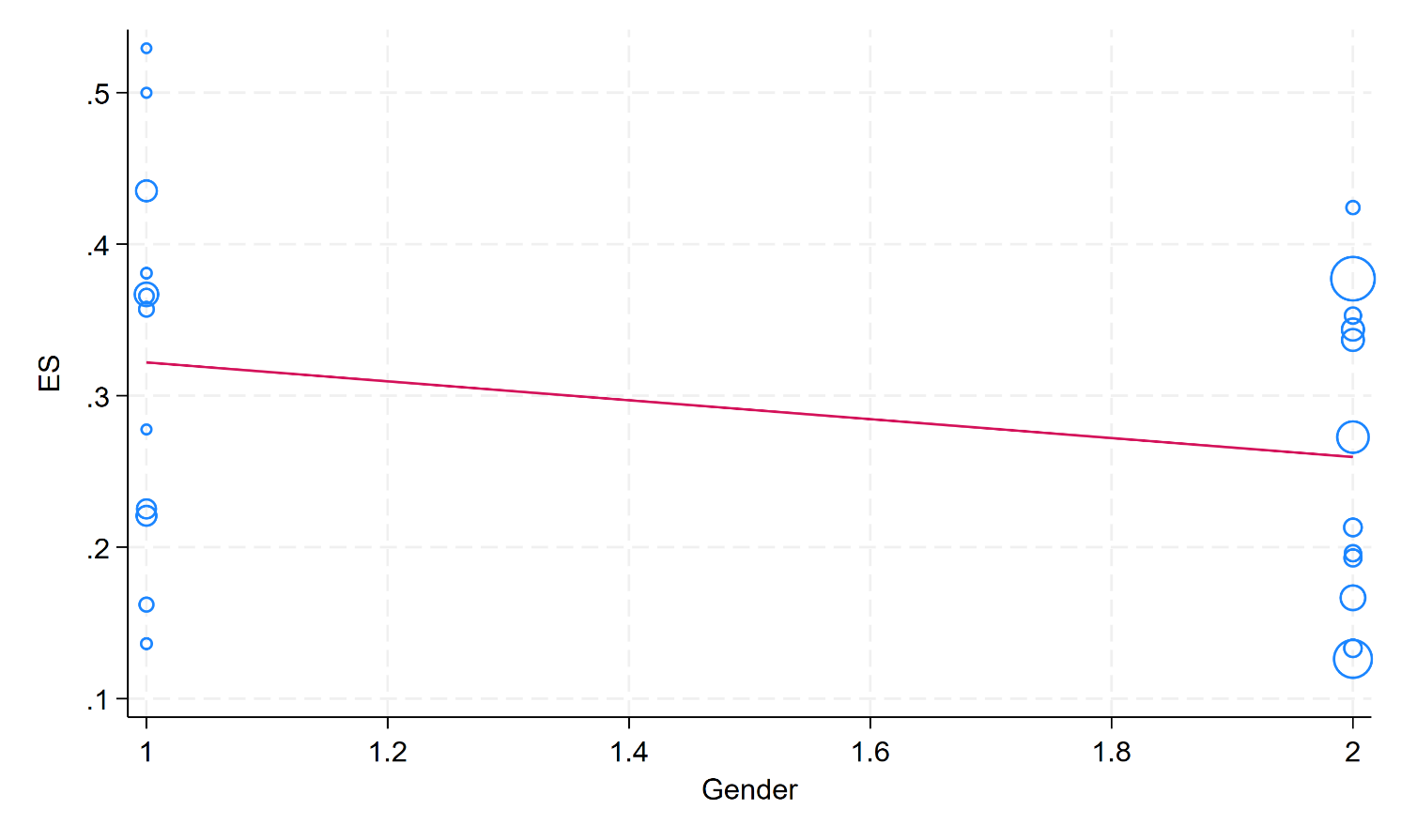


**Figure 8** Meta-regression bubble plot of the incidence of PPF in patients with CTD-ILD using gender as a covariate

## Sensitivity analysis

**
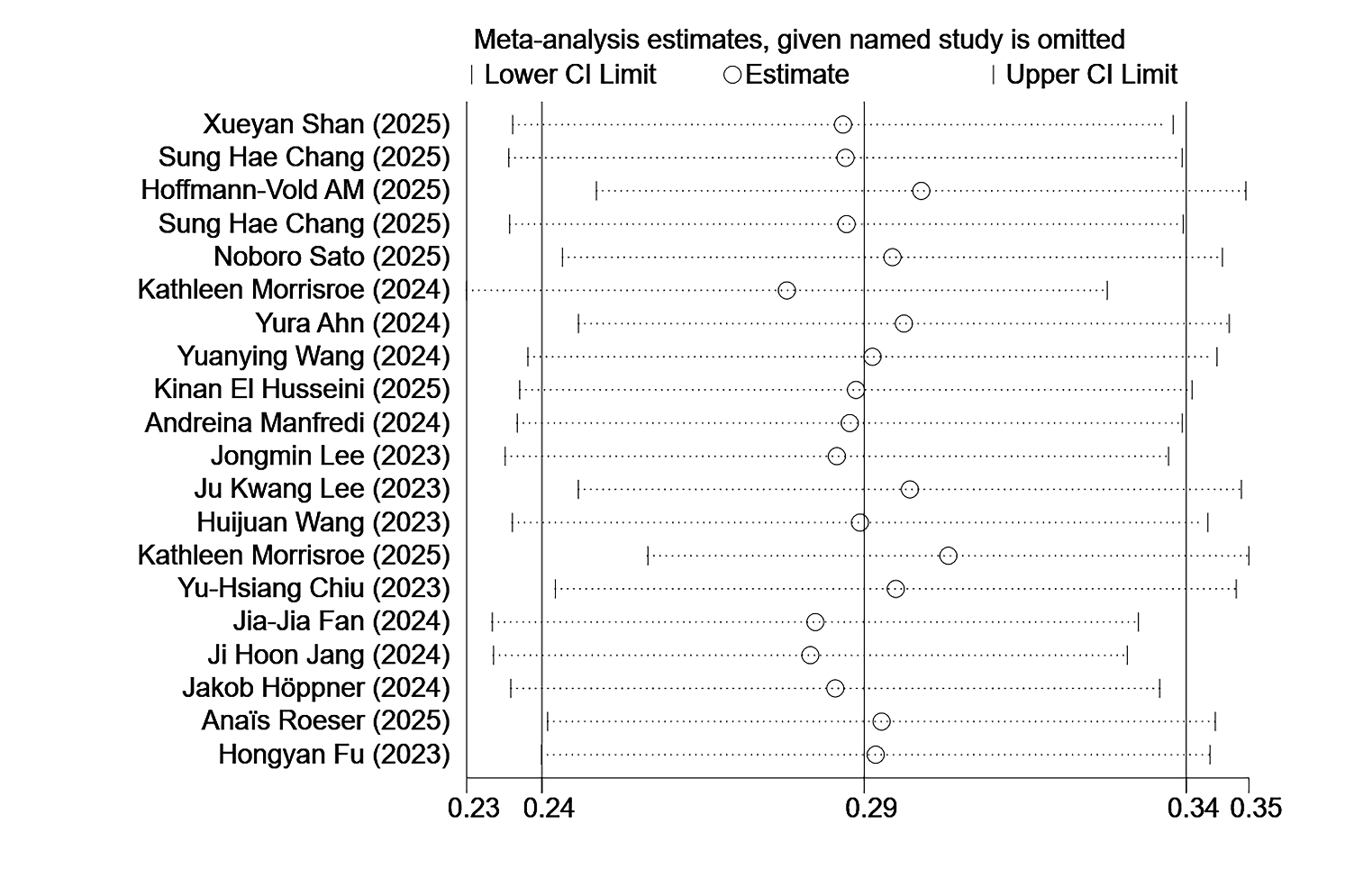
**

**Figure 9** Sensitivity analysis of the overall incidence of PPF in patients with CTD-ILD

## Bias Assessment


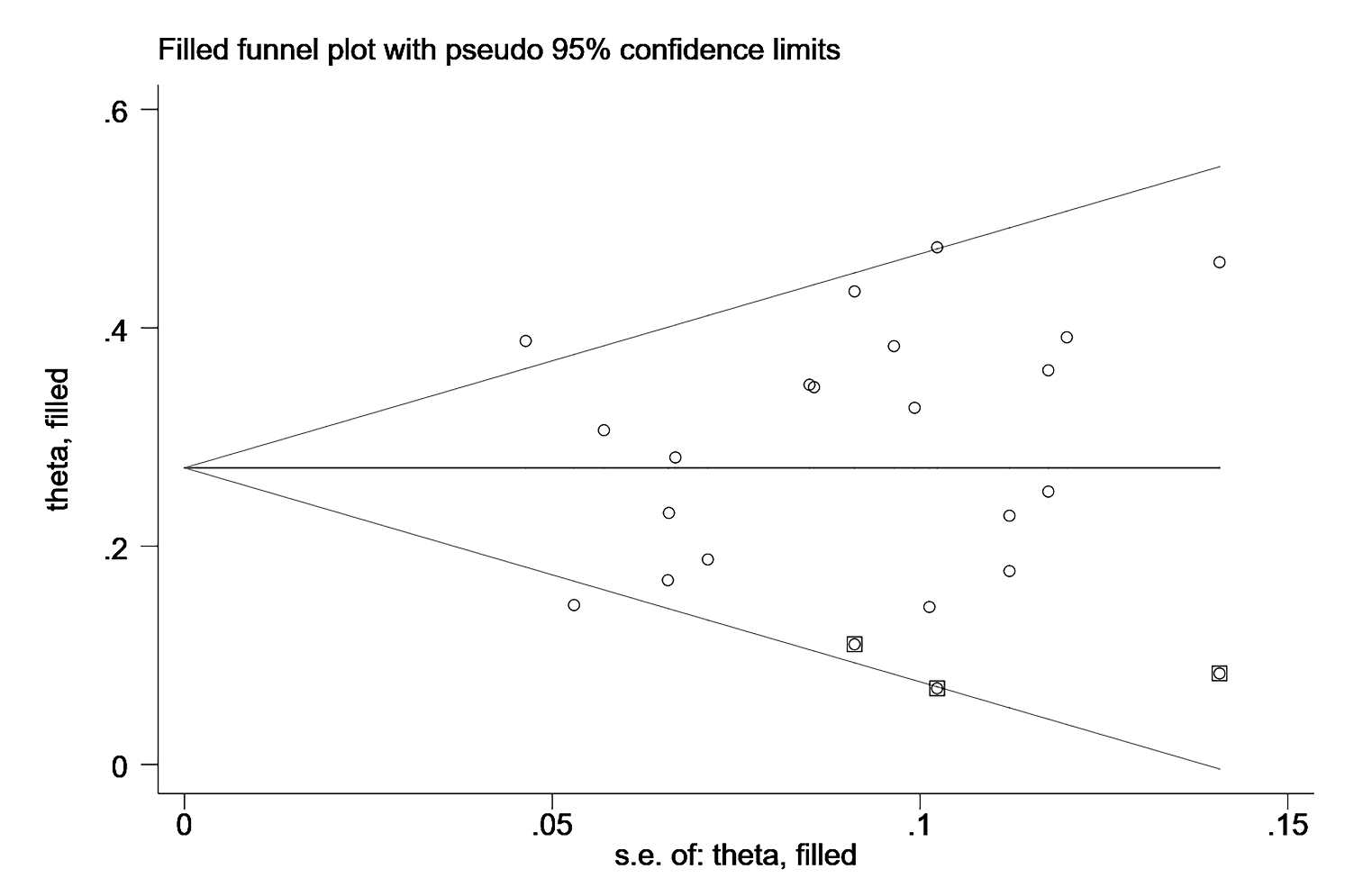


**Figure 10** Filled funnel plot of the overall incidence of PPF in patients with CTD-ILD

## Localization of Heterogeneity Sources and Extreme Value Verification


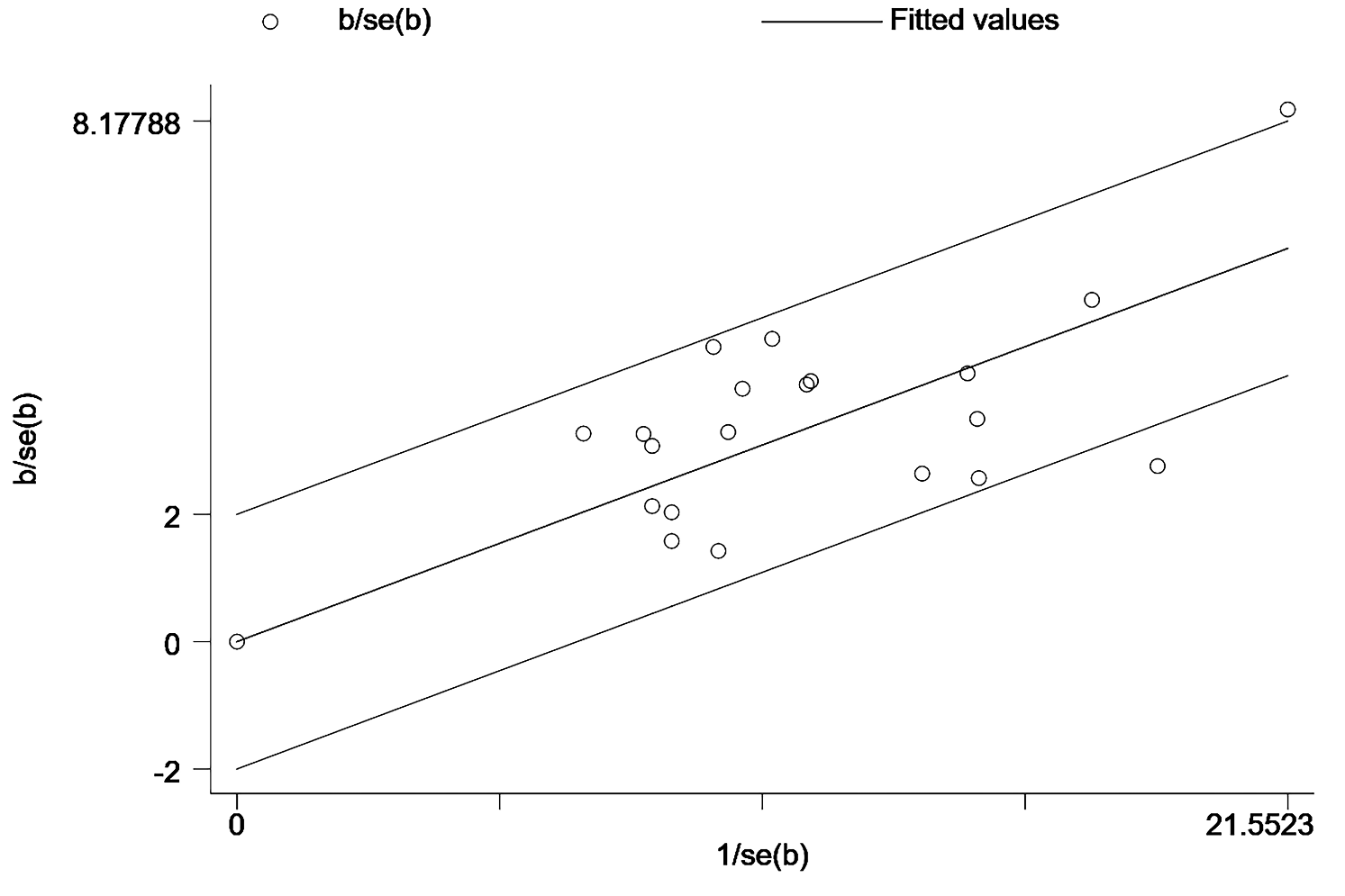


**Figure 11** Galbraith plot of the overall incidence of PPF in patients with CTD-ILD

# Meta-Analysis Results of Factors Influencing PPF Occurrence in Patients with CTD-ILD

## Forest Plot of CA-125


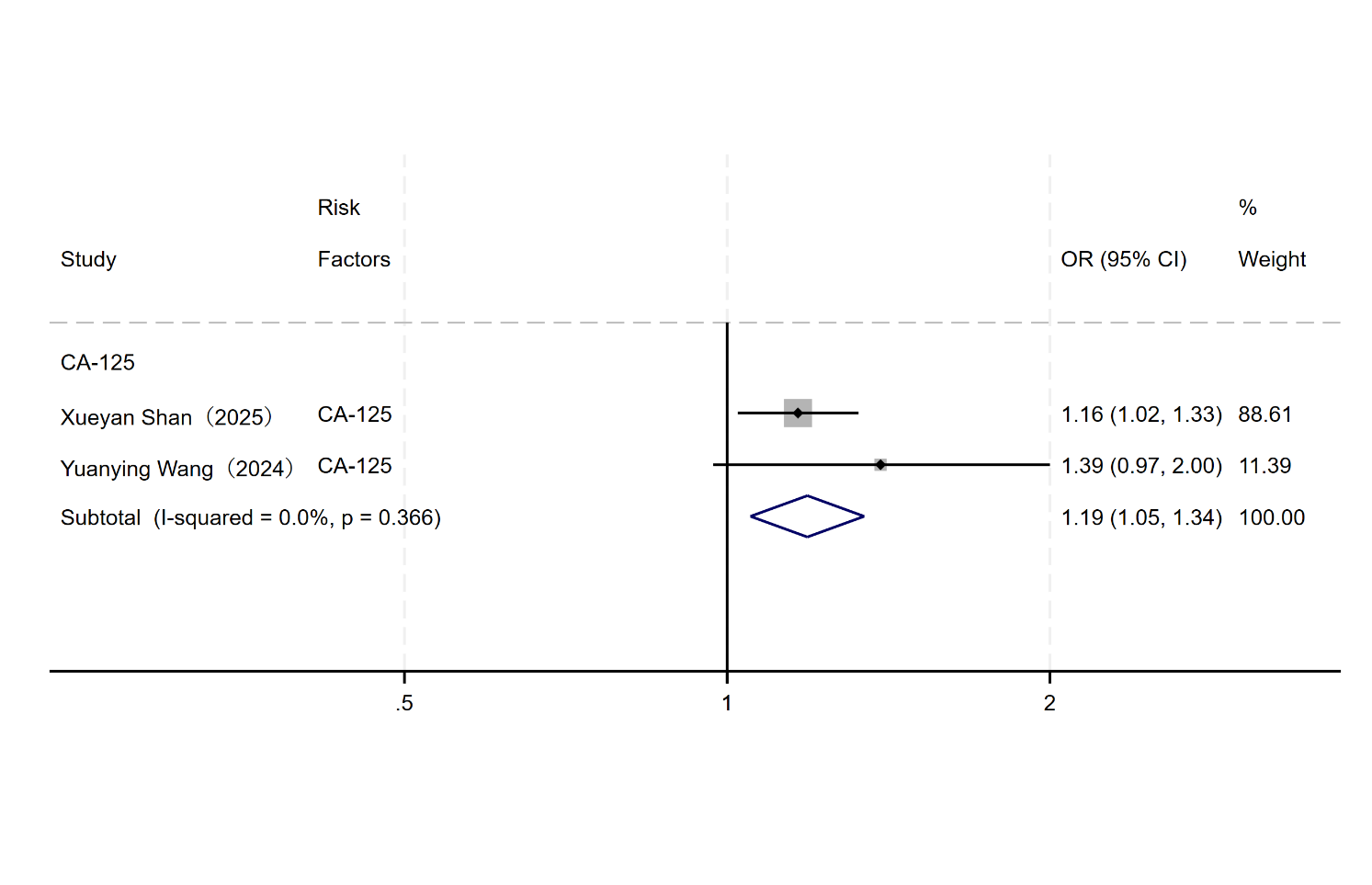


**Figure 12** Forest plot of CA-125 as a risk factor for PPF in CTD-ILD

## Forest Plot of hSP-D


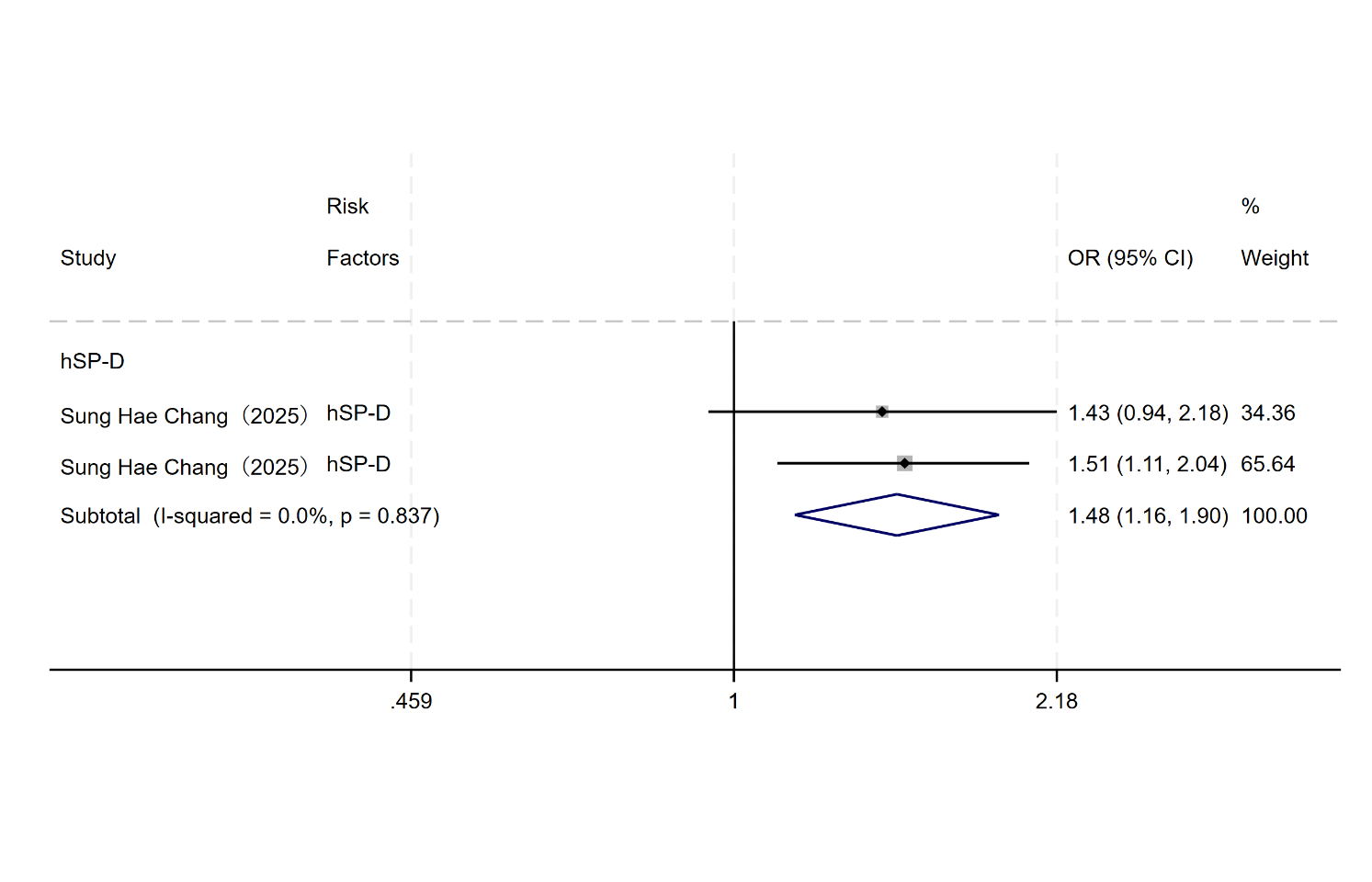


**Figure 13** Forest plot of hSP-D as a risk factor for PPF in CTD-ILD

## Forest Plot of KL-6


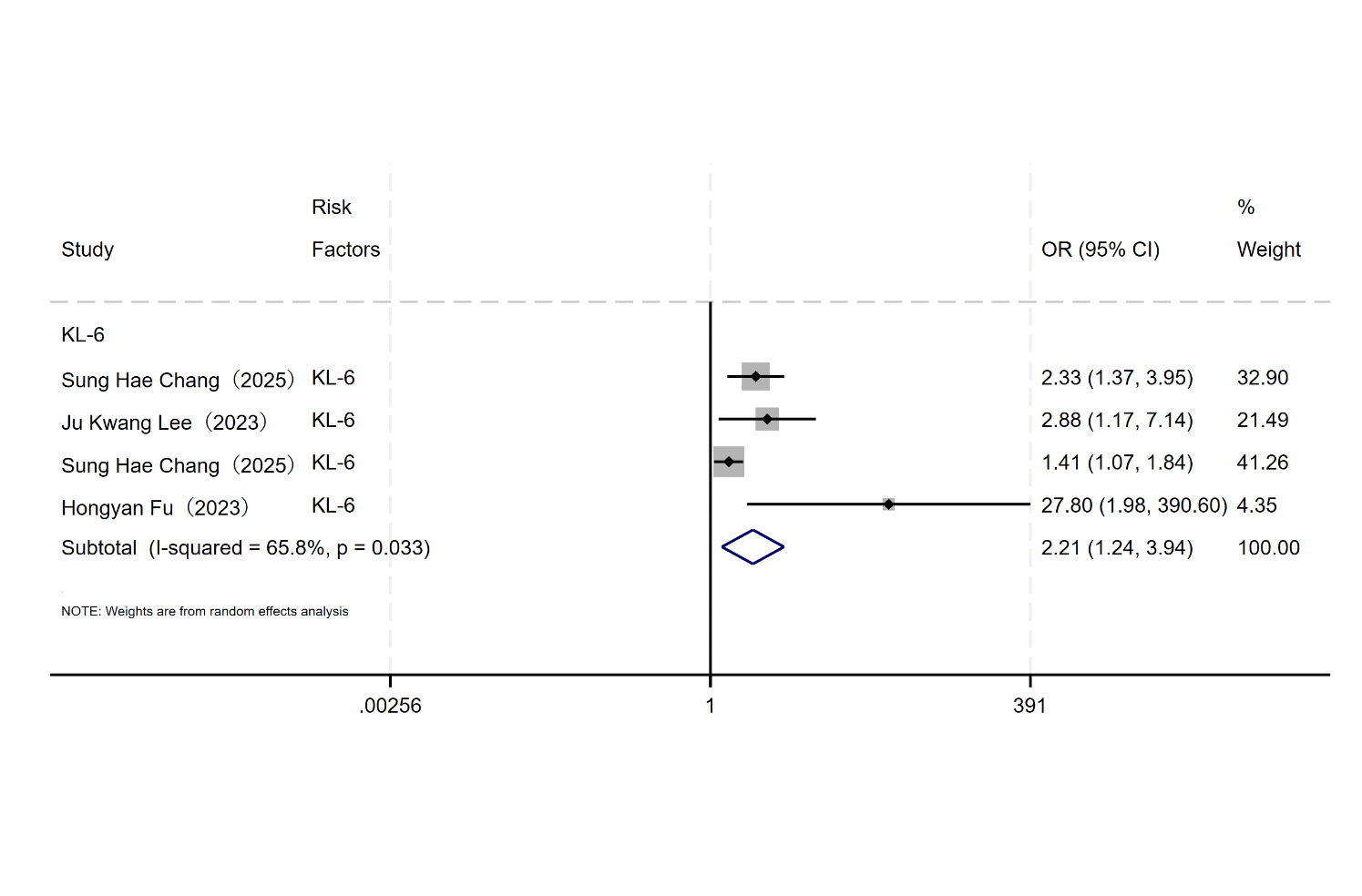


**Figure 14** Forest plot of KL-6 as a risk factor for PPF in CTD-ILD

## Forest Plot of MMP-7


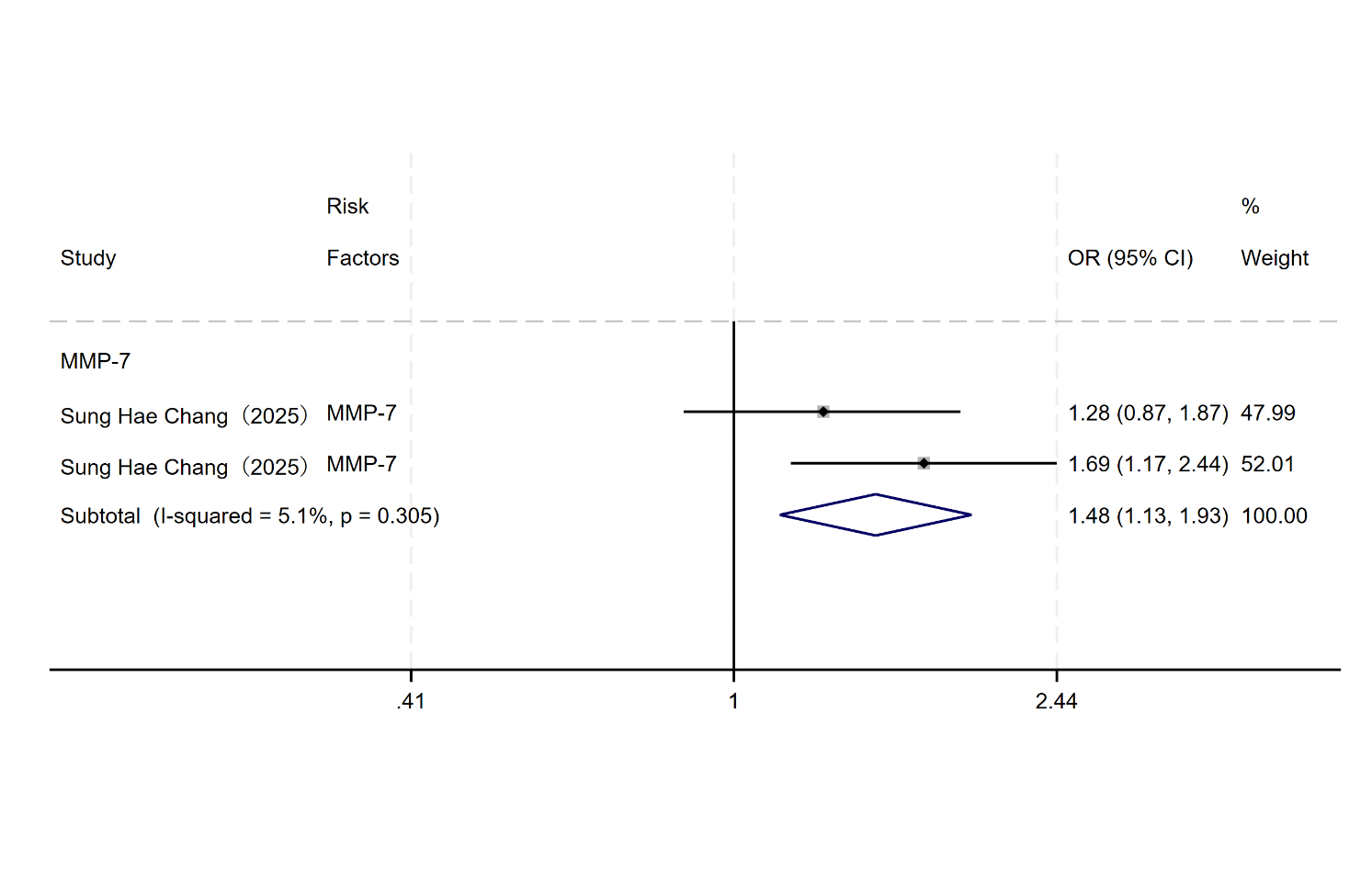


**Figure 15** Forest plot of MMP-7 as a risk factor for PPF in CTD-ILD

## Forest Plot of FVC% predicted


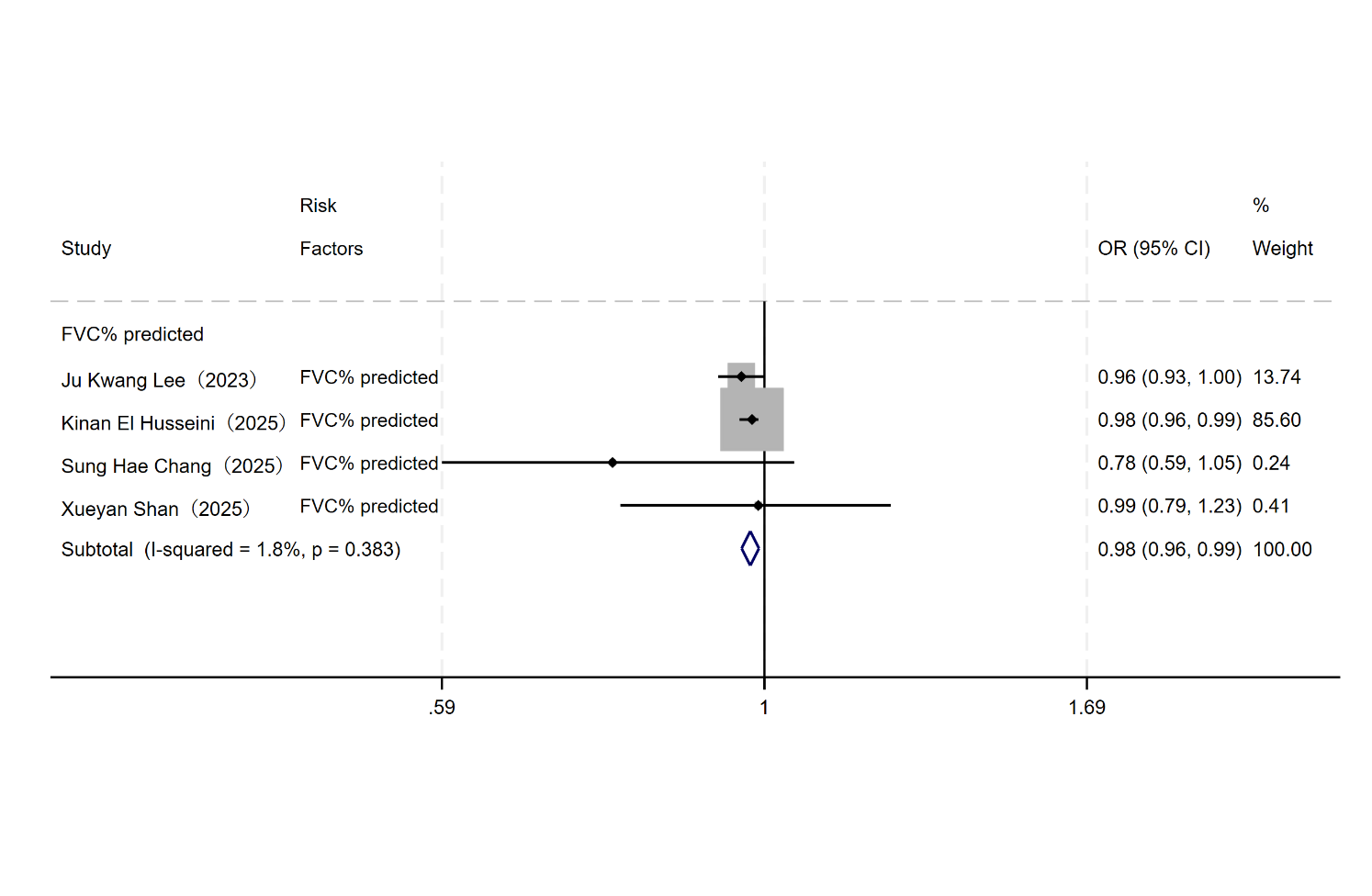


**Figure 16** Forest plot of FVC% predicted as a protective factor for PPF in CTD-ILD

## Forest Plot of CEA


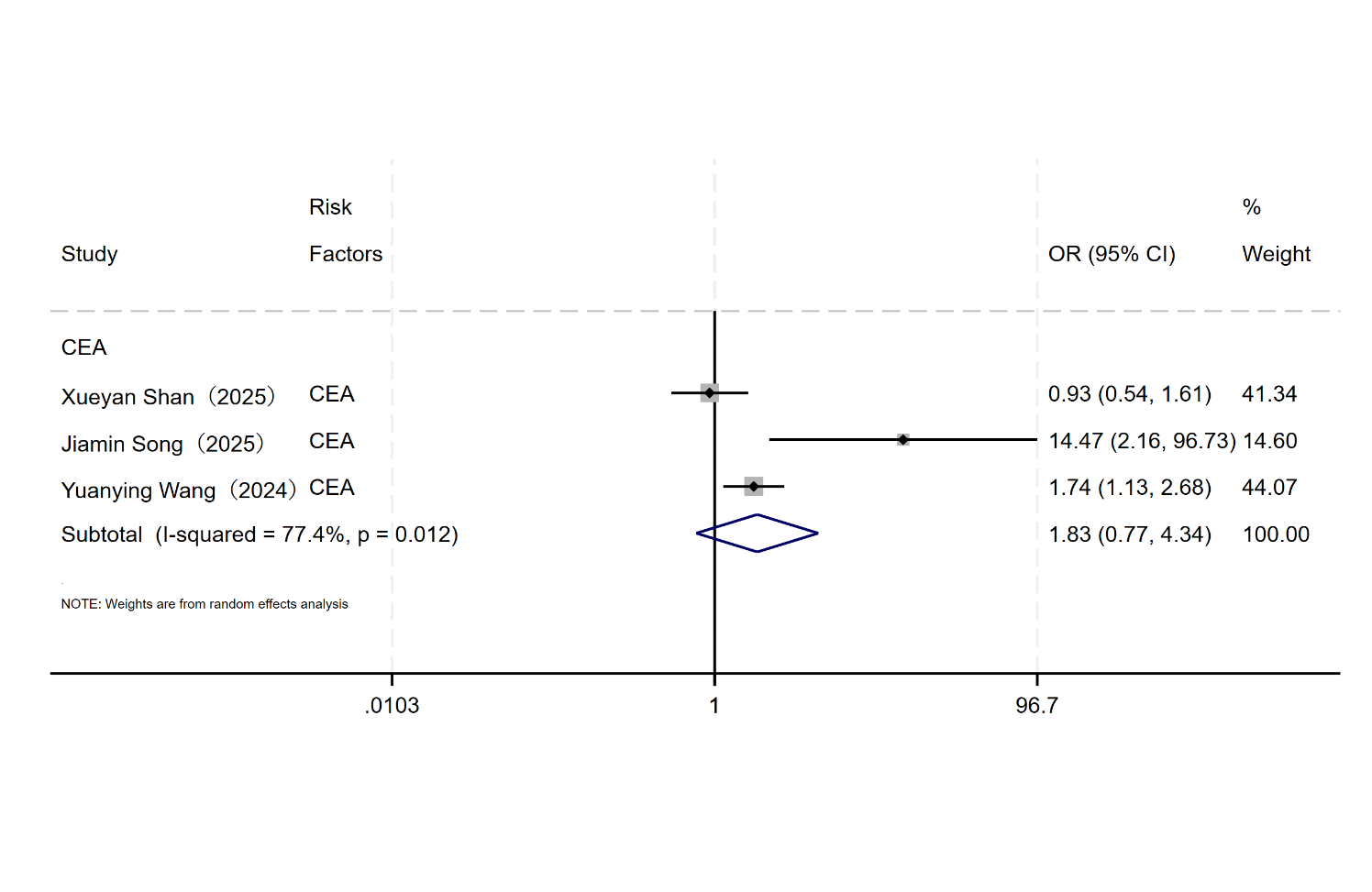


**Figure 17** Forest plot of CEA as an influencing factor for PPF in CTD-ILD

## Forest Plot of DLCO


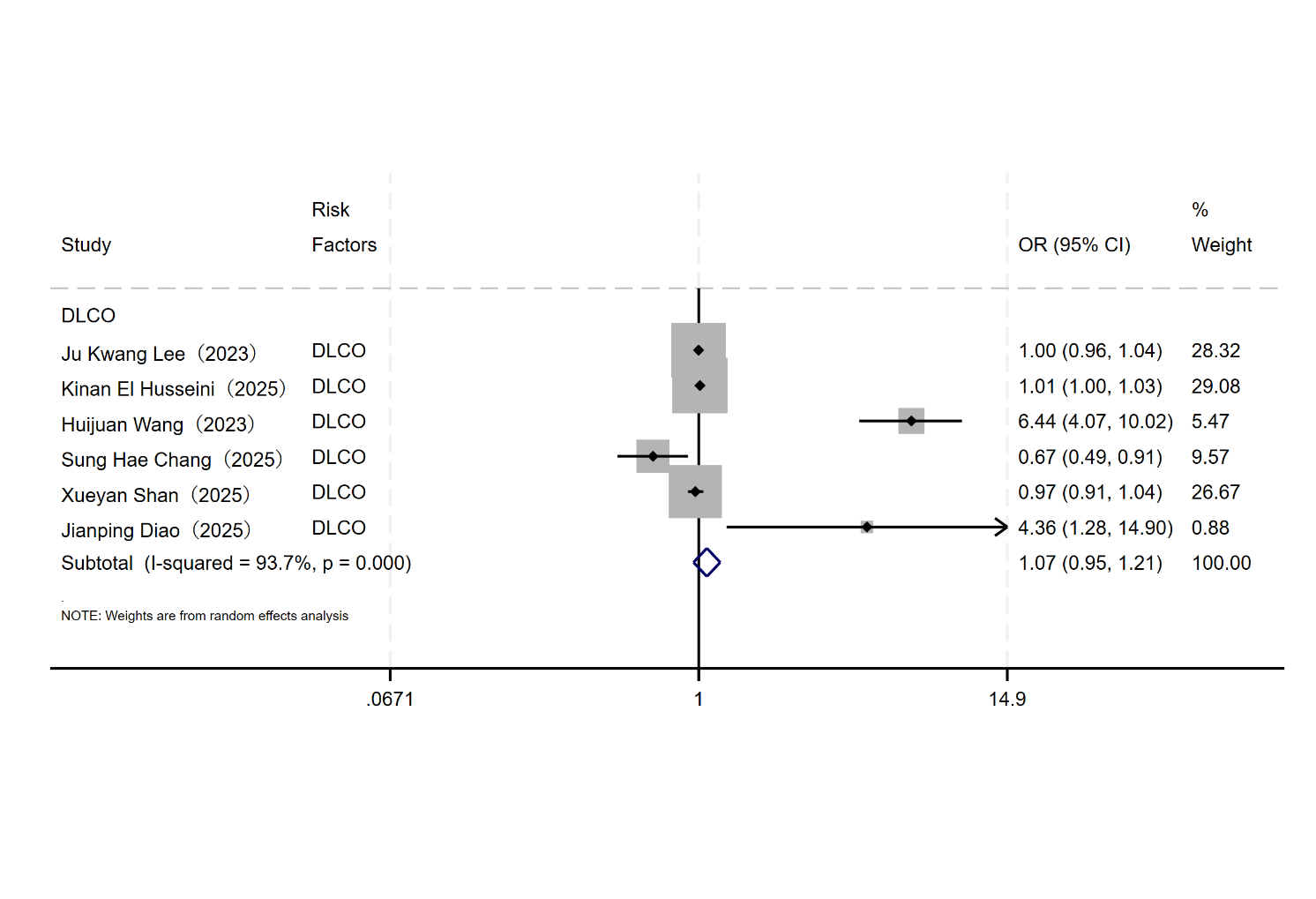


**Figure 18** Forest plot of DLCO as an influencing factor for PPF in CTD-ILD
